# Integrated proteomic and transcriptomic analysis of the *Aedes aegypti* eggshell

**DOI:** 10.1186/1471-213X-14-15

**Published:** 2014-04-05

**Authors:** Osvaldo Marinotti, Tuan Ngo, Bianca B Kojin, Shao-Pei Chou, Brian Nguyen, Jennifer Juhn, Rebeca Carballar-Lejarazú, Pedro N Marinotti, Xiaofang Jiang, Marika F Walter, Zhijian Tu, Paul D Gershon, Anthony A James

**Affiliations:** 1 Department of Molecular Biology and Biochemistry, University of California, Irvine, CA 92697, USA; 2 Department of Molecular Biology and Genetics, Cornell University, Cornell, NY 14850, USA; 3 Department of Biochemistry, Virginia Tech, Blacksburg, VA 24061, USA; 4 Department of Biological Chemistry, School of Medicine, University of California, Irvine, CA 92697, USA; 5 Department of Microbiology and Molecular Genetics, University of California, Irvine, CA 92697, USA

**Keywords:** *Aedes aegypti*, Eggshell, Chorion, Vitelline membrane, Estivation, Oogenesis, Mosquito

## Abstract

**Background:**

Mosquito eggshells show remarkable diversity in physical properties and structure consistent with adaptations to the wide variety of environments exploited by these insects. We applied proteomic, transcriptomic, and hybridization *in situ* techniques to identify gene products and pathways that participate in the assembly of the *Aedes aegypti* eggshell. *Aedes aegypti* population density is low during cold and dry seasons and increases immediately after rainfall. The survival of embryos through unfavorable periods is a key factor in the persistence of their populations. The work described here supports integrated vector control approaches that target eggshell formation and result in *Ae. aegypti* drought-intolerant phenotypes for public health initiatives directed to reduce mosquito-borne diseases.

**Results:**

A total of 130 proteins were identified from the combined mass spectrometric analyses of eggshell preparations.

**Conclusions:**

Classification of proteins according to their known and putative functions revealed the complexity of the eggshell structure. Three novel *Ae. aegypti* vitelline membrane proteins were discovered. Odorant-binding and cysteine-rich proteins that may be structural components of the eggshell were identified. Enzymes with peroxidase, laccase and phenoloxidase activities also were identified, and their likely involvements in cross-linking reactions that stabilize the eggshell structure are discussed.

## Background

Insect eggshells provide embryos protection from environmental insults, both physical and biological [1-3]. Although the primary protective function of eggshells is conserved among insects, differences in ecology, in particular oviposition substrates, are strong forces driving the evolution of eggshell morphology, organization and composition [4-6]. Accordingly, mosquito (Diptera, Culicidae) eggshells show a remarkable diversity in physical properties and structure permitting adaptations to a wide variety of environments. For example, *Aedes aegypti* eggs are highly-resistant to desiccation allowing embryos to survive for months in dry conditions [7,8], while the eggshells of other mosquito species, including anophelines, which comprise human malaria vectors, are more permeable, restricting their development and survival to humid environments [9,10]. Greater knowledge of the proteins that comprise mosquito eggshells is required to understand these differences and to elucidate how they contribute to mosquito reproductive strategies.

Only a small number of *Ae. aegypti* eggshell components have been characterized [11-20]. Here we applied proteomic, transcriptomic, and hybridization *in situ* techniques to identify additional components and describe processes that participate in eggshell assembly. Understanding roles for the discovered proteins in long-term embryo survival may lead to novel approaches for intervening in the reproduction of these mosquitoes and consequently reduce vector-borne transmission of pathogens to humans.

## Results and discussion

### Eggshell proteome

**Figure 1:**
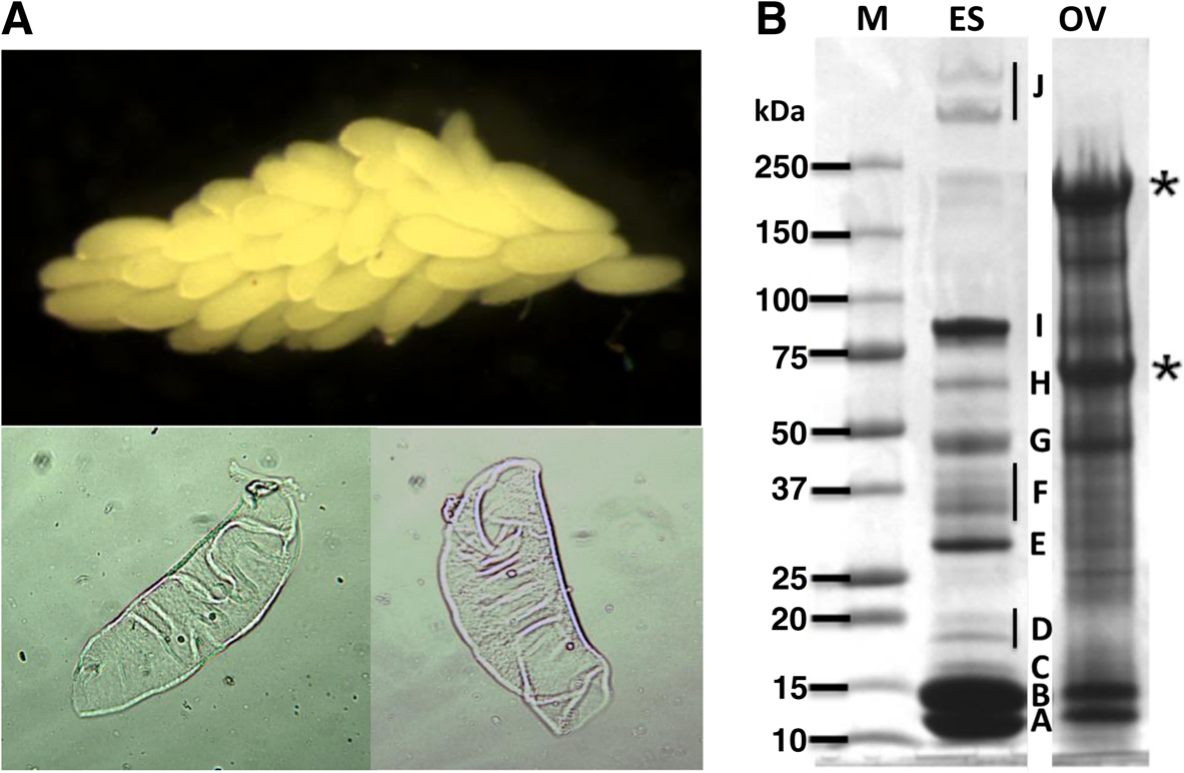
***Aedes aegypti *eggshell purification. A)** Eggshells purified by differential sedimentation from ovaries dissected at 72 hPBM and visualized by light microscopy. Top image, ovary dissected at 72 hPBM. Bottom images, eggshells purified by differential sedimentation. **B)** Ovary (Ov) and eggshell (ES) protein preparations dissolved in SDS/β-mercaptoethanol buffer resolved using 4–15% SDS-PAGE and stained with Coomassie blue R. The electrophoretic migrations of molecular weight markers (M - Precision Plus Protein Kaleidoscope, Bio-Rad Laboratories) and their corresponding molecular weights are indicated at the left of the figure. The excised gel slices submitted to mass spectrometry are indicated by letters A-J, and the identified proteins are listed in Additional file 1. Vitellin subunits are indicated with asterisks.

*Aedes aegypti* eggshells were isolated and their proteins resolved by SDS-PAGE (Figure 1). Several proteins are visibly enriched in the purified eggshell samples when compared with whole ovaries extracts prepared at 72 hours post blood meal (hPBM). Ten gel slices (A-J) containing the most abundant eggshell proteins identified by Coomassie blue staining were analyzed by mass spectrometry. Thirty-four distinct proteins were identified in this analysis (Additional file 1).

The buffer containing SDS/β-mercaptoethanol used for the SDS-PAGE sample preparations did not dissolve all eggshell components since a pellet was recovered by centrifugation of the samples prior to electrophoresis. Protein crosslinking reactions may form higher-order covalent complexes and prevent solubilization of some eggshell proteins, or prevent them from entering the polyacrylamide gel. A second, ‘shotgun’, approach not based on SDS-PAGE was used to mitigate the possible under-representation of proteins in these samples. Tryptic digestion of the whole eggshell preparations followed by mass spectrometry discovered 127 distinct proteins (Additional file 2). All but three of the proteins identified from the excised gel slices were present in the new list of proteins identified from the second experimental approach. Therefore, a total of 130 proteins were identified from the combined mass spectrometric analyses of eggshell preparations. Differences in the two proteomes may arise from various factors including (a) different eggshell preparations, (b) large (≥ 500 kDa) and small (≤ 5 kDa) proteins not resolved during electrophoresis, (c) proteins between gel slices that were not excised after SDS-PAGE, and (d) differences in thresholds parameters for database search results.

### Temporal patterns of transcript accumulation – transcriptome and quantitative RT-PCR

An insoluble fraction was recovered from the eggshell preparation even after treatment with trypsin. Therefore, we undertook a complementary, non-protein based approach to evaluate the complexity of the eggshells. The transcriptome of ovaries at the latter stages of eggshell formation (60-72 hPBM) was compared with the published transcriptome of recently-laid *Ae. aegypti* eggs [21]. Transcripts enriched in the late ovaries and either absent or present at negligible levels in embryos most likely correspond to genes that are expressed in the follicle cells. These are candidates for encoding components of the outermost eggshell layers. Transcripts enriched in the ovaries and maintained at high levels in embryos could be maternally-derived transcripts that serve functions in early embryonic development [22-24].

A total of 536 transcripts were found to be at least 10-fold more represented in ovaries than in embryos (p values < 5E^−05^) (Additional file 3). Publicly-available expression profiles of those transcripts were retrieved and analyzed [25,26] (Additional file 4). We performed quantitative RT-PCR to examine accumulation profiles of selected transcripts to validate the high-throughput derived data (Additional file 5). Quantitative RT-PCR, microarray [25] and RNA-Seq [26] derived data were concordant for the majority (>90%), and evidenced genes with distinct expression patterns.

Not all proteins identified in the eggshell proteome described above were encoded by these transcripts and this is consistent with differential expression profiles of the eggshell components [25,26] (Additional files 4 and 5). For example, vitelline membrane proteins (VPMs) encoding genes are expressed during early oogenesis and were not represented among the abundant transcripts during the late stages of egg formation. Also, a number of the transcripts present in ovaries and absent, or detected at low levels in embryos, encode predicted intracellular components that although displaying differential expression profiles are not likely components of the eggshell.

### Spatial patterns of transcript accumulation - hybridizations *in situ*

**Figure 2:**
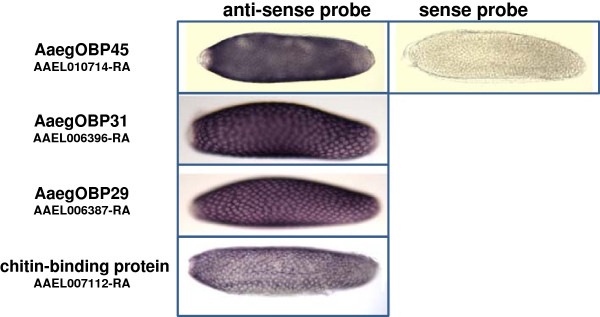
**Hybridizations *in situ *of four transcripts encoding *Ae. aegypti *eggshell proteins.** AaegOBP45 (AAEL01714-RA), AaegOBP31 (AAEL006396-RA), AaegOBP29 (AAEL006387-RA), chitin-binding protein (AAEL007112-RA). All anti-sense probes hybridize to the follicle cells while the sense probe does not. The clear, stain-free, elliptical shapes indicate the positions of the follicular cell nuclei.

Hybridizations *in situ* were performed to further validate expression of four selected genes in vitellogenic ovaries (Figure 2). Three odorant binding protein (OBP) encoding transcripts (AAEL01714-RA, AAEL006396-RA, AAEL006387-RA) accumulated in the follicle cells of ovaries dissected at 48 hPBM. The transcripts of a gene (AAEL007112) encoding a protein with a chitin-binding domain, also accumulated in follicle cells. Hybridizations *in situ* with ovaries dissected later than 48 hPBM to map accumulation of transcripts encoding cysteine-rich proteins (CRPs) failed. Previous attempts to perform hybridizations *in situ* at these late stages of oogenesis also failed [24]. Structural and/or physiological changes in the ovaries during late egg formation could either degrade the probes or block their access to targets within the follicle cells.

### Functional assignments of eggshell components

**Figure 3:**
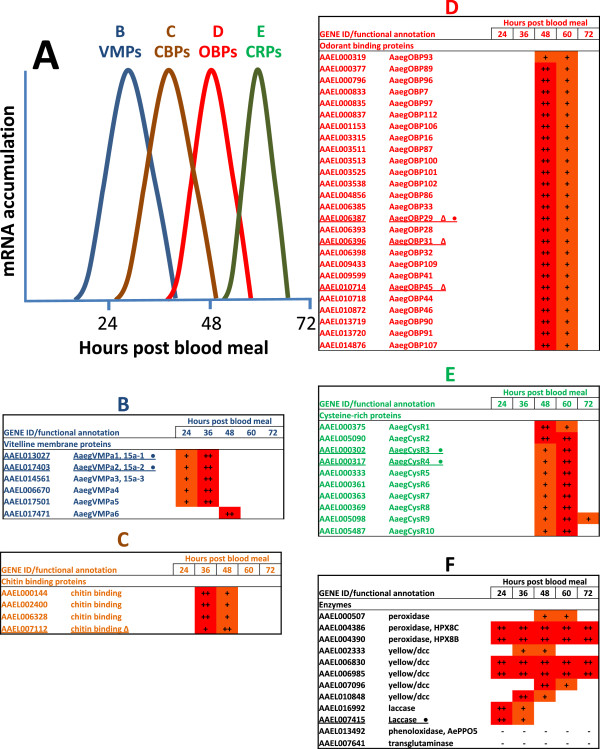
**Functional annotations and expression profiles of *Aedes aegypti *genes associated with eggshell formation.** Expression profiles are derived from qRT-PCR (this publication), microarray [25] and RNAseq data [26]. **A)** Schematic representation of the sequential expression of genes belonging to distinct functional categories: VMPs, vitelline membrane proteins; CBPs, chitin binding proteins/chitinases; OBP, odorant binding proteins; CRP, cysteine-rich proteins. Panels **B**, **C**, **D**, **E** and **F** display lists of annotations and expression profiles for VMPs, CBPs, OBPs, CRPs and enzymes involved in eggshell formation. Selected genes (underlined) had their spatial and temporal expression profiles validated by hybridization *in situ* (indicated by Δ; Figure 2) or quantitative RT-PCR (indicated by ●; Additional file 5). Expression values indicated by ‘++’ and ‘+’ refer to higher and lower relative abundance, respectively, with blanks representing negligible or undetectable levels.

Grouping the transcripts and proteins identified in this study according to known and putative functions and expression profiles reveals multiple components participating in a sequential process of eggshell formation (Figure 3). Morphological aspects of the events resulting in mosquito eggshell formation have been described previously [27-29]. The proteins identified here provide molecular support to the earlier ultrastructural observations. Genes either expressed constitutively or specifically at early, intermediate or late stages of egg formation were grouped in six categories described below, five of which are listed in Figure 3.

#### Vitelline membrane proteins

The vitelline membrane is the inner layer of the eggshell and vitelline membrane proteins (VMPs) are its main components. Three *Ae. aegypti* VMPs, 15a-1, 15a-2 and 15a-3 [15] were recovered from gel slices (A and B) that contain proteins with MW values between 10 and 15 kDa (Figure 1), sizes consistent with their predicted sequences. Gel slices C and D, containing proteins with MWs between 15 and 18 kDa, also contained VMPs 15a-1 and 15a-3, supporting the interpretation that alternative post-translational modification or other factors may result in their abnormal and distinctive SDS-PAGE migration.

**Figure 4:**
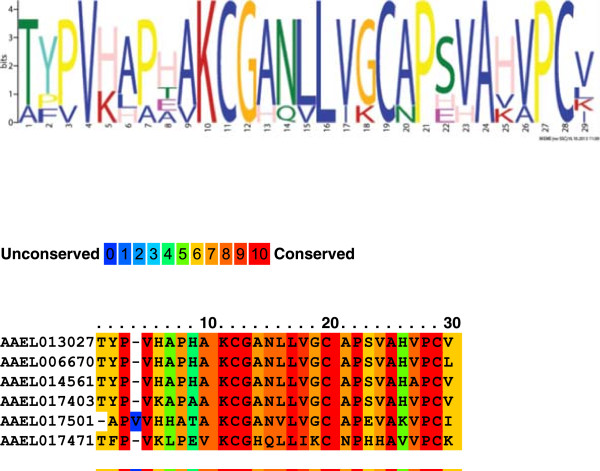
**Conservation of a hydrophobic amino acid sequence motif among *Aedes aegypti *vitelline membrane proteins.** The conserved motif is composed of a sequence 30 amino acids in length. Conserved motif discovery was performed using MEME [65]http://meme.nbcr.net/meme/cgi-bin/meme.cgi.

The products of AAEL006670, identified in gel slice B, and AAEL017471 and AAEL0017501, identified in the whole eggshell proteome, are VMP paralogs and annotated here as novel *Ae. aegypti* VMPs. Two characteristics that define insect vitelline membrane proteins are essential hydrophobic domains that are highly-conserved in all members of the protein family and a short period of expression and accumulation at the onset of egg formation [30]. The three novel *Ae. aegypti* VPMs display hydrophobic domains of 30-50 amino acids in length and their corresponding transcripts are up-regulated following a blood meal, patterns consistent with their proposed function (Figures 3 and 4; Additional files 4, 5 and 6). AAEL017471-RA is unique among the *Ae. aegypti* VMP-encoding transcripts in that its accumulation was maximum at 48 hPBM, while all other VMP transcripts attained their highest levels at 24-36 hPBM. A similar pattern was observed with one of the *D. melanogaster* VMPs, VM32E, which is expressed only at stage 10 of oogenesis and considered a ‘late’ vitelline membrane gene [31]. VM32E is an integral component of both the vitelline membrane and the endochorion and it may serve as a bridge between the two eggshell layers [31]. Therefore, VM32E and AAEL017471 could perform comparable functions.

#### Proteins with chitin-binding and chitinase-like domains

Chitin is present in several insect structures at various developmental stages. In addition to the exoskeleton, chitin and chitin-like materials are found in the peritrophic matrix (PM), embryos, ovaries, compound eyes and mosquito eggshells [32-34]. Proteins with chitin-binding domains and/or chitinase domains (CBPs), products of AAEL006328, AAEL002400, AAEL000144, and AAEL007112, are included in our datasets of eggshell components (Figure 3, Additional files 1 and 2). AAEL002400-PA and AAEL000144-PA are present in gel slices H (Molecular Weight, MW ~ 60 kDa) and J (250 kDa) (Figure 1), possibly a result of their association with higher-order covalent complexes.

Feeding the chitin synthesis inhibitor lufenuron to adult female mosquitoes results in inhibited oogenesis and egg hatching. Furthermore, eggs laid by lufenuron-treated mosquitoes are fragile [33] and this supports an important role for chitin in maintaining egg integrity. Our findings of chitin-binding proteins in *Ae. aegypti* eggshells support the hypothesis that an organized structure composed of chitin and chitin-binding proteins provides rigidity to the mosquito eggshell. Eggshell proteins with chitinase domains may play a role in embryo hatching [33,35] and be stored as proenzymes that are activated immediately prior to hatching.

#### Odorant binding proteins

Gel slices E, F and G, containing proteins migrating with apparent MWs between 30 and 48 kDa (Figure 1) and the shotgun proteomic approach revealed a series of odorant binding proteins (OBPs) as eggshell components (Additional files 1 and 2). Insect OBPs are reported to be expressed in insect antennae and are proposed to bind small hydrophobic odorant molecules and carry and present them to the olfactory receptors [36]. Structurally-related proteins also have been identified in non-sensory organs of insects, supporting the conclusion that OBPs and OBP-like proteins could have roles unrelated to olfaction. For example, D7-related (D7r) proteins similar in structure to OBPs are abundant components in the saliva of hematophagous insects. The *An. gambiae* D7r proteins have been shown to bind the biogenic amines serotonin, norepinephrine, and histamine with high affinity thereby reducing the concentrations of these effectors at the feeding site and resulting in an anti-hemostatic activity [37]. The *Tenebrio molitor* THP12 gene encodes a member of the OBP family that is accumulated in the hemolymph and has the capacity to bind fatty acids with high affinity, a finding consistent with a role in the transport of small hydrophobic molecules [38].

All OBPs identified in the *Ae. aegypti* eggshell preparations, with the exception of AAEL011483-PA (AaegOBP 50c), belong to the atypical, two-domain subfamily or two-domain clade of OBPs [39,40]. As the nomenclature indicates, proteins in this subfamily have two OBP domains that likely originated from the fusion of two Classic OBP genes. AAEL011483 encodes a protein that belongs to the “PlusC” OBP family, however its expression pattern indicates that it is not expressed in the ovaries (Additional file 4). AAEL000319-RA (AaegOBP93), although expressed in ovaries, showed enhanced expression in larvae and likely has an alternative function. Consistent with our findings, six of eight OBPs identified in the eggshell of *An. gambiae*[41] also belong to the atypical, two-domain subfamily or two-domain clade. Although little is known about natural ligands of the OBPs expressed and accumulated in the ovaries, they could carry substrates for eggshell protein crosslinking and assembly reactions. Alternatively or additionally, odorant molecules may act as chemo-attractants for sperm [42], and the OBPs identified in this study could enrich specific attractant molecules at the surface of the untanned mosquito eggshell.

The accumulation of OBPs in vitellogenic mosquito ovaries evidenced by immunoblot analyses [43], their high abundance in the eggshell demonstrated by SDS-PAGE and proteomics in this study, and their transcript accumulation patterns support the hypothesis that they may be structural components of the chorion intermediate layer. OBPs delivering crosslinking substrates could become trapped within the eggshell structure and may have been co-opted through evolutionary selection processes for additional functions.

#### Cysteine-rich proteins - putative chorion proteins

**Figure 5:**
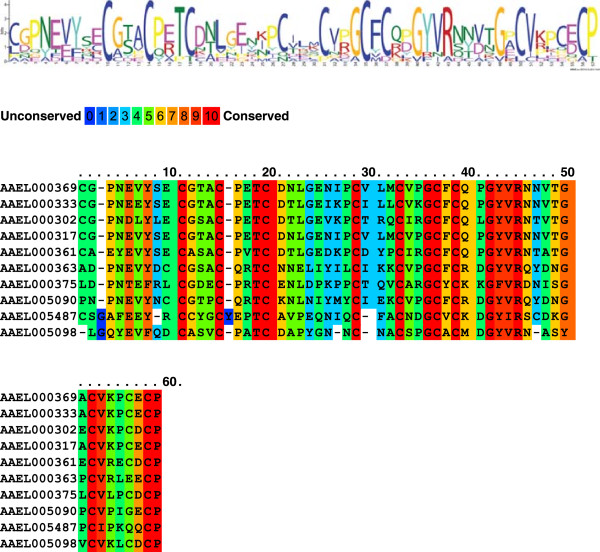
**Identification and alignment and of a motif 59 amino acids in length that is conserved among the *Aedes aegypti *eggshell cysteine-rich proteins.** Conserved motif discovery was performed using MEME [65]http://meme.nbcr.net/meme/cgi-bin/meme.cgi.

A group of small proteins displaying high cysteine content were described as putative *An. gambiae* chorion proteins [41]. Similarly, we found 10 cysteine-rich proteins (CRPs) as components of the *Ae. aegypti* eggshell. They compose a group of short, putatively secreted proteins that contain a trypsin inhibitor-like, cysteine-rich domain (IPR002919). The genes encoding these proteins are among those expressed most highly in ovaries at 60-72 hPBM and their transcripts are absent from embryos. This pattern supports their function as components of the most external layers of the chorion deposited during the last stages of oogenesis. An alignment of the eggshell CRPs sequences revealed a conserved motif 59 amino acids in length that defines a hydrophobic domain possibly analogous to that seen in the vitelline membrane proteins (Figure 5, Additional file 7).

Chorion proteins are proposed to have evolved rapidly, providing adaptation to stresses such as desiccation, plant toxins, pathogen invasion and predation [4]. Culicidae (mosquito) and Drosophilidae (fruit fly) lineages are estimated to have diverged ∼ 250 million years ago [44] and their females lay eggs in ecologically-distinct sites. The strong selective pressures on chorion components resulting from their respective life histories provide opportunities for engagement of distinct genes and gene families in the process of eggshell assembly. We could not identify any mosquito eggshell component with amino acid sequence similarity to the well-characterized *D. melanogaster* chorion proteins [3]. The mosquito CRPs possibly function as structural components of the external chorionic layer of the eggshell and their gene families have many members in both *Ae. aegypti* and *An. gambiae*. Interestingly, the genes encoding eggshell CRPs in *Ae. aegypti* are not annotated as orthologous to those described previously as encoding *An. gambiae* putative chorion proteins [41].

#### Enzymes

Peroxidases, phenoloxidases, laccases, dopachrome-conversion enzymes, and transglutaminases are enzymes present in the eggshell samples (Figure 3, Additional files 1, 2 and 3). Peroxidase-catalyzed chorion protein crosslinking is a phenomenon documented previously in insect eggshell formation [45] with AAEL004386-PA, HPX8C, identified in the proteome, having been characterized previously as an *Ae. aegypti* chorion peroxidase (CPO) [18]. The peroxidases AAEL004390-PA (HPX8B) and AAEL000507-PA also were present in our datasets (gel slices H and I, respectively) likely representing new enzymes involved in eggshell formation. Laccase (AAEL016992) was identified as an *Ae. aegypti* eggshell component. Laccases have p-diphenol oxidase enzymatic activity and participate in cuticular tanning in insects. Pan *et al*. [46] reported that *Culex pipiens pallens* laccase 2 (CpLac2) is expressed abundantly in the egg developmental stages and postulated a role in eggshell tanning. The presence of the *An. gambiae* Laccase 2 transcripts (AGAP006176-RA and -RB) in ovaries and corresponding peptides in the eggshell provide additional support for this hypothesis [41]. Pigmentation of *Ae. albopictus* eggshells was blocked when laccase 2 expression was knocked down by RNAi [47]. Furthermore, eggs produced after laccase 2 knockdown showed incomplete sclerotization, were deformed and did not complete development [47].

Phenoloxidases are involved in chorion melanization [12]. AePPO5 (AAEL013492-PA) is present in *Ae. aegypti* eggshells (Additional file 2), however, the accumulation profile of its corresponding transcript indicates that the enzyme is synthesized mainly in tissues other than ovaries [26] (Additional file 4). AAEL013492 is an ortholog of the *An. gambiae* prophenoloxidase 9 (AgPPO9; AGAP004978-PA) and the *Cu. quinquefasciatus* product of CPIJ016564 [19]. AePPO5 and AgPPO9 lack secretory signal peptides, and mechanisms involved in the transport of these enzymes to the eggshell are unknown. A putative transglutaminase (AAEL007641-PA) identified as a component of the eggshell (Additional file 2) also has an expression pattern inconsistent with its synthesis in the ovaries [26] (Additional file 4). Transglutaminases participate in multiple biological processes by catalyzing protein crosslinking and playing a key role in cuticle morphogenesis and sclerotization in insects [48]. Therefore, AAEL007641-PA may catalyze protein cross-linking during *Ae. aegypti* eggshell formation.

The translation products of AAEL006985, AAEL006830, AAEL007096, AAEL010848, and AAEL002333 were identified in gel slices F and H and contain motifs similar to those found in the Major Royal Jelly protein family. This protein family includes the products of the *Drosophila yellow* genes, which encode dopachrome-conversion enzymes that catalyze cuticle melanization. The *yellow-g* and *yellow-g2* gene products participate in the cross-linking of fruit fly eggshell proteins [49]. Those and our proteome and gene expression data support the hypothesis that mosquito Major Royal Jelly proteins play a catalytic role in the crosslinking of eggshell proteins. However, dopachrome conversion enzyme-encoding genes also are expressed in tissues other than ovaries and at various developmental stages, supporting a role for them in other mosquito metabolic functions. Our proteome data did not include AAEL014238-PA, a dopachrome-conversion enzyme described previously as participating in *Ae. aegypti* eggshell formation [17]. Its absence indicates that the eggshell protein set reported here, although larger than those included in the previous reports, may be incomplete.

#### Other cellular and structural proteins

Proteins with similarity to cytoplasmic or cell membrane components were identified in eggshell preparations and could indicate novel functions for these molecules (Additional files 1 and 2). Actin (AAEL001673-PA), gamma glutamyl transpeptidase (AAEL004240-PA), transcriptional regulator ATRX (AAEL010502-PA), ATP-binding cassette transporter (AAEL012192-PA), vacuolar sorting protein (AAEL005861-PA), glycosyl transferase (AAEL001590-PA), coronin (AAEL009249-PA), alpha (1,3) fucosyltransferase (AAEL000244-PA), and a protein of unknown function (AAEL002382-PA) were identified by the proteomics approach. However, a number of non-eggshell proteins may co-purify with the eggshells with the methods used in this study. Other molecules with putative functions in eggshell formation identified by our differential transcriptomic methodology include serine-type endopeptidases (AAEL014188-PA, AAEL003060-PA), a putative 67B2 family heat shock protein (AAEL007339-PA) and myosin (AAEL005733-PA). Although actin, myosin, heat shock proteins and serine proteinases also were identified in *D. melanogaster* eggshell preparations [50], their functions in the mosquito eggshell remain to be validated.

## Conclusions

*Aedes aegypti* is an important world-wide vector of dengue and yellow fever viruses. Therefore, the management of *Ae. aegypti* populations is of prime importance to public health and welfare. In general, *Ae. aegypti* population densities are low during cold and dry seasons and increase immediately after rainfall. This pattern is due to the hatching of a large number of estivated, drought-resistant larvae that hatch in response to water accumulation in natural and artificial breeding sites. Mosquito survival until the next favorable climatic period is then a key factor for the persistence of *Ae. aegypti* populations [51]. Thus, targeting *Ae. aegypti* eggs to make them less resistant, or intolerant to desiccation would be beneficial.

Our data fill a gap in knowledge regarding the proteins and sequential processes involved in *Ae. aegypti* eggshell formation. Proteins involved in the hardening and tanning processes of the mosquito eggshell may represent targets for the development of enzyme-specific inhibitors that added to the egg-laying sites and/or fed to females via blood or nectar could result in abnormal egg formation or a defective chorion. For example, phoroglucinol, a natural phenolic peroxidase inhibitor, affects eggshell formation when fed to adult female insects [52]. This substance or derivatives are potential candidates for controlling vector populations in integrated insect control programs. High-throughput searches for molecules with inhibitory activity on enzymes [53,54] also can be applied to insect control. The discovery or development of highly specific, *Ae. aegypti* eggshell enzyme inhibitors would be advantageous and desirable to minimize unwanted environmental effects of insect control strategies on non-target organisms. Design of insecticides that target cuticle and chorion formation, such as lufenuron used to control fleas [55], are possible with an understanding of the biochemical and physiological processes involved in eggshell formation in mosquitoes.

The data documented here also support control strategies involving transgenic insects [56-59]. Transgenic mosquitoes with a drought-intolerant embryo phenotype could be engineered, easily raised under controlled humidity laboratory conditions, and upon release would display little or no fitness cost during rainy seasons. However, their eggs would not survive through dry seasons. The population suppression proposed here together with other efforts that result in local or regional vector elimination are anticipated to delay the onset of increased mosquito populations that follows the onset of a rainy season, result in reduced pathogen transmission and provide a consequent reduction in human morbidity and mortality.

## Methods

### Mosquitoes

The *Ae. aegypti* Liverpool strain was maintained at 26°C ± 1°C with 77% humidity and 12 h day/night, 30 min dusk/dawn lighting cycle. Larvae were fed a diet of powdered fish food (Tetramin). Adult males and females were kept together in a cage with unlimited access to water and sugar (10% sucrose) until blood feeding. Mosquitoes aged 3-5 days after eclosion were allowed to feed on anaesthetized mice. The study was carried out in strict accordance with the recommendations in the Guide for the Care and Use of Laboratory Animals of the National Institutes of Health.

### Eggshell isolation

Eggshell isolation was carried out as described [20] with modifications. Ovaries were dissected from females at 72 hPBM in 0.15 M NaCl and homogenized in a HEPES buffer, pH 8.0 (10 mM HEPES, 130 mM NaCl, 4.7 mM KCl, 0.5 mM phenyl thiocarbamide, 0.1 mM p-nitrophenyl-p’-guanidinobenzoate, 5 mM ethylene diamine tetraacetic acid, 1% Triton X-100, 0.1 mM diethyldithiocarbamic acid, 1 mM phenylmethylsulfonyl fluoride). Eggshells were collected by sedimentation for 15 min on ice and the supernatant decanted. The sediment was washed five times for 15 min each with homogenization buffer. Eggshells then were sonicated for 30 seconds and centrifuged (100 g, 10 min, 4°C). Five additional cycles of resuspending the pellet in homogenization buffer followed by centrifugation were performed to remove cellular contaminants.

### SDS-PAGE/mass spectrometry

Purified eggshell and whole ovary samples were dissolved in SDS/β-mercaptoethanol for 2 min at 100°C and then resolved by polyacrylamide gel electrophoresis. Proteins were stained with Coomassie Brilliant Blue R-250 and excised as slices from the gel using a sharp scalpel. Gel slices were destained in 100 μl of 100 mM ammonium bicarbonate and 50% acetonitrile for 10 min at room temperature (RT) while agitating at 700 rpm. Two identical destaining steps were performed. The gel slices were dehydrated in 30 μl of acetonitrile for 10 min at RT, while agitating at 700 rpm. The acetonitrile was removed and the dehydration step was repeated. After removal of the acetonitrile, gel slices were dried in a Savant ISS110 SpeedVac Concentrator for 10 min at 45°C. Disulfide bonds were disrupted in 50 μL of 10 mM dithiothreitol in 50 mM ammonium bicarbonate, incubating for 1 hr at 60°C. The dithiothreitol solution was removed and cysteines were alkylated with 50 mM iodoacetamide in 50 mM ammonium bicarbonate during incubation for 45 min at 45°C in the dark. Gel slices then were washed with 20 μl of 50 mM ammonium bicarbonate, followed by a wash with 50 μl of 100 mM ammonium bicarbonate. After two 10 min washes with 30 μl of acetronitrile, gel slices were dried for 5-10 min under vacuum. Each sample was incubated in trypsin (50 μL at 20 ng/mL) for 45 min at 4°C and then at 37°C, overnight. Tryptic peptides were extracted by adding 30 μL of 50% (v/v) acetonitrile/1% (v/v) formic acid and incubating for 10 min at RT. The extraction solution was transferred to another tube and the extraction step was performed three additional times. All extracts of each gel slice were combined and samples evaporated to dryness in a rotary vacuum and reconstituted in 20 μL of HPLC loading buffer consisting of 50% (v/v) acetonitrile/1.0% (v/v) formic acid. LC-MS was carried out for data acquisition using a Waters ACQUITY UPLC® system coupled to a SYNAPT®G2 mass spectrometer. Injected samples were washed on a trap column prior to being loaded on a separation column. The sample was separated by a linear gradient from 95% A (0.1% v/v formic acid in water) and 5% B (0.1% v/v formic acid in acetonitrile) to 65% A and 35% B over 90 min. Masslynx 3.5 software (Waters Corporation, Milford, MA, USA) was used for data acquisition, and processing. Peptides were identified using ProteinLynx Global Server Web (Waters) with SwissProt database for *Ae. aegypti*.

### Whole eggshell nanoLC-MS/MS

Protein extraction and digestion for mass spectrometry was adapted from [60]. Briefly, eggshells were dissolved in a buffered solution containing sodium dodecyl sulfate (SDS) and tris(2-carboxyethyl)phosphine (TCEP) (Pierce) and the resulting solution placed on a 30 kDa ultrafiltration unit (YM-30, Millipore) which was used for buffer exchange, carbamidomethylation of cysteine, and protein trypsinization. Eluted peptides were cleaned in pipette tips packed with strong cation-exchange (SCX) and C18 filters (3 M Inc.) [61]. Peptides eluted from the C18 phase were transluted to the SCX phase to remove remaining contaminants. Peptides were eluted with ammonium acetate and subjected to nanoLC-MS/MS on an LTQ Velos Pro (ThermoFisher) with EASY-nLC 1000 running a 3 hr acetonitrile gradient at a flow rate of 0.2 nL/min. The Velos Pro instrument method fragmented the 20 most-abundant ions per precursor spectrum via collision induced dissociation, with dynamic exclusion. Searches using Mascot 2.4 were against an *Ae. aegypti* custom database (alongside a database of common contaminants), with trypsin specificity. The sole fixed and variable modifications were Carbamidomethyl (C) and Oxidation (M), respectively. Peptide and fragment mass tolerances were ± 2 Da and ± 0.8 Da, respectively, with a maximum of one missed cleavage.

### Transcriptome analyses

Ilumina transcriptomes were generated as in [62]. Data from two samples, 0-2 hour embryos [19] and ovaries dissected 60-72 hPBM (this work), were compared. The 0-2 hr embryo sample has 6,629,888 reads 33 bp in length, and the 60-72 hPBM ovary sample comprises 15,691,856 40 bp long reads. Reads were aligned by bowtie [63] to the 18,769 annotated *Ae. aegypti* transcripts (AaegL1.3 database, http://vectorbase.org/) to calculate relative transcript accumulation. The number of reads mapped to each transcript was counted and normalized, resulting in values expressed as reads per kilobase per million mapable reads (RPKM).

The DEGseq method in the DEGseq package was used to determine the significance and fold-changes of transcript abundance between samples. The MA-plot-based method with random sampling model (MARS) with the default p-value < 0.001 was used for our analysis. The input data were the values of normalized mapped reads for each transcriptome sample.

### Quantitative RT-PCR

RNA samples were extracted using TRIZOL (Invitrogen) from pools of ovaries dissected from females kept exclusively on a sugar diet or collected at defined intervals after blood feeding. Following DNAse I (Invitrogen) treatment, a total of 10 μg of RNA were used for cDNA synthesis with superscript III (Invitrogen) and random primers. Real-time quantitative PCR reactions of 20 μl were performed in triplicate with SYBR Green Supermix (Biorad) and 0.3 μM of each primer (Additional file 8) on three sequential five-fold dilutions each of the original cDNA. Real-time quantitative PCR reactions were run on an iQ3 system (Biorad). No primer dimers were detected when inspecting the melting curves and primer pairs with at least 90% amplification efficiency were selected. Fold-changes in mRNA abundance were derived by the comparative Ct method [64] using a constitutively-expressed gene, *rp49* (GenBank Acc. No.:AY539746; AAEL003396) as the reference transcript.

### Hybridization ***in situ***

Hybridizations *in situ* in whole-mount ovaries were conducted as described previously [23]. A One-step RT-PCR kit (Qiagen, Valencia, CA) was used to generate the probes, for subsequent cDNA amplification reactions. Sequences of the oligonucleotide primers are listed in Additional file 8. Reaction mixtures were incubated for 30 min at 50°C and 15 min at 95°C. Amplification conditions were 3 min at 95°C followed by 30 cycles of 30 s at 95°C, 30 s at 60°C and 1 min at 72°C. RT-PCR products were cloned into the pCR®4-TOPO® cloning vector (Invitrogen) and sequenced to confirm their identity. Digoxygenin (DIG)-labeled sense and antisense RNA probes for each gene product was synthesized *in vitro* using T3 or T7 RNA polymerases (Ambion, Austin, TX).

## Competing interests

We declare that we have no significant competing financial, professional or personal interests that might have influenced the performance or presentation of the work described in this manuscript.

## Authors’ contributions

OM, PDG and AAJ conceived and designed the experiments. OM, TN, BBK, SPC, BN, JJ, RCL, PM, MFW and PDG performed experiments. OM, TN, JJ, RCL, XJ, ZT, PDG and AAJ analyzed the data. TN, XJ, JJ, ZT and PDG contributed reagents/materials/analysis tools. OM, JJ, PDG, ZT and AAJ wrote the manuscript. All authors read and approved the final manuscript.

## Supplementary Material

Additional file 1 **Identification of *Aedes aegypti *eggshell proteins fractionated by SDS-PAGE and excised from a polyacrylamide gel shown in Figure** 1**.** Click here for file

Additional file 2 **Identification of proteins solubilized by trypsin digestion of *Aedes aegypti *** **eggshells.** Click here for file

Additional file 3 **Comparative analysis of *Aedes aegypti *** **late ovaries (60-72 hPBM) and early embryos (0-2 h) transcriptomes.** Click here for file

Additional file 4 **Expression profiles of selected *Aedes aegypti *eggshell- related genes.** RNAseq data extracted from Akbari *et al*., 2013 show transcript abundance (FPKM) at developmental stages and dissected tissues, including ovaries. Microarray data (Dissanayake *et al*., 2010) are presented as an inset when available, and represent the abundance of specific mRNAs in samples derived from non-blood fed whole females (NBF) and those at 3, 12, 24, 48, 72, and 96 hours following a blood meal (BF). A sample from adult males (M) also is included. Click here for file

Additional file 5 **Quantitative RT-PCR determinations of transcript accumulation in the ovaries of *Aedes aegypti *blood-fed females.** Bars represent the mean of three biological replicates with similar results. RNA for each biological replicate was isolated from the ovaries of a single female. AAEL007415 (Laccase), AAEL013027 (VMP 15a-1), AAEL017403 (VMP 15a-2), AAEL006387 (AaegOBP29), AAEL000302 (Cysteine-rich protein, AaegCysR3), AAEL000317 (Cysteine-rich protein, AaegCysR4). Values were normalized to the expression of the ribosomal protein 49 gene, AAEL003396. Click here for file

Additional file 6 **Sequence and hydrophobicity conservation among *Aedes aegypti *vitelline membrane proteins.** A) Full-length protein sequences were submitted to MEME [65]http://meme.nbcr.net/meme/cgi-bin/meme.cgi and a conserved motif 30 amino acids in length was identified. B) Multiple alignment of the sequence motif constructed at PRALINE [66]http://www.ibi.vu.nl/programs/pralinewww/ provides additional support for its conservation. C) The hydrophobicity patterns of the six vitelline membrane proteins were predicted at ExPASy [67]http://web.expasy.org/cgi-bin/protscale/protscale.pl using the Hphob./Kyte & Doolittle option [68]. Bars were inserted in the graphs to indicate the positions of the conserved motif displayed in A and B. Click here for file

Additional file 7 **Sequence and hydrophobicity conservation among *Aedes aegypti *eggshell cysteine-rich proteins.** A) Full length protein sequences were submitted to MEME [65]http://meme.nbcr.net/meme/cgi-bin/meme.cgi and a conserved motif 50 amino acids in length was discovered. B) Multiple alignment of the sequence motif constructed at PRALINE [66]http://www.ibi.vu.nl/programs/pralinewww/ supports the proposed conservation. C) The hydrophobicity patterns of the cysteine-rich proteins were predicted at ExPASy [67]http://web.expasy.org/cgi-bin/protscale/protscale.pl using the Hphob./Kyte & Doolittle option [68]. Bars were inserted in the graphs to indicate the positions of the conserved motif displayed in A and B. Click here for file

Additional file 8 **Oligonucleotide primer sequences used in quantitative RT-PCRs and for the generation of probes for hybridizations *in situ *** **(q and h suffixes, respectively).** Click here for file
