# Supplementary material for: Integrated proteomic and transcriptomic analysis of the Aedes aegypti eggshell
Source: BMC Dev Biol. 2014 Apr 5;14:15. doi: 10.1186/1471-213X-14-15 (PMC4234484; doi:10.1186/1471-213X-14-15)
Supplement: Additional file 4 — Expression profiles of selected Aedes aegypti eggshell- related genes. RNAseq data extracted from Akbari et al., 2013 show transcript abundance (FPKM) at developmental stages and dissected tissues, including ovaries. Microarray data (Dissanayake et al., 2010) are presented as an inset when available, and represent the abundance of specific mRNAs in samples derived from non-blood fed whole females (NBF) and those at 3, 12, 24, 48, 72, and 96 hours following a blood meal (BF). A sample from adult males (M) also is included. [file 1471-213X-14-15-S4.pptx]

## Slide 1
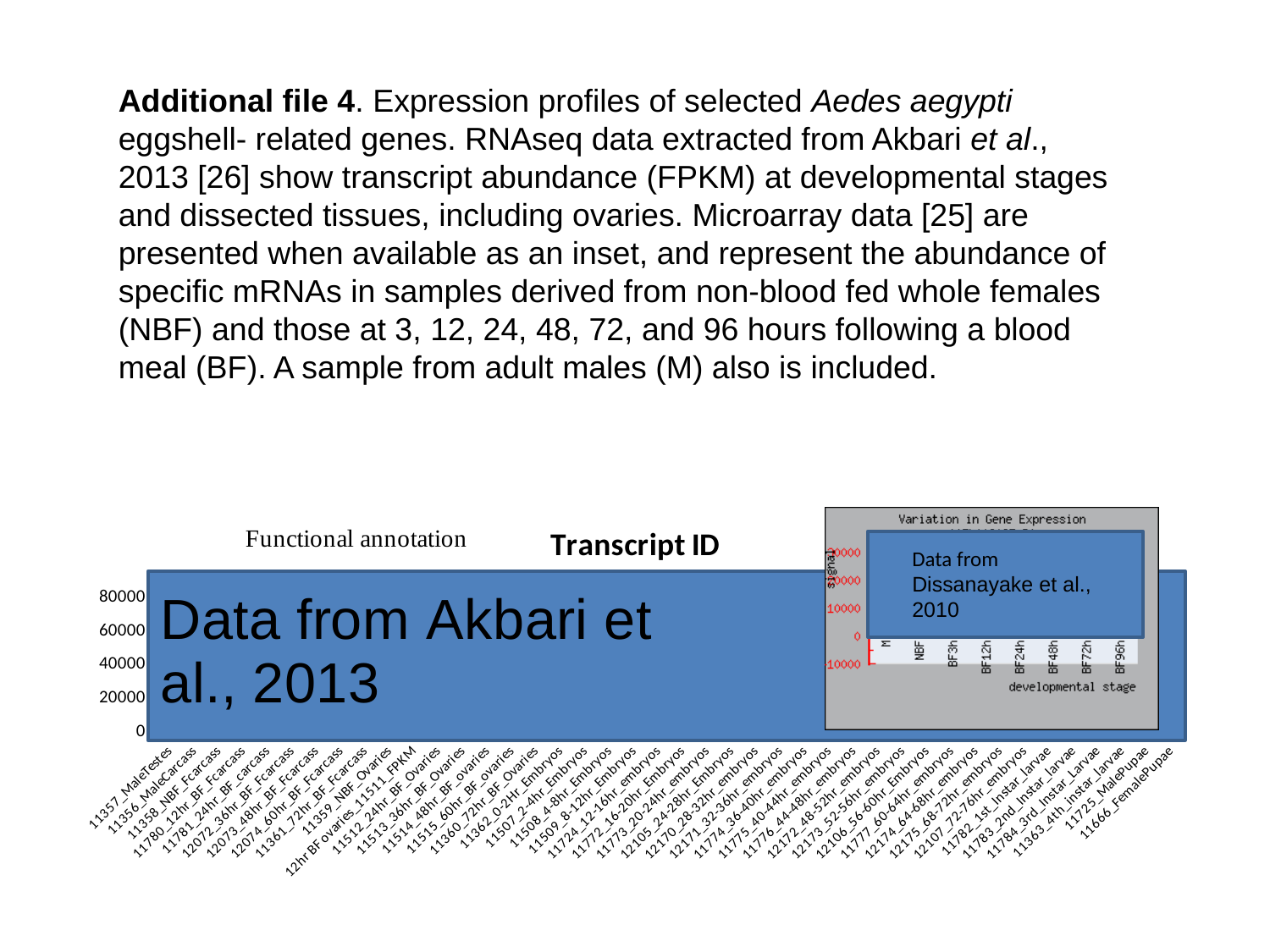

Additional file 4. Expression profiles of selected Aedes aegypti eggshell- related genes. RNAseq data extracted from Akbari et al., 2013 [26] show transcript abundance (FPKM) at developmental stages and dissected tissues, including ovaries. Microarray data [25] are presented when available as an inset, and represent the abundance of specific mRNAs in samples derived from non-blood fed whole females (NBF) and those at 3, 12, 24, 48, 72, and 96 hours following a blood meal (BF). A sample from adult males (M) also is included.
### Chart: Transcript ID
| Category | AAEL014561-RA |
|---|---|
| 11357_MaleTestes | 0.0 |
| 11356_MaleCarcass | 0.0 |
| 11358_NBF_Fcarcass | 0.0 |
| 11780_12hr_BF_Fcarcass | 22.6915 |
| 11781_24hr_BF_carcass | 207.775 |
| 12072_36hr_BF_Fcarcass | 63.4263 |
| 12073_48hr_BF_Fcarcass | 1.85602 |
| 12074_60hr_BF_Fcarcass | 4.53366 |
| 11361_72hr_BF_Fcarcass | 0.18744 |
| 11359_NBF_Ovaries | 0.0964118 |
| 12hr BF ovaries_11511_FPKM | 1543.0 |
| 11512_24hr_BF_Ovaries | 40375.3 |
| 11513_36hr_BF_Ovaries | 80140.5 |
| 11514_48hr_BF_ovaries | 5.01993 |
| 11515_60hr_BF_ovaries | 6.19372 |
| 11360_72hr_BF_Ovaries | 0.0 |
| 11362_0-2Hr_Embryos | 0.0 |
| 11507_2-4hr_Embryos | 0.149545 |
| 11508_4-8hr_Embryos | 0.244525 |
| 11509_8-12hr_Embryos | 0.0 |
| 11724_12-16hr_embryos | 102.054 |
| 11772_16-20hr_Embryos | 13.7396 |
| 11773_20-24hr_embryos | 17.3529 |
| 12105_24-28hr_Embryos | 0.10421 |
| 12170_28-32hr_embryos | 0.0 |
| 12171_32-36hr_embryos | 0.0 |
| 11774_36-40hr_embryos | 19.6167 |
| 11775_40-44hr_embryos | 58.6904 |
| 11776_44-48hr_embryos | 16.8916 |
| 12172_48-52hr_embryos | 2.26215 |
| 12173_52-56hr_embryos | 1.72683 |
| 12106_56-60hr_Embryos | 0.412164 |
| 11777_60-64hr_embryos | 17.921 |
| 12174_64-68hr_embryos | 0.0 |
| 12175_68-72hr_embryos | 0.108481 |
| 12107_72-76hr_embryos | 0.0 |
| 11782_1st_Instar_larvae | 0.728095 |
| 11783_2nd_Instar_larvae | 0.382103 |
| 11784_3rd_Instar_Larvae | 0.0500094 |
| 11363_4th_instar_larvae | 0.530921 |
| 11725_MalePupae | 0.108481 |
| 11666_FemalePupae | 1251.7 |
Data from Dissanayake et al., 2010

## Slide 2
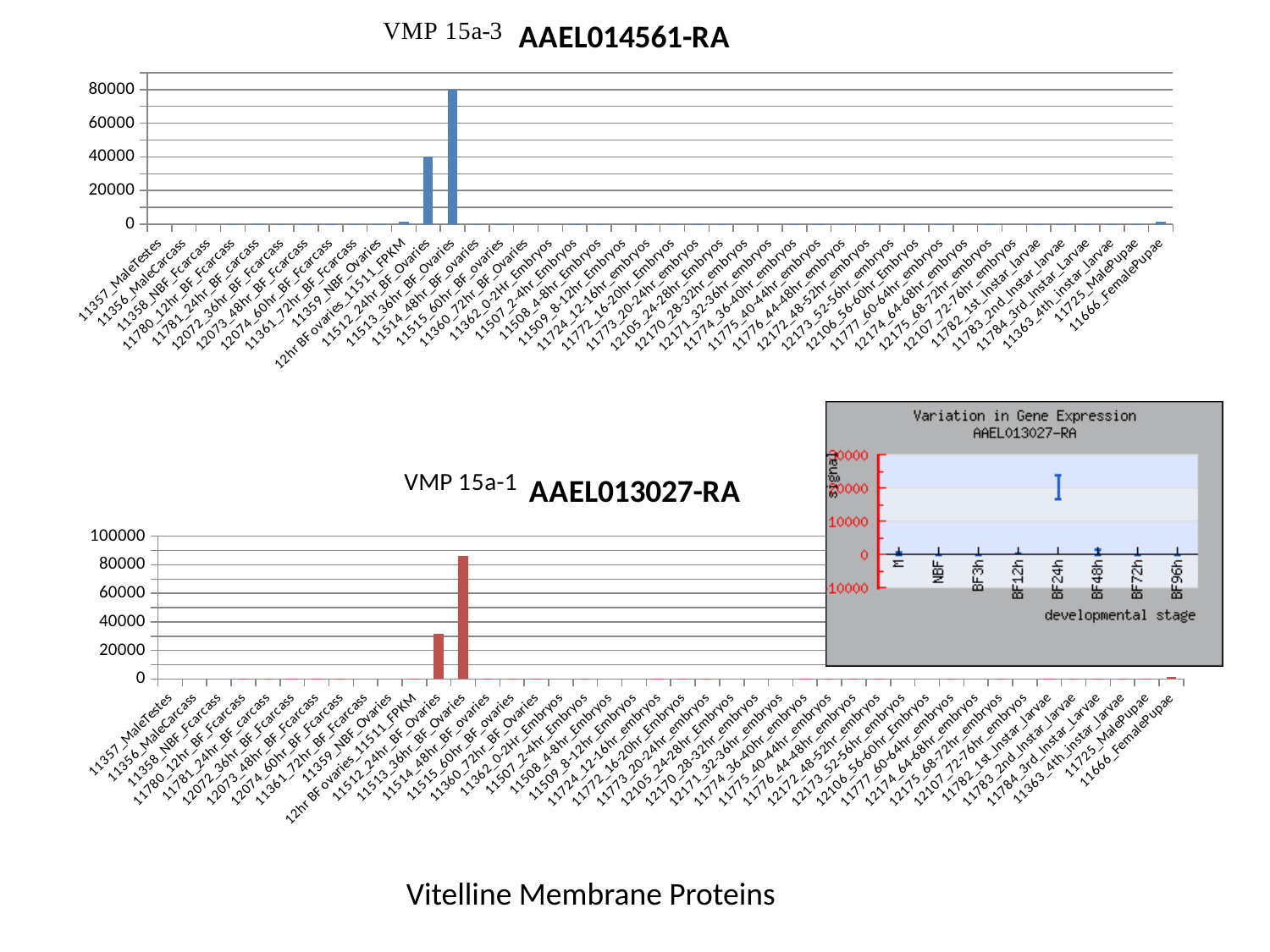

### Chart:
| Category | AAEL014561-RA |
|---|---|
| 11357_MaleTestes | 0.0 |
| 11356_MaleCarcass | 0.0 |
| 11358_NBF_Fcarcass | 0.0 |
| 11780_12hr_BF_Fcarcass | 22.6915 |
| 11781_24hr_BF_carcass | 207.775 |
| 12072_36hr_BF_Fcarcass | 63.4263 |
| 12073_48hr_BF_Fcarcass | 1.85602 |
| 12074_60hr_BF_Fcarcass | 4.53366 |
| 11361_72hr_BF_Fcarcass | 0.18744 |
| 11359_NBF_Ovaries | 0.0964118 |
| 12hr BF ovaries_11511_FPKM | 1543.0 |
| 11512_24hr_BF_Ovaries | 40375.3 |
| 11513_36hr_BF_Ovaries | 80140.5 |
| 11514_48hr_BF_ovaries | 5.01993 |
| 11515_60hr_BF_ovaries | 6.19372 |
| 11360_72hr_BF_Ovaries | 0.0 |
| 11362_0-2Hr_Embryos | 0.0 |
| 11507_2-4hr_Embryos | 0.149545 |
| 11508_4-8hr_Embryos | 0.244525 |
| 11509_8-12hr_Embryos | 0.0 |
| 11724_12-16hr_embryos | 102.054 |
| 11772_16-20hr_Embryos | 13.7396 |
| 11773_20-24hr_embryos | 17.3529 |
| 12105_24-28hr_Embryos | 0.10421 |
| 12170_28-32hr_embryos | 0.0 |
| 12171_32-36hr_embryos | 0.0 |
| 11774_36-40hr_embryos | 19.6167 |
| 11775_40-44hr_embryos | 58.6904 |
| 11776_44-48hr_embryos | 16.8916 |
| 12172_48-52hr_embryos | 2.26215 |
| 12173_52-56hr_embryos | 1.72683 |
| 12106_56-60hr_Embryos | 0.412164 |
| 11777_60-64hr_embryos | 17.921 |
| 12174_64-68hr_embryos | 0.0 |
| 12175_68-72hr_embryos | 0.108481 |
| 12107_72-76hr_embryos | 0.0 |
| 11782_1st_Instar_larvae | 0.728095 |
| 11783_2nd_Instar_larvae | 0.382103 |
| 11784_3rd_Instar_Larvae | 0.0500094 |
| 11363_4th_instar_larvae | 0.530921 |
| 11725_MalePupae | 0.108481 |
| 11666_FemalePupae | 1251.7 |
### Chart:
| Category | AAEL013027-RA |
|---|---|
| 11357_MaleTestes | 0.0 |
| 11356_MaleCarcass | 0.0 |
| 11358_NBF_Fcarcass | 0.0 |
| 11780_12hr_BF_Fcarcass | 26.8793 |
| 11781_24hr_BF_carcass | 153.397 |
| 12072_36hr_BF_Fcarcass | 73.1537 |
| 12073_48hr_BF_Fcarcass | 3.73318 |
| 12074_60hr_BF_Fcarcass | 4.69328 |
| 11361_72hr_BF_Fcarcass | 0.0 |
| 11359_NBF_Ovaries | 0.0 |
| 12hr BF ovaries_11511_FPKM | 63.0235 |
| 11512_24hr_BF_Ovaries | 31912.7 |
| 11513_36hr_BF_Ovaries | 86182.2 |
| 11514_48hr_BF_ovaries | 58.0489 |
| 11515_60hr_BF_ovaries | 44.9651 |
| 11360_72hr_BF_Ovaries | 0.491996 |
| 11362_0-2Hr_Embryos | 0.0 |
| 11507_2-4hr_Embryos | 0.14195 |
| 11508_4-8hr_Embryos | 0.0 |
| 11509_8-12hr_Embryos | 0.0 |
| 11724_12-16hr_embryos | 96.448 |
| 11772_16-20hr_Embryos | 16.1096 |
| 11773_20-24hr_embryos | 18.0169 |
| 12105_24-28hr_Embryos | 0.0 |
| 12170_28-32hr_embryos | 0.0 |
| 12171_32-36hr_embryos | 0.0 |
| 11774_36-40hr_embryos | 24.9959 |
| 11775_40-44hr_embryos | 74.6495 |
| 11776_44-48hr_embryos | 19.8837 |
| 12172_48-52hr_embryos | 0.104733 |
| 12173_52-56hr_embryos | 0.0 |
| 12106_56-60hr_Embryos | 0.0 |
| 11777_60-64hr_embryos | 19.6455 |
| 12174_64-68hr_embryos | 0.0 |
| 12175_68-72hr_embryos | 0.102972 |
| 12107_72-76hr_embryos | 0.0 |
| 11782_1st_Instar_larvae | 0.845776 |
| 11783_2nd_Instar_larvae | 0.339092 |
| 11784_3rd_Instar_Larvae | 0.10549 |
| 11363_4th_instar_larvae | 0.167986 |
| 11725_MalePupae | 0.102972 |
| 11666_FemalePupae | 1235.44 |Vitelline Membrane Proteins

## Slide 3
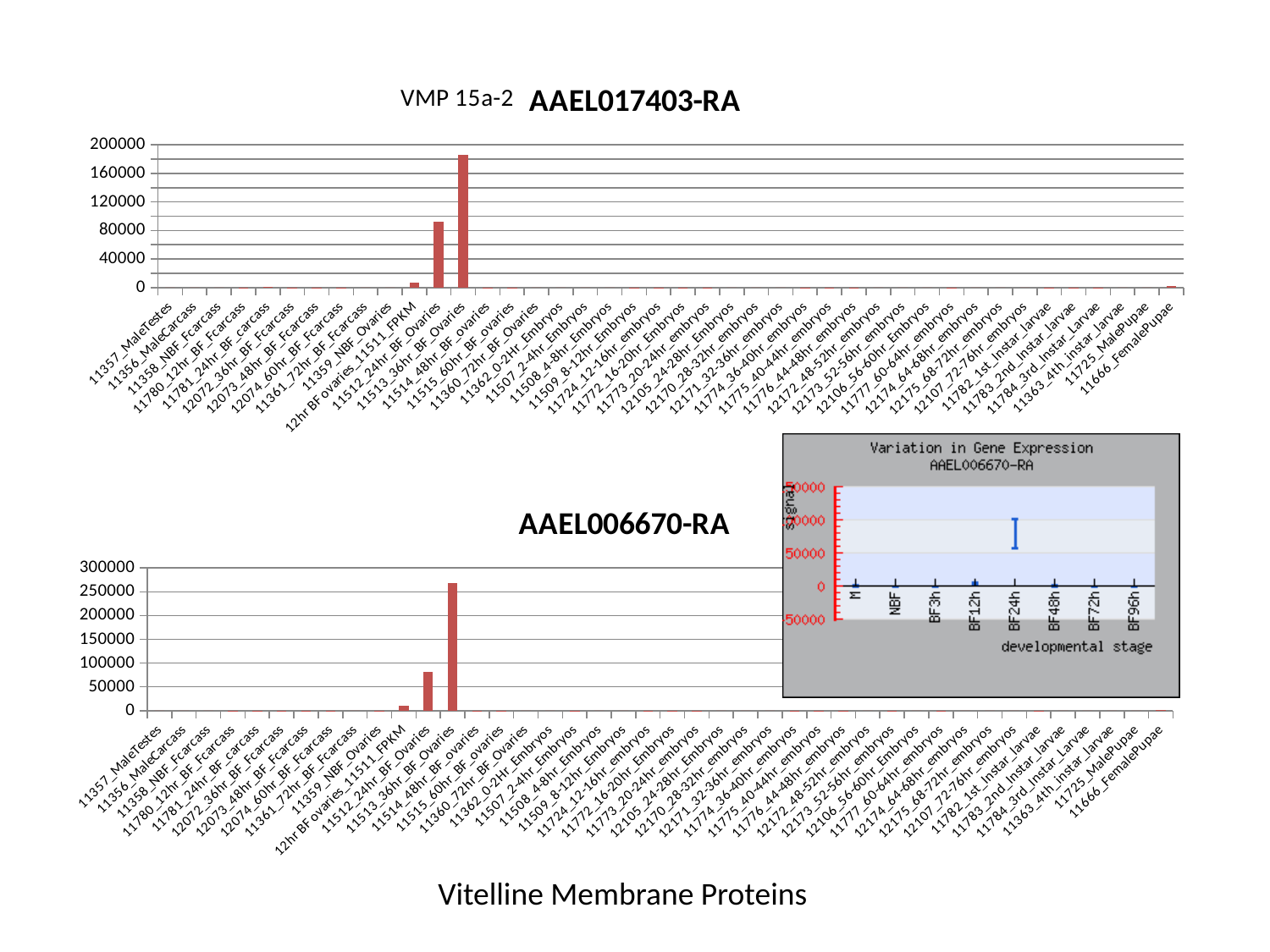

### Chart:
| Category | AAEL017403-RA |
|---|---|
| 11357_MaleTestes | 0.0 |
| 11356_MaleCarcass | 0.0 |
| 11358_NBF_Fcarcass | 0.0 |
| 11780_12hr_BF_Fcarcass | 53.9734 |
| 11781_24hr_BF_carcass | 828.355 |
| 12072_36hr_BF_Fcarcass | 191.601 |
| 12073_48hr_BF_Fcarcass | 20.8898 |
| 12074_60hr_BF_Fcarcass | 10.0932 |
| 11361_72hr_BF_Fcarcass | 0.0 |
| 11359_NBF_Ovaries | 0.0 |
| 12hr BF ovaries_11511_FPKM | 6673.27 |
| 11512_24hr_BF_Ovaries | 92097.5 |
| 11513_36hr_BF_Ovaries | 186450.0 |
| 11514_48hr_BF_ovaries | 188.777 |
| 11515_60hr_BF_ovaries | 81.5295 |
| 11360_72hr_BF_Ovaries | 0.0 |
| 11362_0-2Hr_Embryos | 0.0 |
| 11507_2-4hr_Embryos | 0.0 |
| 11508_4-8hr_Embryos | 0.0 |
| 11509_8-12hr_Embryos | 1.2323 |
| 11724_12-16hr_embryos | 212.803 |
| 11772_16-20hr_Embryos | 38.0129 |
| 11773_20-24hr_embryos | 43.2993 |
| 12105_24-28hr_Embryos | 0.0 |
| 12170_28-32hr_embryos | 0.0 |
| 12171_32-36hr_embryos | 0.0 |
| 11774_36-40hr_embryos | 55.13 |
| 11775_40-44hr_embryos | 160.141 |
| 11776_44-48hr_embryos | 47.7413 |
| 12172_48-52hr_embryos | 0.0 |
| 12173_52-56hr_embryos | 0.0 |
| 12106_56-60hr_Embryos | 0.0 |
| 11777_60-64hr_embryos | 37.4996 |
| 12174_64-68hr_embryos | 0.0 |
| 12175_68-72hr_embryos | 0.0 |
| 12107_72-76hr_embryos | 0.0 |
| 11782_1st_Instar_larvae | 5.2008 |
| 11783_2nd_Instar_larvae | 0.97306 |
| 11784_3rd_Instar_Larvae | 1.36221 |
| 11363_4th_instar_larvae | 0.0 |
| 11725_MalePupae | 0.0 |
| 11666_FemalePupae | 1748.71 |
### Chart:
| Category | AAEL006670-RA |
|---|---|
| 11357_MaleTestes | 0.0 |
| 11356_MaleCarcass | 0.0 |
| 11358_NBF_Fcarcass | 0.0 |
| 11780_12hr_BF_Fcarcass | 20.0272 |
| 11781_24hr_BF_carcass | 604.106 |
| 12072_36hr_BF_Fcarcass | 104.961 |
| 12073_48hr_BF_Fcarcass | 4.53798 |
| 12074_60hr_BF_Fcarcass | 14.5876 |
| 11361_72hr_BF_Fcarcass | 0.0 |
| 11359_NBF_Ovaries | 1.65278 |
| 12hr BF ovaries_11511_FPKM | 10192.1 |
| 11512_24hr_BF_Ovaries | 81032.4 |
| 11513_36hr_BF_Ovaries | 268202.0 |
| 11514_48hr_BF_ovaries | 5.74027 |
| 11515_60hr_BF_ovaries | 10.8014 |
| 11360_72hr_BF_Ovaries | 0.0 |
| 11362_0-2Hr_Embryos | 0.0 |
| 11507_2-4hr_Embryos | 1.15364 |
| 11508_4-8hr_Embryos | 0.0 |
| 11509_8-12hr_Embryos | 0.0 |
| 11724_12-16hr_embryos | 175.886 |
| 11772_16-20hr_Embryos | 25.047 |
| 11773_20-24hr_embryos | 51.661 |
| 12105_24-28hr_Embryos | 0.0 |
| 12170_28-32hr_embryos | 0.0 |
| 12171_32-36hr_embryos | 0.0 |
| 11774_36-40hr_embryos | 39.0036 |
| 11775_40-44hr_embryos | 125.56 |
| 11776_44-48hr_embryos | 30.0508 |
| 12172_48-52hr_embryos | 0.0 |
| 12173_52-56hr_embryos | 0.427392 |
| 12106_56-60hr_Embryos | 0.0 |
| 11777_60-64hr_embryos | 30.6254 |
| 12174_64-68hr_embryos | 0.0 |
| 12175_68-72hr_embryos | 0.0 |
| 12107_72-76hr_embryos | 0.0 |
| 11782_1st_Instar_larvae | 0.979153 |
| 11783_2nd_Instar_larvae | 0.0 |
| 11784_3rd_Instar_Larvae | 0.0 |
| 11363_4th_instar_larvae | 0.0 |
| 11725_MalePupae | 0.0 |
| 11666_FemalePupae | 1780.17 |Vitelline Membrane Proteins

## Slide 4
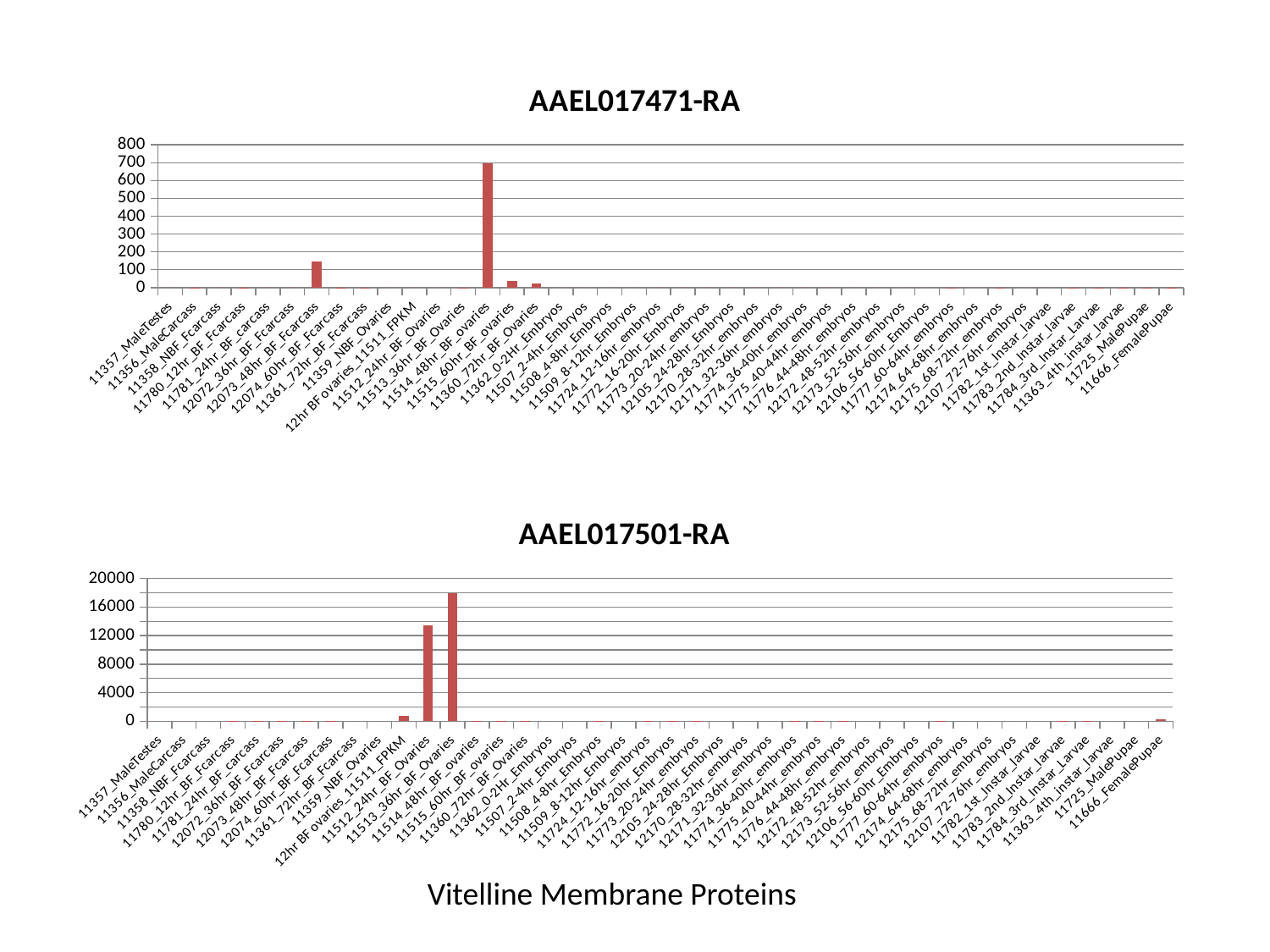

### Chart:
| Category | AAEL017471-RA |
|---|---|
| 11357_MaleTestes | 0.0 |
| 11356_MaleCarcass | 0.18936 |
| 11358_NBF_Fcarcass | 0.0 |
| 11780_12hr_BF_Fcarcass | 0.64007 |
| 11781_24hr_BF_carcass | 0.0 |
| 12072_36hr_BF_Fcarcass | 0.0 |
| 12073_48hr_BF_Fcarcass | 148.533 |
| 12074_60hr_BF_Fcarcass | 0.404399 |
| 11361_72hr_BF_Fcarcass | 0.797573 |
| 11359_NBF_Ovaries | 0.0 |
| 12hr BF ovaries_11511_FPKM | 0.0 |
| 11512_24hr_BF_Ovaries | 0.0 |
| 11513_36hr_BF_Ovaries | 0.114834 |
| 11514_48hr_BF_ovaries | 695.789 |
| 11515_60hr_BF_ovaries | 38.9083 |
| 11360_72hr_BF_Ovaries | 20.5859 |
| 11362_0-2Hr_Embryos | 0.0 |
| 11507_2-4hr_Embryos | 0.0 |
| 11508_4-8hr_Embryos | 0.0 |
| 11509_8-12hr_Embryos | 0.0 |
| 11724_12-16hr_embryos | 0.0 |
| 11772_16-20hr_Embryos | 0.0 |
| 11773_20-24hr_embryos | 0.0 |
| 12105_24-28hr_Embryos | 0.0 |
| 12170_28-32hr_embryos | 0.0 |
| 12171_32-36hr_embryos | 0.0 |
| 11774_36-40hr_embryos | 0.0 |
| 11775_40-44hr_embryos | 0.0 |
| 11776_44-48hr_embryos | 0.0 |
| 12172_48-52hr_embryos | 0.0 |
| 12173_52-56hr_embryos | 0.0 |
| 12106_56-60hr_Embryos | 0.0 |
| 11777_60-64hr_embryos | 0.527339 |
| 12174_64-68hr_embryos | 0.0 |
| 12175_68-72hr_embryos | 0.102577 |
| 12107_72-76hr_embryos | 0.0 |
| 11782_1st_Instar_larvae | 0.0 |
| 11783_2nd_Instar_larvae | 0.11257 |
| 11784_3rd_Instar_Larvae | 0.10506 |
| 11363_4th_instar_larvae | 1.04589 |
| 11725_MalePupae | 0.102577 |
| 11666_FemalePupae | 0.599539 |
### Chart:
| Category | AAEL017501-RA |
|---|---|
| 11357_MaleTestes | 0.0 |
| 11356_MaleCarcass | 0.0 |
| 11358_NBF_Fcarcass | 0.0 |
| 11780_12hr_BF_Fcarcass | 2.10479 |
| 11781_24hr_BF_carcass | 73.7291 |
| 12072_36hr_BF_Fcarcass | 16.2817 |
| 12073_48hr_BF_Fcarcass | 1.83335 |
| 12074_60hr_BF_Fcarcass | 1.77202 |
| 11361_72hr_BF_Fcarcass | 0.0 |
| 11359_NBF_Ovaries | 0.0 |
| 12hr BF ovaries_11511_FPKM | 772.125 |
| 11512_24hr_BF_Ovaries | 13379.4 |
| 11513_36hr_BF_Ovaries | 17958.2 |
| 11514_48hr_BF_ovaries | 3.49419 |
| 11515_60hr_BF_ovaries | 1.57957 |
| 11360_72hr_BF_Ovaries | 0.537219 |
| 11362_0-2Hr_Embryos | 0.0 |
| 11507_2-4hr_Embryos | 0.0 |
| 11508_4-8hr_Embryos | 0.427684 |
| 11509_8-12hr_Embryos | 0.0 |
| 11724_12-16hr_embryos | 14.3843 |
| 11772_16-20hr_Embryos | 3.69733 |
| 11773_20-24hr_embryos | 3.72752 |
| 12105_24-28hr_Embryos | 0.0 |
| 12170_28-32hr_embryos | 0.0 |
| 12171_32-36hr_embryos | 0.0 |
| 11774_36-40hr_embryos | 5.89541 |
| 11775_40-44hr_embryos | 20.8947 |
| 11776_44-48hr_embryos | 5.69478 |
| 12172_48-52hr_embryos | 0.0 |
| 12173_52-56hr_embryos | 0.0 |
| 12106_56-60hr_Embryos | 0.0 |
| 11777_60-64hr_embryos | 2.31212 |
| 12174_64-68hr_embryos | 0.0 |
| 12175_68-72hr_embryos | 0.0 |
| 12107_72-76hr_embryos | 0.0 |
| 11782_1st_Instar_larvae | 0.0 |
| 11783_2nd_Instar_larvae | 1.11018 |
| 11784_3rd_Instar_Larvae | 0.345371 |
| 11363_4th_instar_larvae | 0.0 |
| 11725_MalePupae | 0.0 |
| 11666_FemalePupae | 276.011 |Vitelline Membrane Proteins

## Slide 5
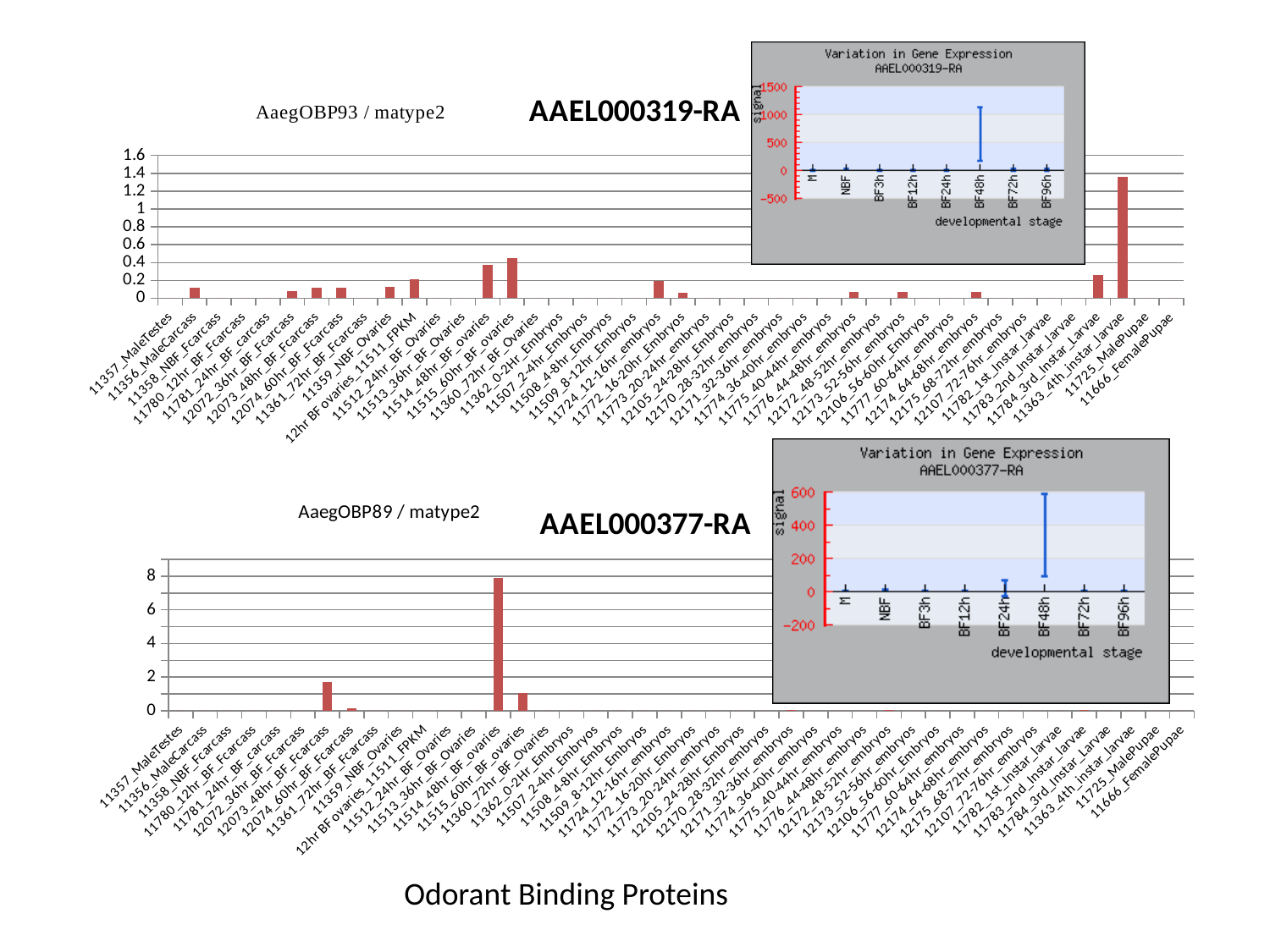

### Chart:
| Category | AAEL000319-RA |
|---|---|
| 11357_MaleTestes | 0.0 |
| 11356_MaleCarcass | 0.118865 |
| 11358_NBF_Fcarcass | 0.0 |
| 11780_12hr_BF_Fcarcass | 0.0 |
| 11781_24hr_BF_carcass | 0.0 |
| 12072_36hr_BF_Fcarcass | 0.079667 |
| 12073_48hr_BF_Fcarcass | 0.116692 |
| 12074_60hr_BF_Fcarcass | 0.112859 |
| 11361_72hr_BF_Fcarcass | 0.0 |
| 11359_NBF_Ovaries | 0.127127 |
| 12hr BF ovaries_11511_FPKM | 0.209741 |
| 11512_24hr_BF_Ovaries | 0.0 |
| 11513_36hr_BF_Ovaries | 0.0 |
| 11514_48hr_BF_ovaries | 0.371129 |
| 11515_60hr_BF_ovaries | 0.452138 |
| 11360_72hr_BF_Ovaries | 0.0 |
| 11362_0-2Hr_Embryos | 0.0 |
| 11507_2-4hr_Embryos | 0.0 |
| 11508_4-8hr_Embryos | 0.0 |
| 11509_8-12hr_Embryos | 0.0 |
| 11724_12-16hr_embryos | 0.196066 |
| 11772_16-20hr_Embryos | 0.0641413 |
| 11773_20-24hr_embryos | 0.0 |
| 12105_24-28hr_Embryos | 0.0 |
| 12170_28-32hr_embryos | 0.0 |
| 12171_32-36hr_embryos | 0.0 |
| 11774_36-40hr_embryos | 0.0 |
| 11775_40-44hr_embryos | 0.0 |
| 11776_44-48hr_embryos | 0.0679202 |
| 12172_48-52hr_embryos | 0.0 |
| 12173_52-56hr_embryos | 0.0659412 |
| 12106_56-60hr_Embryos | 0.0 |
| 11777_60-64hr_embryos | 0.0 |
| 12174_64-68hr_embryos | 0.0686942 |
| 12175_68-72hr_embryos | 0.0 |
| 12107_72-76hr_embryos | 0.0 |
| 11782_1st_Instar_larvae | 0.0 |
| 11783_2nd_Instar_larvae | 0.0 |
| 11784_3rd_Instar_Larvae | 0.263793 |
| 11363_4th_instar_larvae | 1.36512 |
| 11725_MalePupae | 0.0 |
| 11666_FemalePupae | 0.0 |
### Chart:
| Category | AAEL000377-RA |
|---|---|
| 11357_MaleTestes | 0.0 |
| 11356_MaleCarcass | 0.0 |
| 11358_NBF_Fcarcass | 0.0 |
| 11780_12hr_BF_Fcarcass | 0.0 |
| 11781_24hr_BF_carcass | 0.0 |
| 12072_36hr_BF_Fcarcass | 0.0 |
| 12073_48hr_BF_Fcarcass | 1.68104 |
| 12074_60hr_BF_Fcarcass | 0.128359 |
| 11361_72hr_BF_Fcarcass | 0.0 |
| 11359_NBF_Ovaries | 0.0 |
| 12hr BF ovaries_11511_FPKM | 0.0 |
| 11512_24hr_BF_Ovaries | 0.0 |
| 11513_36hr_BF_Ovaries | 0.0 |
| 11514_48hr_BF_ovaries | 7.87947 |
| 11515_60hr_BF_ovaries | 1.0284 |
| 11360_72hr_BF_Ovaries | 0.0 |
| 11362_0-2Hr_Embryos | 0.0 |
| 11507_2-4hr_Embryos | 0.0 |
| 11508_4-8hr_Embryos | 0.0 |
| 11509_8-12hr_Embryos | 0.0 |
| 11724_12-16hr_embryos | 0.0 |
| 11772_16-20hr_Embryos | 0.0 |
| 11773_20-24hr_embryos | 0.0 |
| 12105_24-28hr_Embryos | 0.0 |
| 12170_28-32hr_embryos | 0.0 |
| 12171_32-36hr_embryos | 0.053829 |
| 11774_36-40hr_embryos | 0.0 |
| 11775_40-44hr_embryos | 0.0 |
| 11776_44-48hr_embryos | 0.0 |
| 12172_48-52hr_embryos | 0.0496554 |
| 12173_52-56hr_embryos | 0.0 |
| 12106_56-60hr_Embryos | 0.0 |
| 11777_60-64hr_embryos | 0.0 |
| 12174_64-68hr_embryos | 0.0 |
| 12175_68-72hr_embryos | 0.0 |
| 12107_72-76hr_embryos | 0.0 |
| 11782_1st_Instar_larvae | 0.0 |
| 11783_2nd_Instar_larvae | 0.0535764 |
| 11784_3rd_Instar_Larvae | 0.0 |
| 11363_4th_instar_larvae | 0.0 |
| 11725_MalePupae | 0.0 |
| 11666_FemalePupae | 0.0 |Odorant Binding Proteins

## Slide 6
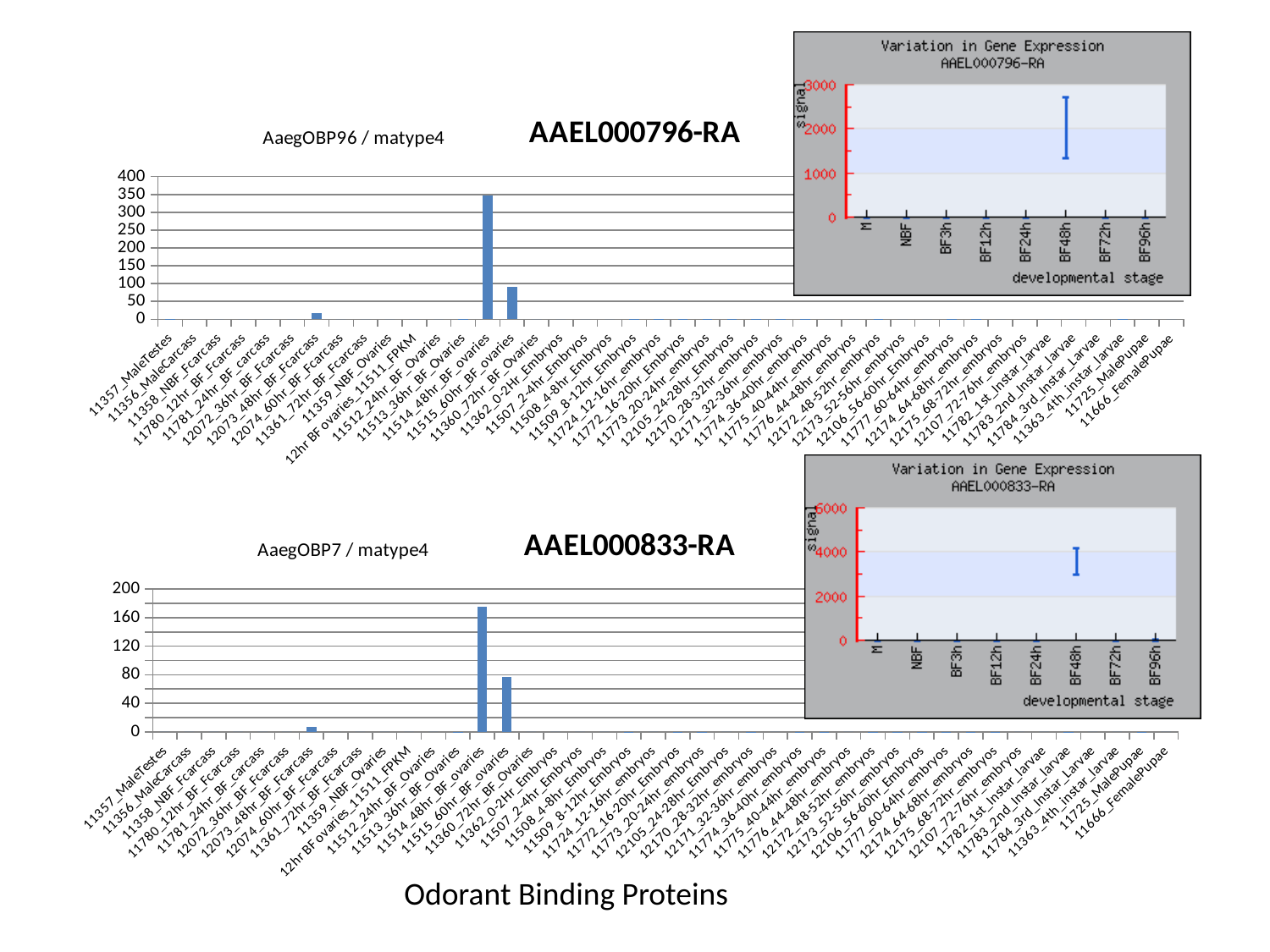

### Chart:
| Category | AAEL000796-RA |
|---|---|
| 11357_MaleTestes | 9.61447e-07 |
| 11356_MaleCarcass | 0.0 |
| 11358_NBF_Fcarcass | 0.0 |
| 11780_12hr_BF_Fcarcass | 0.0 |
| 11781_24hr_BF_carcass | 0.0 |
| 12072_36hr_BF_Fcarcass | 0.0 |
| 12073_48hr_BF_Fcarcass | 17.6506 |
| 12074_60hr_BF_Fcarcass | 0.0 |
| 11361_72hr_BF_Fcarcass | 0.0 |
| 11359_NBF_Ovaries | 0.0 |
| 12hr BF ovaries_11511_FPKM | 0.0 |
| 11512_24hr_BF_Ovaries | 0.0 |
| 11513_36hr_BF_Ovaries | 0.22451 |
| 11514_48hr_BF_ovaries | 346.734 |
| 11515_60hr_BF_ovaries | 89.9893 |
| 11360_72hr_BF_Ovaries | 0.0 |
| 11362_0-2Hr_Embryos | 0.0 |
| 11507_2-4hr_Embryos | 0.0 |
| 11508_4-8hr_Embryos | 0.0 |
| 11509_8-12hr_Embryos | 0.0270444 |
| 11724_12-16hr_embryos | 6.75071e-07 |
| 11772_16-20hr_Embryos | 0.019401 |
| 11773_20-24hr_embryos | 3.51047e-07 |
| 12105_24-28hr_Embryos | 4.25872e-07 |
| 12170_28-32hr_embryos | 0.0861853 |
| 12171_32-36hr_embryos | 3.71757e-09 |
| 11774_36-40hr_embryos | 0.0974589 |
| 11775_40-44hr_embryos | 0.0 |
| 11776_44-48hr_embryos | 0.0 |
| 12172_48-52hr_embryos | 4.24e-07 |
| 12173_52-56hr_embryos | 0.0 |
| 12106_56-60hr_Embryos | 0.0 |
| 11777_60-64hr_embryos | 5.06382e-07 |
| 12174_64-68hr_embryos | 4.73068e-07 |
| 12175_68-72hr_embryos | 0.0 |
| 12107_72-76hr_embryos | 0.0 |
| 11782_1st_Instar_larvae | 0.0 |
| 11783_2nd_Instar_larvae | 0.0 |
| 11784_3rd_Instar_Larvae | 0.0 |
| 11363_4th_instar_larvae | 0.0317624 |
| 11725_MalePupae | 0.0 |
| 11666_FemalePupae | 0.0 |
### Chart:
| Category | AAEL000833-RA |
|---|---|
| 11357_MaleTestes | 0.0 |
| 11356_MaleCarcass | 0.0 |
| 11358_NBF_Fcarcass | 0.0 |
| 11780_12hr_BF_Fcarcass | 0.0 |
| 11781_24hr_BF_carcass | 0.0 |
| 12072_36hr_BF_Fcarcass | 0.0 |
| 12073_48hr_BF_Fcarcass | 6.84283 |
| 12074_60hr_BF_Fcarcass | 0.0 |
| 11361_72hr_BF_Fcarcass | 0.0 |
| 11359_NBF_Ovaries | 0.0 |
| 12hr BF ovaries_11511_FPKM | 0.0 |
| 11512_24hr_BF_Ovaries | 0.0 |
| 11513_36hr_BF_Ovaries | 0.0528375 |
| 11514_48hr_BF_ovaries | 174.879 |
| 11515_60hr_BF_ovaries | 76.941 |
| 11360_72hr_BF_Ovaries | 0.0 |
| 11362_0-2Hr_Embryos | 0.0 |
| 11507_2-4hr_Embryos | 0.0 |
| 11508_4-8hr_Embryos | 0.0 |
| 11509_8-12hr_Embryos | 0.327678 |
| 11724_12-16hr_embryos | 0.0 |
| 11772_16-20hr_Embryos | 0.0940274 |
| 11773_20-24hr_embryos | 0.0434477 |
| 12105_24-28hr_Embryos | 0.0 |
| 12170_28-32hr_embryos | 0.0464099 |
| 12171_32-36hr_embryos | 0.0 |
| 11774_36-40hr_embryos | 0.045811 |
| 11775_40-44hr_embryos | 0.0456652 |
| 11776_44-48hr_embryos | 0.0 |
| 12172_48-52hr_embryos | 0.0960108 |
| 12173_52-56hr_embryos | 0.193339 |
| 12106_56-60hr_Embryos | 0.134442 |
| 11777_60-64hr_embryos | 0.215599 |
| 12174_64-68hr_embryos | 0.0503528 |
| 12175_68-72hr_embryos | 0.0943604 |
| 12107_72-76hr_embryos | 0.0 |
| 11782_1st_Instar_larvae | 0.0 |
| 11783_2nd_Instar_larvae | 0.207183 |
| 11784_3rd_Instar_Larvae | 0.0 |
| 11363_4th_instar_larvae | 0.0 |
| 11725_MalePupae | 0.0943604 |
| 11666_FemalePupae | 0.0 |Odorant Binding Proteins

## Slide 7
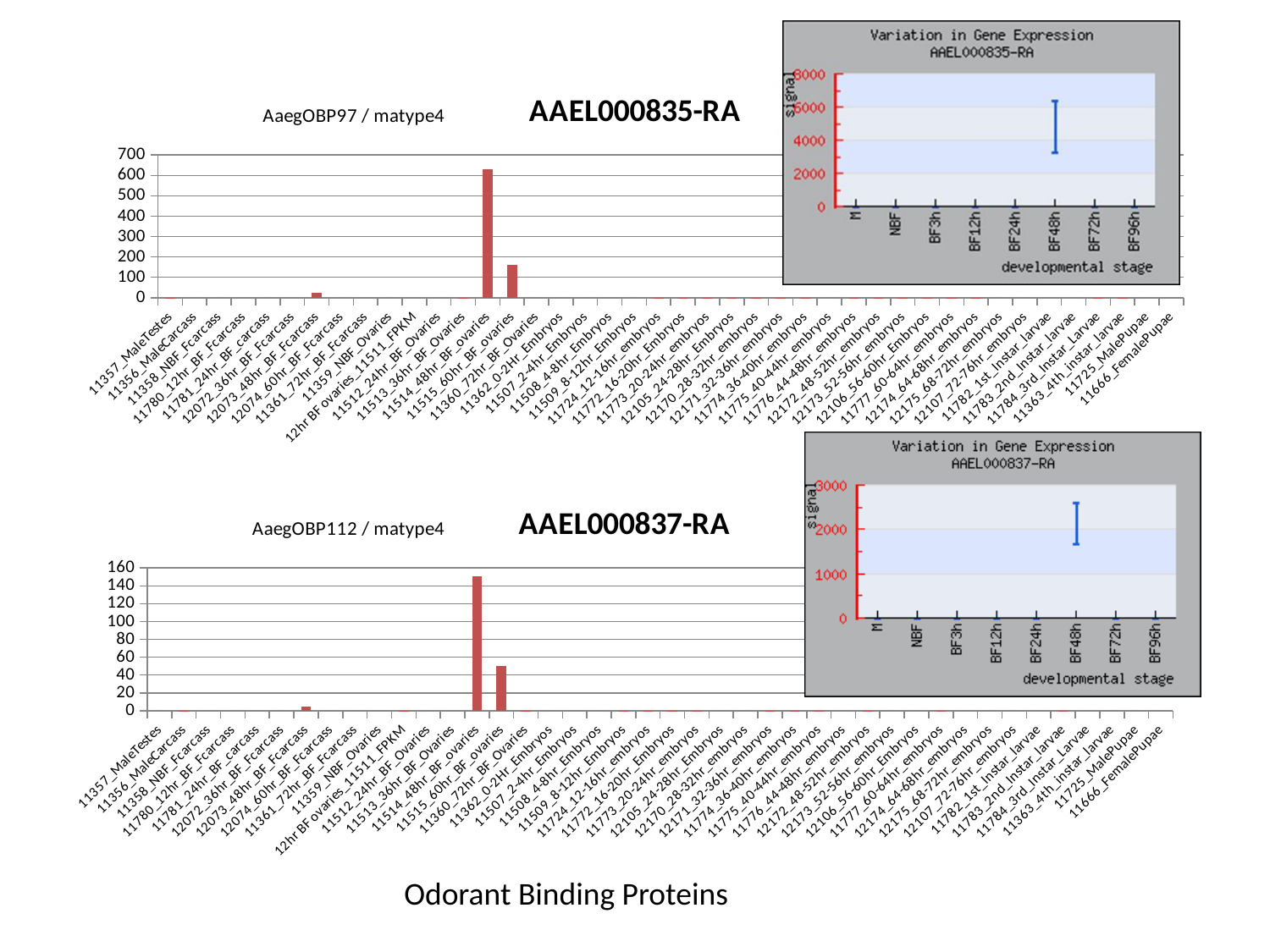

### Chart:
| Category | AAEL000835-RA |
|---|---|
| 11357_MaleTestes | 0.0784692 |
| 11356_MaleCarcass | 0.0 |
| 11358_NBF_Fcarcass | 0.0 |
| 11780_12hr_BF_Fcarcass | 0.0 |
| 11781_24hr_BF_carcass | 0.0 |
| 12072_36hr_BF_Fcarcass | 0.0 |
| 12073_48hr_BF_Fcarcass | 22.3357 |
| 12074_60hr_BF_Fcarcass | 0.0 |
| 11361_72hr_BF_Fcarcass | 0.0 |
| 11359_NBF_Ovaries | 0.0 |
| 12hr BF ovaries_11511_FPKM | 0.0 |
| 11512_24hr_BF_Ovaries | 0.0 |
| 11513_36hr_BF_Ovaries | 0.700975 |
| 11514_48hr_BF_ovaries | 628.666 |
| 11515_60hr_BF_ovaries | 160.388 |
| 11360_72hr_BF_Ovaries | 0.0 |
| 11362_0-2Hr_Embryos | 0.0 |
| 11507_2-4hr_Embryos | 0.0 |
| 11508_4-8hr_Embryos | 0.0 |
| 11509_8-12hr_Embryos | 0.0 |
| 11724_12-16hr_embryos | 0.449954 |
| 11772_16-20hr_Embryos | 0.0991306 |
| 11773_20-24hr_embryos | 0.241597 |
| 12105_24-28hr_Embryos | 0.156398 |
| 12170_28-32hr_embryos | 0.0160848 |
| 12171_32-36hr_embryos | 3.33517e-07 |
| 11774_36-40hr_embryos | 0.361952 |
| 11775_40-44hr_embryos | 0.0 |
| 11776_44-48hr_embryos | 0.0763449 |
| 12172_48-52hr_embryos | 0.165612 |
| 12173_52-56hr_embryos | 0.074119 |
| 12106_56-60hr_Embryos | 0.0687242 |
| 11777_60-64hr_embryos | 0.258304 |
| 12174_64-68hr_embryos | 0.241292 |
| 12175_68-72hr_embryos | 0.0 |
| 12107_72-76hr_embryos | 0.0 |
| 11782_1st_Instar_larvae | 0.0 |
| 11783_2nd_Instar_larvae | 0.0 |
| 11784_3rd_Instar_Larvae | 0.0741269 |
| 11363_4th_instar_larvae | 0.214367 |
| 11725_MalePupae | 0.0 |
| 11666_FemalePupae | 0.0 |
### Chart:
| Category | AAEL000837-RA |
|---|---|
| 11357_MaleTestes | 0.0 |
| 11356_MaleCarcass | 0.196457 |
| 11358_NBF_Fcarcass | 0.0 |
| 11780_12hr_BF_Fcarcass | 0.0 |
| 11781_24hr_BF_carcass | 0.0 |
| 12072_36hr_BF_Fcarcass | 0.0 |
| 12073_48hr_BF_Fcarcass | 4.53233 |
| 12074_60hr_BF_Fcarcass | 0.0 |
| 11361_72hr_BF_Fcarcass | 0.0 |
| 11359_NBF_Ovaries | 0.0 |
| 12hr BF ovaries_11511_FPKM | 0.0866612 |
| 11512_24hr_BF_Ovaries | 0.0 |
| 11513_36hr_BF_Ovaries | 0.0 |
| 11514_48hr_BF_ovaries | 150.288 |
| 11515_60hr_BF_ovaries | 50.5647 |
| 11360_72hr_BF_Ovaries | 0.0847158 |
| 11362_0-2Hr_Embryos | 0.0 |
| 11507_2-4hr_Embryos | 0.0 |
| 11508_4-8hr_Embryos | 0.0 |
| 11509_8-12hr_Embryos | 0.295544 |
| 11724_12-16hr_embryos | 0.0810108 |
| 11772_16-20hr_Embryos | 0.053004 |
| 11773_20-24hr_embryos | 0.0979673 |
| 12105_24-28hr_Embryos | 0.0 |
| 12170_28-32hr_embryos | 0.0 |
| 12171_32-36hr_embryos | 0.0586698 |
| 11774_36-40hr_embryos | 0.0516481 |
| 11775_40-44hr_embryos | 0.154451 |
| 11776_44-48hr_embryos | 0.0 |
| 12172_48-52hr_embryos | 0.0541198 |
| 12173_52-56hr_embryos | 0.0 |
| 12106_56-60hr_Embryos | 0.0 |
| 11777_60-64hr_embryos | 0.0607675 |
| 12174_64-68hr_embryos | 0.0 |
| 12175_68-72hr_embryos | 0.0 |
| 12107_72-76hr_embryos | 0.0 |
| 11782_1st_Instar_larvae | 0.0 |
| 11783_2nd_Instar_larvae | 0.0583945 |
| 11784_3rd_Instar_Larvae | 0.0 |
| 11363_4th_instar_larvae | 0.0 |
| 11725_MalePupae | 0.0 |
| 11666_FemalePupae | 0.0 |Odorant Binding Proteins

## Slide 8
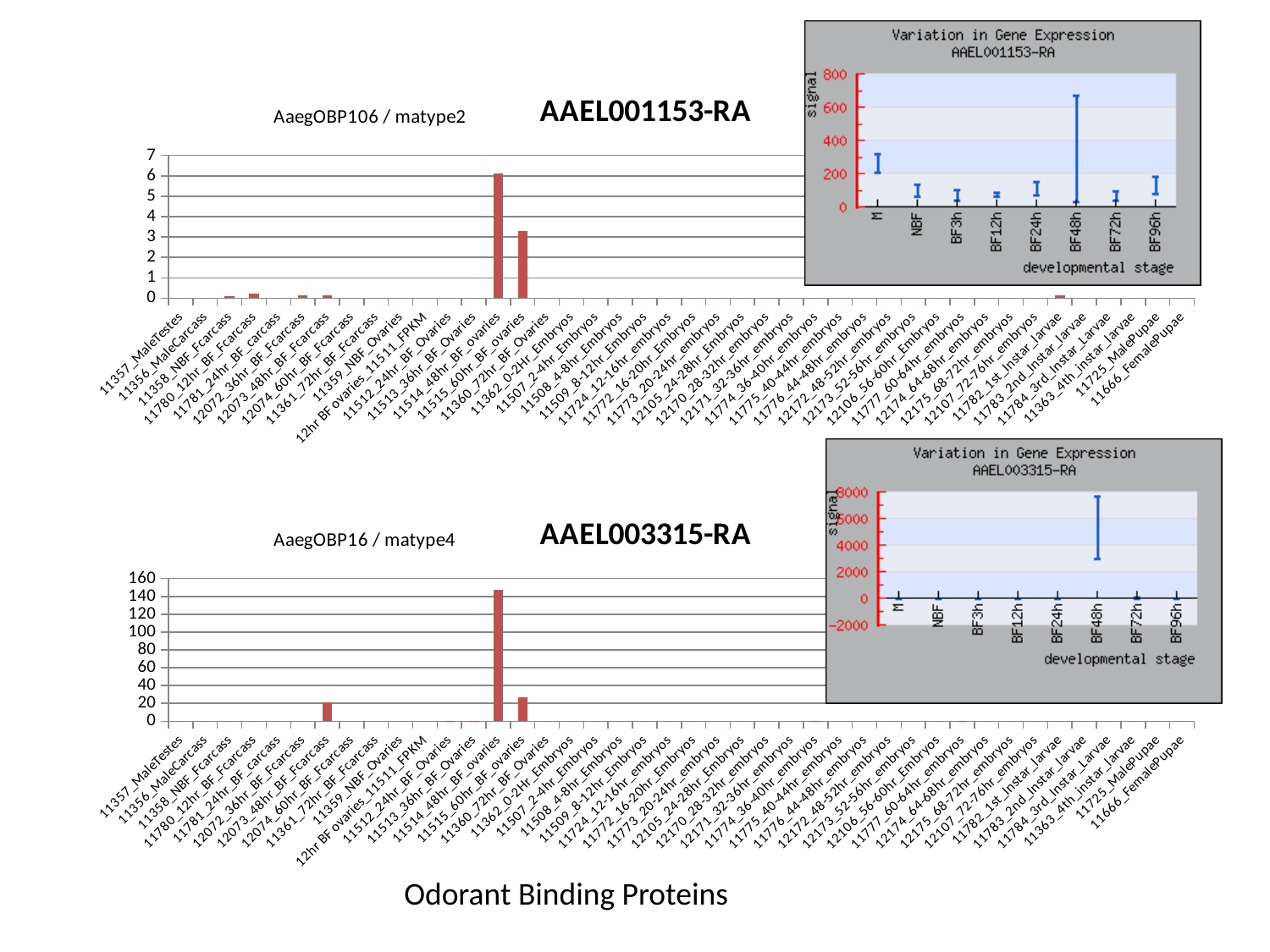

### Chart:
| Category | AAEL001153-RA |
|---|---|
| 11357_MaleTestes | 0.0 |
| 11356_MaleCarcass | 0.0 |
| 11358_NBF_Fcarcass | 0.093672 |
| 11780_12hr_BF_Fcarcass | 0.218006 |
| 11781_24hr_BF_carcass | 0.0 |
| 12072_36hr_BF_Fcarcass | 0.129723 |
| 12073_48hr_BF_Fcarcass | 0.142513 |
| 12074_60hr_BF_Fcarcass | 0.0 |
| 11361_72hr_BF_Fcarcass | 0.0 |
| 11359_NBF_Ovaries | 0.0 |
| 12hr BF ovaries_11511_FPKM | 0.0 |
| 11512_24hr_BF_Ovaries | 0.0 |
| 11513_36hr_BF_Ovaries | 0.0 |
| 11514_48hr_BF_ovaries | 6.10409 |
| 11515_60hr_BF_ovaries | 3.31301 |
| 11360_72hr_BF_Ovaries | 0.0 |
| 11362_0-2Hr_Embryos | 0.0 |
| 11507_2-4hr_Embryos | 0.0 |
| 11508_4-8hr_Embryos | 0.0 |
| 11509_8-12hr_Embryos | 0.0 |
| 11724_12-16hr_embryos | 0.0 |
| 11772_16-20hr_Embryos | 0.0 |
| 11773_20-24hr_embryos | 0.0 |
| 12105_24-28hr_Embryos | 0.0 |
| 12170_28-32hr_embryos | 0.0 |
| 12171_32-36hr_embryos | 0.0 |
| 11774_36-40hr_embryos | 0.0 |
| 11775_40-44hr_embryos | 0.0 |
| 11776_44-48hr_embryos | 0.0 |
| 12172_48-52hr_embryos | 0.0 |
| 12173_52-56hr_embryos | 0.0 |
| 12106_56-60hr_Embryos | 0.0 |
| 11777_60-64hr_embryos | 0.0 |
| 12174_64-68hr_embryos | 0.0 |
| 12175_68-72hr_embryos | 0.0 |
| 12107_72-76hr_embryos | 0.0 |
| 11782_1st_Instar_larvae | 0.122999 |
| 11783_2nd_Instar_larvae | 0.0 |
| 11784_3rd_Instar_Larvae | 0.0 |
| 11363_4th_instar_larvae | 0.0 |
| 11725_MalePupae | 0.0 |
| 11666_FemalePupae | 0.0 |
### Chart:
| Category | AAEL003315-RA |
|---|---|
| 11357_MaleTestes | 0.0 |
| 11356_MaleCarcass | 0.0 |
| 11358_NBF_Fcarcass | 0.0 |
| 11780_12hr_BF_Fcarcass | 0.0 |
| 11781_24hr_BF_carcass | 0.0 |
| 12072_36hr_BF_Fcarcass | 0.0 |
| 12073_48hr_BF_Fcarcass | 21.2071 |
| 12074_60hr_BF_Fcarcass | 0.0 |
| 11361_72hr_BF_Fcarcass | 0.0 |
| 11359_NBF_Ovaries | 0.0 |
| 12hr BF ovaries_11511_FPKM | 0.0 |
| 11512_24hr_BF_Ovaries | 0.0562286 |
| 11513_36hr_BF_Ovaries | 0.123296 |
| 11514_48hr_BF_ovaries | 147.343 |
| 11515_60hr_BF_ovaries | 27.0026 |
| 11360_72hr_BF_Ovaries | 0.0 |
| 11362_0-2Hr_Embryos | 0.0 |
| 11507_2-4hr_Embryos | 0.0 |
| 11508_4-8hr_Embryos | 0.0 |
| 11509_8-12hr_Embryos | 0.0 |
| 11724_12-16hr_embryos | 0.0 |
| 11772_16-20hr_Embryos | 0.0 |
| 11773_20-24hr_embryos | 0.0 |
| 12105_24-28hr_Embryos | 0.0 |
| 12170_28-32hr_embryos | 0.0 |
| 12171_32-36hr_embryos | 0.0 |
| 11774_36-40hr_embryos | 0.0534509 |
| 11775_40-44hr_embryos | 0.0 |
| 11776_44-48hr_embryos | 0.0 |
| 12172_48-52hr_embryos | 0.0 |
| 12173_52-56hr_embryos | 0.0 |
| 12106_56-60hr_Embryos | 0.0 |
| 11777_60-64hr_embryos | 0.125777 |
| 12174_64-68hr_embryos | 0.0 |
| 12175_68-72hr_embryos | 0.0 |
| 12107_72-76hr_embryos | 0.0 |
| 11782_1st_Instar_larvae | 0.0 |
| 11783_2nd_Instar_larvae | 0.0 |
| 11784_3rd_Instar_Larvae | 0.0 |
| 11363_4th_instar_larvae | 0.0 |
| 11725_MalePupae | 0.0 |
| 11666_FemalePupae | 0.0 |Odorant Binding Proteins

## Slide 9
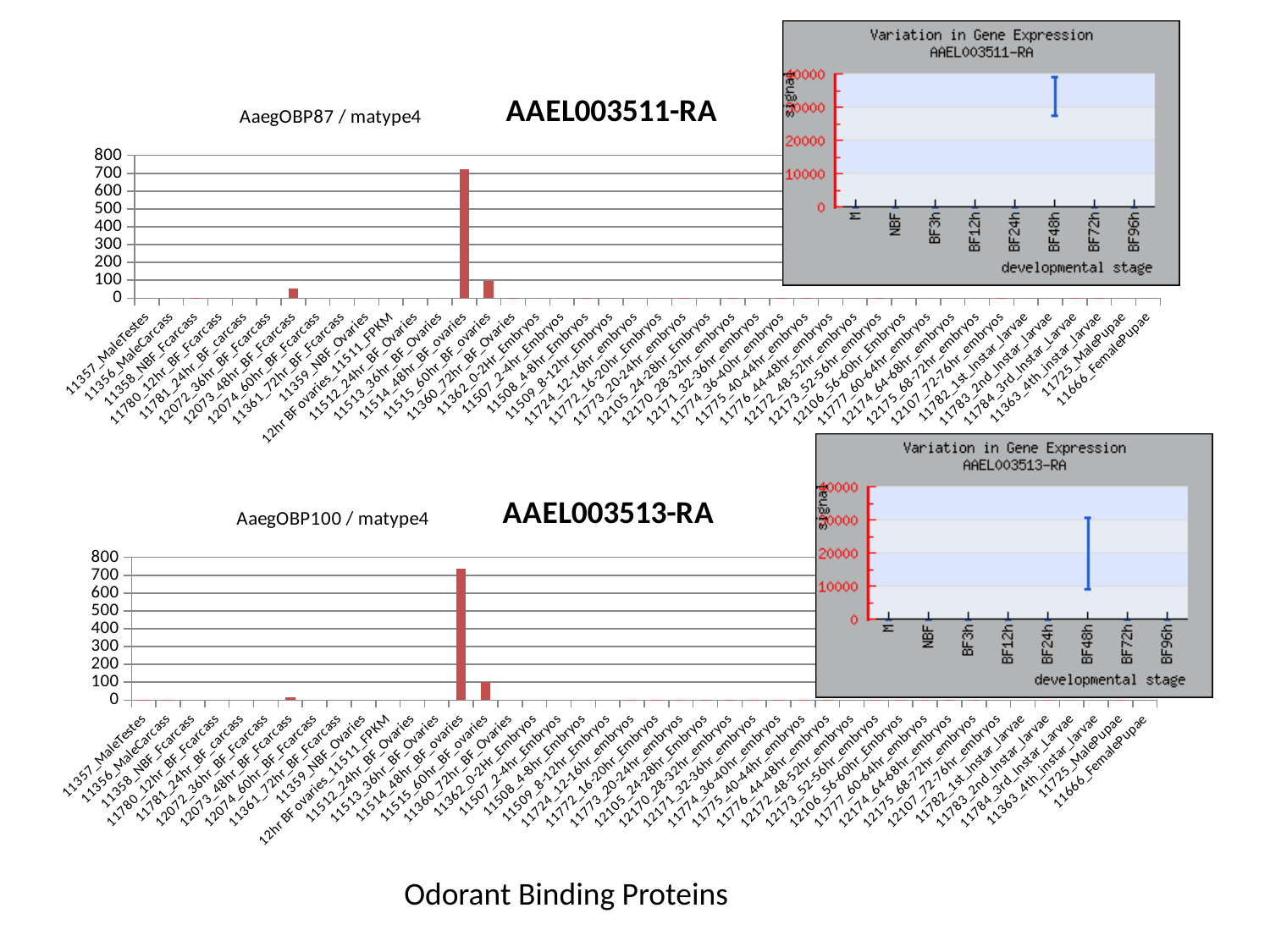

### Chart:
| Category | AAEL003511-RA |
|---|---|
| 11357_MaleTestes | 0.0 |
| 11356_MaleCarcass | 0.0 |
| 11358_NBF_Fcarcass | 0.106222 |
| 11780_12hr_BF_Fcarcass | 0.0 |
| 11781_24hr_BF_carcass | 0.0 |
| 12072_36hr_BF_Fcarcass | 0.0 |
| 12073_48hr_BF_Fcarcass | 53.4371 |
| 12074_60hr_BF_Fcarcass | 0.0 |
| 11361_72hr_BF_Fcarcass | 0.0 |
| 11359_NBF_Ovaries | 0.0 |
| 12hr BF ovaries_11511_FPKM | 0.0 |
| 11512_24hr_BF_Ovaries | 0.0 |
| 11513_36hr_BF_Ovaries | 0.0 |
| 11514_48hr_BF_ovaries | 722.38 |
| 11515_60hr_BF_ovaries | 96.9139 |
| 11360_72hr_BF_Ovaries | 0.0946473 |
| 11362_0-2Hr_Embryos | 0.0 |
| 11507_2-4hr_Embryos | 0.0 |
| 11508_4-8hr_Embryos | 0.0753495 |
| 11509_8-12hr_Embryos | 0.0 |
| 11724_12-16hr_embryos | 0.0 |
| 11772_16-20hr_Embryos | 0.0 |
| 11773_20-24hr_embryos | 0.0547262 |
| 12105_24-28hr_Embryos | 0.0 |
| 12170_28-32hr_embryos | 0.0584524 |
| 12171_32-36hr_embryos | 0.0 |
| 11774_36-40hr_embryos | 0.115406 |
| 11775_40-44hr_embryos | 0.115039 |
| 11776_44-48hr_embryos | 0.0 |
| 12172_48-52hr_embryos | 0.0 |
| 12173_52-56hr_embryos | 0.0608802 |
| 12106_56-60hr_Embryos | 0.0 |
| 11777_60-64hr_embryos | 0.0 |
| 12174_64-68hr_embryos | 0.0 |
| 12175_68-72hr_embryos | 0.0 |
| 12107_72-76hr_embryos | 0.0845288 |
| 11782_1st_Instar_larvae | 0.0 |
| 11783_2nd_Instar_larvae | 0.0 |
| 11784_3rd_Instar_Larvae | 0.0608868 |
| 11363_4th_instar_larvae | 0.0969487 |
| 11725_MalePupae | 0.0 |
| 11666_FemalePupae | 0.0 |
### Chart:
| Category | AAEL003513-RA |
|---|---|
| 11357_MaleTestes | 0.0727806 |
| 11356_MaleCarcass | 0.123922 |
| 11358_NBF_Fcarcass | 0.0 |
| 11780_12hr_BF_Fcarcass | 0.0 |
| 11781_24hr_BF_carcass | 0.0 |
| 12072_36hr_BF_Fcarcass | 0.0 |
| 12073_48hr_BF_Fcarcass | 14.2339 |
| 12074_60hr_BF_Fcarcass | 0.0 |
| 11361_72hr_BF_Fcarcass | 0.0 |
| 11359_NBF_Ovaries | 0.0 |
| 12hr BF ovaries_11511_FPKM | 0.0 |
| 11512_24hr_BF_Ovaries | 0.0 |
| 11513_36hr_BF_Ovaries | 0.0 |
| 11514_48hr_BF_ovaries | 736.451 |
| 11515_60hr_BF_ovaries | 99.225 |
| 11360_72hr_BF_Ovaries | 0.0 |
| 11362_0-2Hr_Embryos | 0.0 |
| 11507_2-4hr_Embryos | 0.0 |
| 11508_4-8hr_Embryos | 0.0 |
| 11509_8-12hr_Embryos | 0.0 |
| 11724_12-16hr_embryos | 0.102204 |
| 11772_16-20hr_Embryos | 0.0668707 |
| 11773_20-24hr_embryos | 0.252344 |
| 12105_24-28hr_Embryos | 0.201475 |
| 12170_28-32hr_embryos | 0.198009 |
| 12171_32-36hr_embryos | 0.521198 |
| 11774_36-40hr_embryos | 0.03258 |
| 11775_40-44hr_embryos | 0.324763 |
| 11776_44-48hr_embryos | 0.358477 |
| 12172_48-52hr_embryos | 0.0 |
| 12173_52-56hr_embryos | 0.137493 |
| 12106_56-60hr_Embryos | 0.127484 |
| 11777_60-64hr_embryos | 0.388117 |
| 12174_64-68hr_embryos | 0.292435 |
| 12175_68-72hr_embryos | 0.339732 |
| 12107_72-76hr_embryos | 0.0 |
| 11782_1st_Instar_larvae | 0.0 |
| 11783_2nd_Instar_larvae | 0.073669 |
| 11784_3rd_Instar_Larvae | 0.0 |
| 11363_4th_instar_larvae | 0.0 |
| 11725_MalePupae | 0.339732 |
| 11666_FemalePupae | 0.0 |Odorant Binding Proteins

## Slide 10
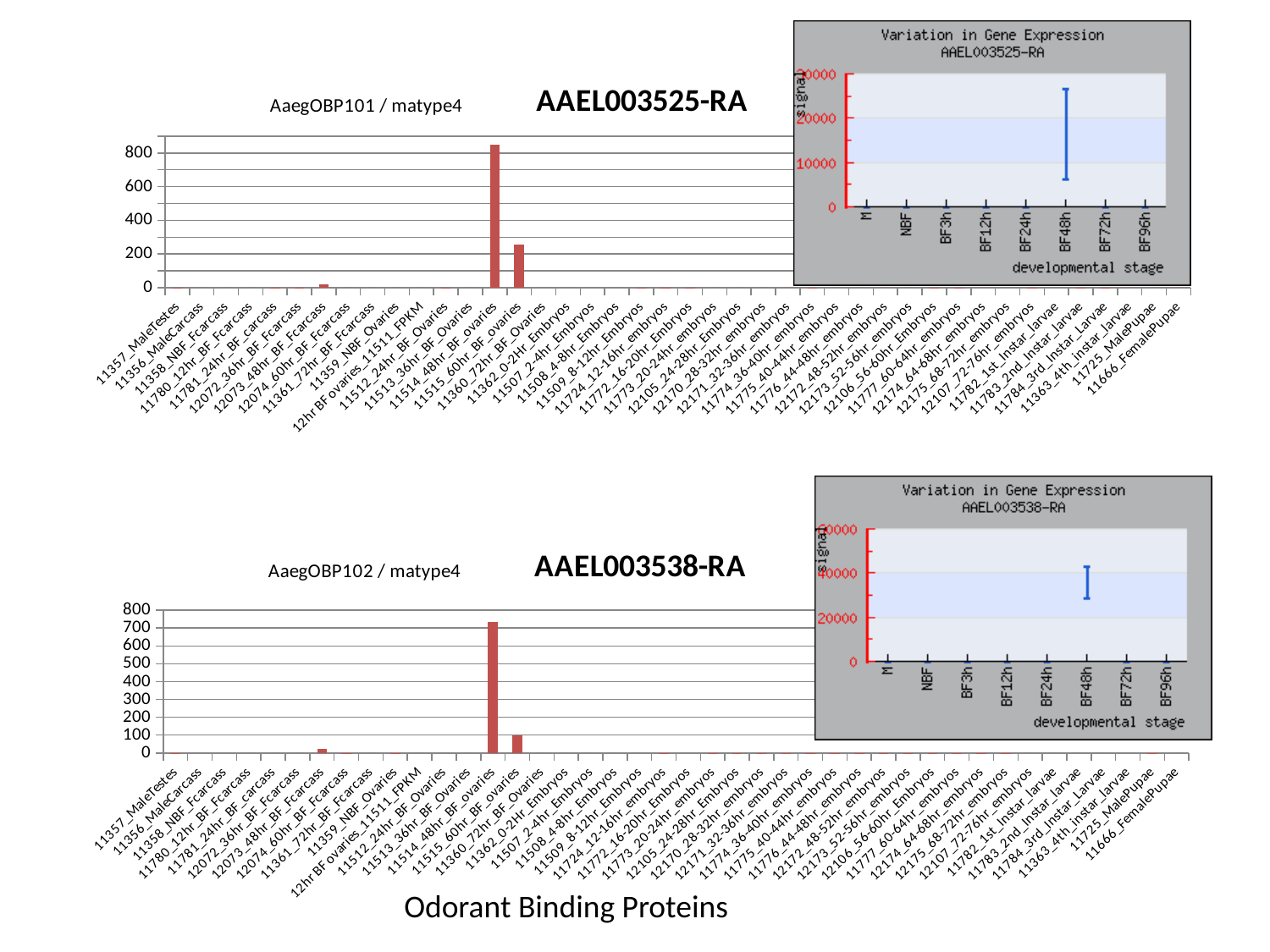

### Chart:
| Category | AAEL003525-RA |
|---|---|
| 11357_MaleTestes | 0.0351582 |
| 11356_MaleCarcass | 3.08817e-128 |
| 11358_NBF_Fcarcass | 0.0 |
| 11780_12hr_BF_Fcarcass | 0.0 |
| 11781_24hr_BF_carcass | 0.112775 |
| 12072_36hr_BF_Fcarcass | 0.0589659 |
| 12073_48hr_BF_Fcarcass | 17.1784 |
| 12074_60hr_BF_Fcarcass | 0.0 |
| 11361_72hr_BF_Fcarcass | 1.57863e-76 |
| 11359_NBF_Ovaries | 3.72306e-58 |
| 12hr BF ovaries_11511_FPKM | 0.0 |
| 11512_24hr_BF_Ovaries | 0.0513932 |
| 11513_36hr_BF_Ovaries | 1.5909e-145 |
| 11514_48hr_BF_ovaries | 846.986 |
| 11515_60hr_BF_ovaries | 256.755 |
| 11360_72hr_BF_Ovaries | 7.48514e-196 |
| 11362_0-2Hr_Embryos | 0.0 |
| 11507_2-4hr_Embryos | 0.0 |
| 11508_4-8hr_Embryos | 0.0 |
| 11509_8-12hr_Embryos | 0.0677056 |
| 11724_12-16hr_embryos | 0.0838654 |
| 11772_16-20hr_Embryos | 0.0529907 |
| 11773_20-24hr_embryos | 1.45839e-63 |
| 12105_24-28hr_Embryos | 4.53799e-80 |
| 12170_28-32hr_embryos | 1.57862e-172 |
| 12171_32-36hr_embryos | 0.0 |
| 11774_36-40hr_embryos | 0.0521275 |
| 11775_40-44hr_embryos | 9.31692e-170 |
| 11776_44-48hr_embryos | 1.55249e-123 |
| 12172_48-52hr_embryos | 3.23e-84 |
| 12173_52-56hr_embryos | 2.76199e-65 |
| 12106_56-60hr_Embryos | 0.09744 |
| 11777_60-64hr_embryos | 0.104265 |
| 12174_64-68hr_embryos | 1.47987e-116 |
| 12175_68-72hr_embryos | 0.0 |
| 12107_72-76hr_embryos | 0.0904036 |
| 11782_1st_Instar_larvae | 0.0 |
| 11783_2nd_Instar_larvae | 0.208302 |
| 11784_3rd_Instar_Larvae | 8.30543e-09 |
| 11363_4th_instar_larvae | 0.0 |
| 11725_MalePupae | 0.0 |
| 11666_FemalePupae | 0.0 |
### Chart:
| Category | AAEL003538-RA |
|---|---|
| 11357_MaleTestes | 0.221427 |
| 11356_MaleCarcass | 0.0 |
| 11358_NBF_Fcarcass | 0.0 |
| 11780_12hr_BF_Fcarcass | 0.0 |
| 11781_24hr_BF_carcass | 0.0 |
| 12072_36hr_BF_Fcarcass | 0.0 |
| 12073_48hr_BF_Fcarcass | 22.0854 |
| 12074_60hr_BF_Fcarcass | 0.039778 |
| 11361_72hr_BF_Fcarcass | 0.0 |
| 11359_NBF_Ovaries | 0.0896058 |
| 12hr BF ovaries_11511_FPKM | 0.0 |
| 11512_24hr_BF_Ovaries | 0.0 |
| 11513_36hr_BF_Ovaries | 0.0 |
| 11514_48hr_BF_ovaries | 736.32 |
| 11515_60hr_BF_ovaries | 99.0601 |
| 11360_72hr_BF_Ovaries | 0.0 |
| 11362_0-2Hr_Embryos | 0.0 |
| 11507_2-4hr_Embryos | 0.0 |
| 11508_4-8hr_Embryos | 0.0 |
| 11509_8-12hr_Embryos | 0.0 |
| 11724_12-16hr_embryos | 0.0690989 |
| 11772_16-20hr_Embryos | 0.0 |
| 11773_20-24hr_embryos | 0.130566 |
| 12105_24-28hr_Embryos | 0.136221 |
| 12170_28-32hr_embryos | 0.133891 |
| 12171_32-36hr_embryos | 0.253347 |
| 11774_36-40hr_embryos | 0.137668 |
| 11775_40-44hr_embryos | 0.395222 |
| 11776_44-48hr_embryos | 0.718963 |
| 12172_48-52hr_embryos | 0.0923291 |
| 12173_52-56hr_embryos | 0.325367 |
| 12106_56-60hr_Embryos | 0.560237 |
| 11777_60-64hr_embryos | 1.34816 |
| 12174_64-68hr_embryos | 0.437307 |
| 12175_68-72hr_embryos | 0.454965 |
| 12107_72-76hr_embryos | 0.0 |
| 11782_1st_Instar_larvae | 0.0 |
| 11783_2nd_Instar_larvae | 0.0 |
| 11784_3rd_Instar_Larvae | 0.0 |
| 11363_4th_instar_larvae | 0.0 |
| 11725_MalePupae | 0.454965 |
| 11666_FemalePupae | 0.0 |Odorant Binding Proteins

## Slide 11
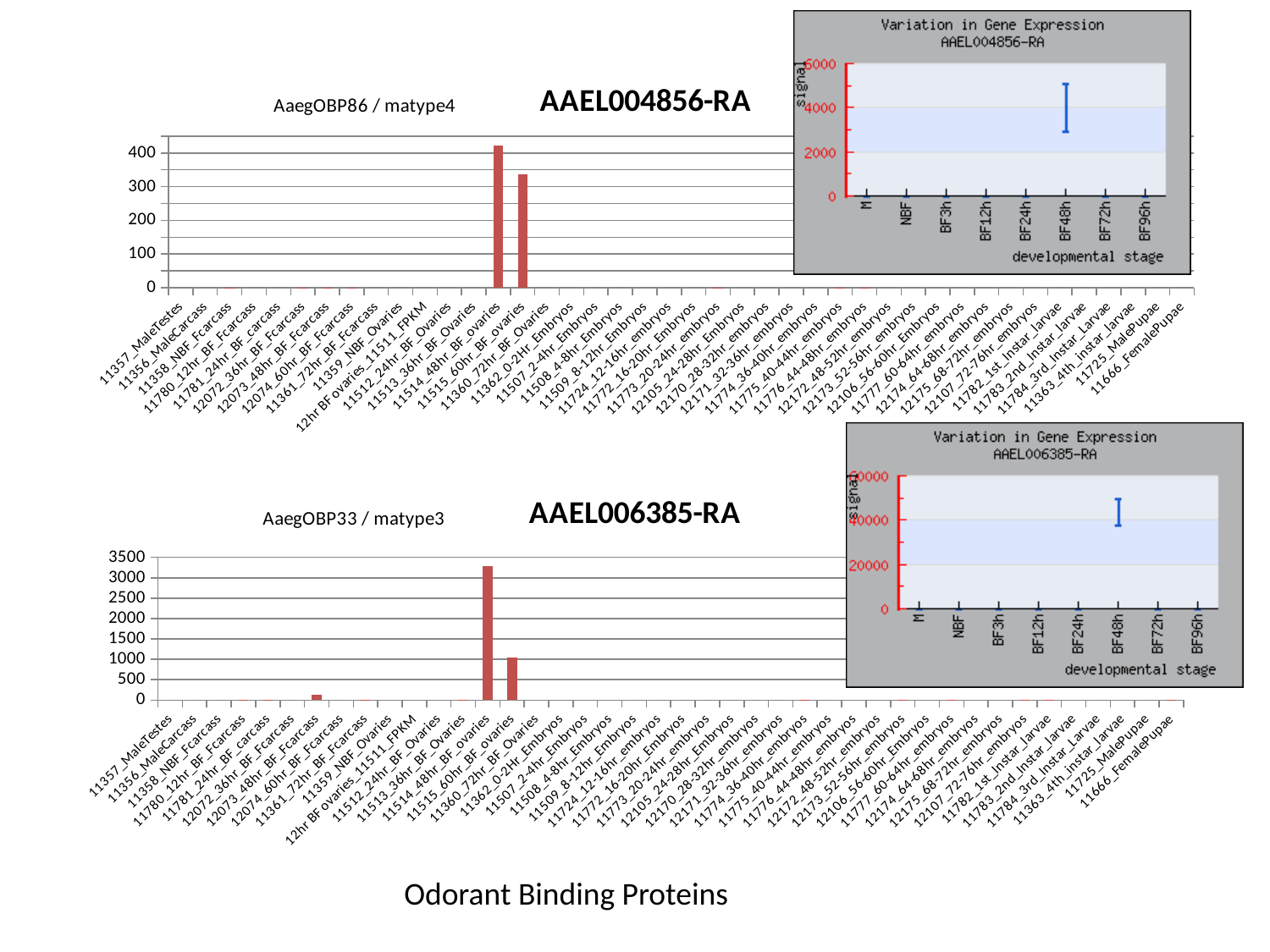

### Chart:
| Category | AAEL004856-RA |
|---|---|
| 11357_MaleTestes | 0.0 |
| 11356_MaleCarcass | 0.0 |
| 11358_NBF_Fcarcass | 0.114191 |
| 11780_12hr_BF_Fcarcass | 0.0 |
| 11781_24hr_BF_carcass | 0.0 |
| 12072_36hr_BF_Fcarcass | 0.158139 |
| 12073_48hr_BF_Fcarcass | 0.0579085 |
| 12074_60hr_BF_Fcarcass | 0.280031 |
| 11361_72hr_BF_Fcarcass | 0.0 |
| 11359_NBF_Ovaries | 0.0 |
| 12hr BF ovaries_11511_FPKM | 0.0 |
| 11512_24hr_BF_Ovaries | 0.0 |
| 11513_36hr_BF_Ovaries | 0.0 |
| 11514_48hr_BF_ovaries | 423.229 |
| 11515_60hr_BF_ovaries | 336.261 |
| 11360_72hr_BF_Ovaries | 0.0 |
| 11362_0-2Hr_Embryos | 0.0 |
| 11507_2-4hr_Embryos | 0.0 |
| 11508_4-8hr_Embryos | 0.0 |
| 11509_8-12hr_Embryos | 0.0 |
| 11724_12-16hr_embryos | 0.0 |
| 11772_16-20hr_Embryos | 0.0 |
| 11773_20-24hr_embryos | 0.0588315 |
| 12105_24-28hr_Embryos | 0.0 |
| 12170_28-32hr_embryos | 0.0 |
| 12171_32-36hr_embryos | 0.0 |
| 11774_36-40hr_embryos | 0.0 |
| 11775_40-44hr_embryos | 0.185503 |
| 11776_44-48hr_embryos | 0.0674107 |
| 12172_48-52hr_embryos | 0.0 |
| 12173_52-56hr_embryos | 0.0 |
| 12106_56-60hr_Embryos | 0.0 |
| 11777_60-64hr_embryos | 0.0 |
| 12174_64-68hr_embryos | 0.0 |
| 12175_68-72hr_embryos | 0.0 |
| 12107_72-76hr_embryos | 0.0 |
| 11782_1st_Instar_larvae | 0.0 |
| 11783_2nd_Instar_larvae | 0.0 |
| 11784_3rd_Instar_Larvae | 0.0 |
| 11363_4th_instar_larvae | 0.0 |
| 11725_MalePupae | 0.0 |
| 11666_FemalePupae | 0.0 |
### Chart:
| Category | AAEL006385-RA |
|---|---|
| 11357_MaleTestes | 0.0 |
| 11356_MaleCarcass | 0.0 |
| 11358_NBF_Fcarcass | 0.0 |
| 11780_12hr_BF_Fcarcass | 0.0598814 |
| 11781_24hr_BF_carcass | 0.0322708 |
| 12072_36hr_BF_Fcarcass | 0.0 |
| 12073_48hr_BF_Fcarcass | 140.503 |
| 12074_60hr_BF_Fcarcass | 0.0 |
| 11361_72hr_BF_Fcarcass | 0.0994886 |
| 11359_NBF_Ovaries | 0.0 |
| 12hr BF ovaries_11511_FPKM | 0.0 |
| 11512_24hr_BF_Ovaries | 0.0 |
| 11513_36hr_BF_Ovaries | 0.145084 |
| 11514_48hr_BF_ovaries | 3296.34 |
| 11515_60hr_BF_ovaries | 1046.58 |
| 11360_72hr_BF_Ovaries | 0.0 |
| 11362_0-2Hr_Embryos | 0.0 |
| 11507_2-4hr_Embryos | 0.0 |
| 11508_4-8hr_Embryos | 0.0 |
| 11509_8-12hr_Embryos | 0.0 |
| 11724_12-16hr_embryos | 0.0 |
| 11772_16-20hr_Embryos | 0.0 |
| 11773_20-24hr_embryos | 0.0 |
| 12105_24-28hr_Embryos | 0.0 |
| 12170_28-32hr_embryos | 0.0 |
| 12171_32-36hr_embryos | 0.0 |
| 11774_36-40hr_embryos | 0.0559083 |
| 11775_40-44hr_embryos | 0.0 |
| 11776_44-48hr_embryos | 0.0 |
| 12172_48-52hr_embryos | 0.0 |
| 12173_52-56hr_embryos | 0.0196624 |
| 12106_56-60hr_Embryos | 0.0 |
| 11777_60-64hr_embryos | 0.03289 |
| 12174_64-68hr_embryos | 0.0 |
| 12175_68-72hr_embryos | 0.0 |
| 12107_72-76hr_embryos | 0.232961 |
| 11782_1st_Instar_larvae | 0.0675695 |
| 11783_2nd_Instar_larvae | 0.0 |
| 11784_3rd_Instar_Larvae | 0.0 |
| 11363_4th_instar_larvae | 0.0 |
| 11725_MalePupae | 0.0 |
| 11666_FemalePupae | 0.0420672 |Odorant Binding Proteins

## Slide 12
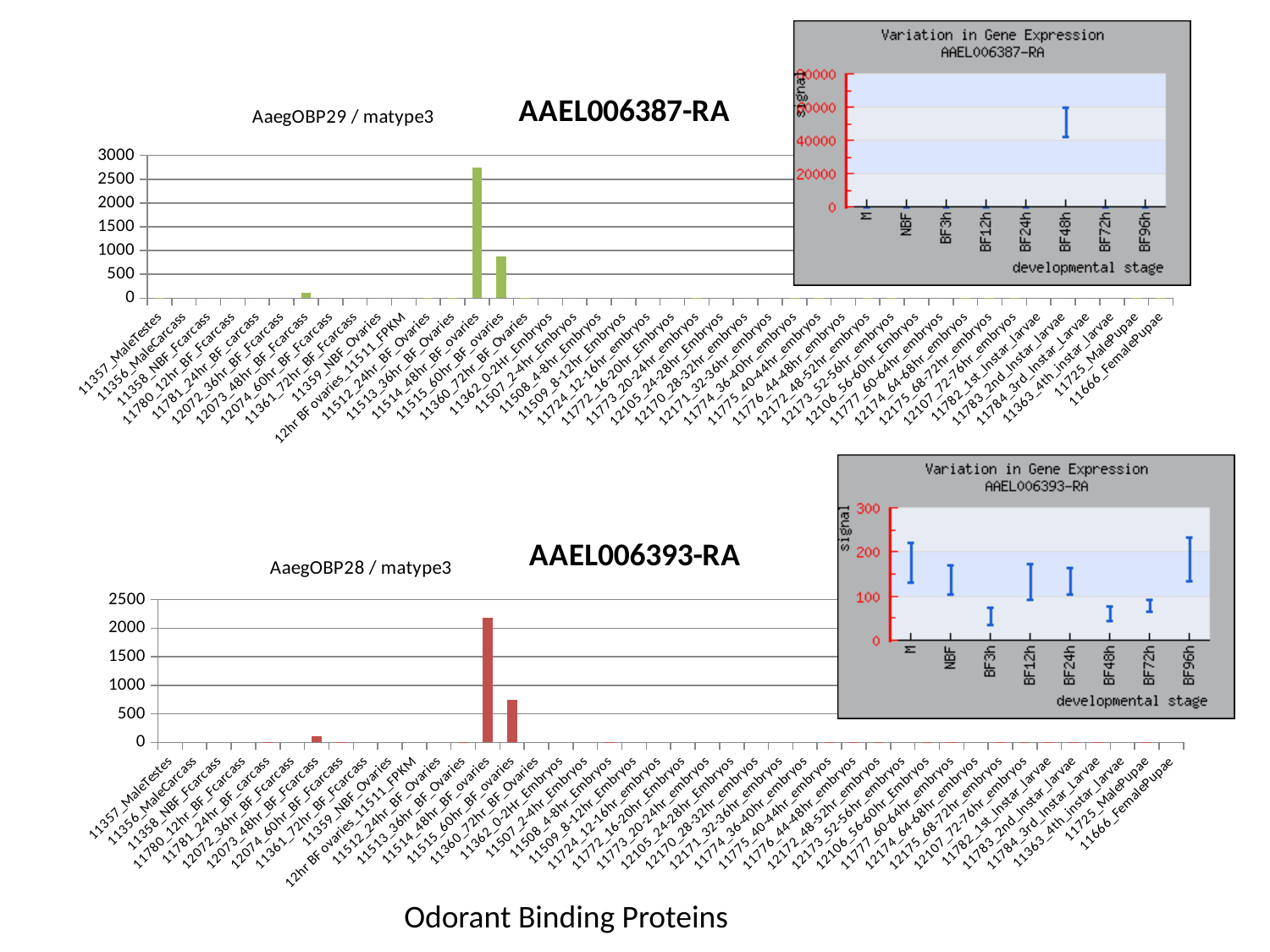

### Chart:
| Category | AAEL006387-RA |
|---|---|
| 11357_MaleTestes | 0.144175 |
| 11356_MaleCarcass | 0.0 |
| 11358_NBF_Fcarcass | 0.0 |
| 11780_12hr_BF_Fcarcass | 0.0 |
| 11781_24hr_BF_carcass | 3.20891e-67 |
| 12072_36hr_BF_Fcarcass | 0.0 |
| 12073_48hr_BF_Fcarcass | 115.193 |
| 12074_60hr_BF_Fcarcass | 1.19588e-44 |
| 11361_72hr_BF_Fcarcass | 0.0 |
| 11359_NBF_Ovaries | 0.0 |
| 12hr BF ovaries_11511_FPKM | 3.28041e-24 |
| 11512_24hr_BF_Ovaries | 0.0398659 |
| 11513_36hr_BF_Ovaries | 1.45361 |
| 11514_48hr_BF_ovaries | 2737.6 |
| 11515_60hr_BF_ovaries | 870.942 |
| 11360_72hr_BF_Ovaries | 0.103497 |
| 11362_0-2Hr_Embryos | 0.0 |
| 11507_2-4hr_Embryos | 6.96362e-18 |
| 11508_4-8hr_Embryos | 0.0 |
| 11509_8-12hr_Embryos | 0.0 |
| 11724_12-16hr_embryos | 0.0 |
| 11772_16-20hr_Embryos | 0.0 |
| 11773_20-24hr_embryos | 8.41128e-12 |
| 12105_24-28hr_Embryos | 0.0 |
| 12170_28-32hr_embryos | 0.0 |
| 12171_32-36hr_embryos | 0.0 |
| 11774_36-40hr_embryos | 0.0633172 |
| 11775_40-44hr_embryos | 0.166962 |
| 11776_44-48hr_embryos | 2.61373e-33 |
| 12172_48-52hr_embryos | 0.0570657 |
| 12173_52-56hr_embryos | 0.055441 |
| 12106_56-60hr_Embryos | 0.0 |
| 11777_60-64hr_embryos | 5.52198e-20 |
| 12174_64-68hr_embryos | 0.208537 |
| 12175_68-72hr_embryos | 1.09044e-11 |
| 12107_72-76hr_embryos | 0.318402 |
| 11782_1st_Instar_larvae | 2.81907e-13 |
| 11783_2nd_Instar_larvae | 5.79593e-31 |
| 11784_3rd_Instar_Larvae | 3.13363e-46 |
| 11363_4th_instar_larvae | 0.0 |
| 11725_MalePupae | 1.09044e-11 |
| 11666_FemalePupae | 0.330026 |
### Chart:
| Category | AAEL006393-RA |
|---|---|
| 11357_MaleTestes | 6.16912e-24 |
| 11356_MaleCarcass | 7.64882e-219 |
| 11358_NBF_Fcarcass | 0.0 |
| 11780_12hr_BF_Fcarcass | 3.13154e-105 |
| 11781_24hr_BF_carcass | 0.000159226 |
| 12072_36hr_BF_Fcarcass | 1.2975e-177 |
| 12073_48hr_BF_Fcarcass | 109.704 |
| 12074_60hr_BF_Fcarcass | 3.93056e-05 |
| 11361_72hr_BF_Fcarcass | 9.30586e-145 |
| 11359_NBF_Ovaries | 0.0 |
| 12hr BF ovaries_11511_FPKM | 1.23212e-74 |
| 11512_24hr_BF_Ovaries | 1.55235e-289 |
| 11513_36hr_BF_Ovaries | 0.343961 |
| 11514_48hr_BF_ovaries | 2179.15 |
| 11515_60hr_BF_ovaries | 743.949 |
| 11360_72hr_BF_Ovaries | 1.4097e-154 |
| 11362_0-2Hr_Embryos | 0.0 |
| 11507_2-4hr_Embryos | 9.87873e-36 |
| 11508_4-8hr_Embryos | 0.0707002 |
| 11509_8-12hr_Embryos | 0.0 |
| 11724_12-16hr_embryos | 0.0 |
| 11772_16-20hr_Embryos | 0.0 |
| 11773_20-24hr_embryos | 1.87864e-15 |
| 12105_24-28hr_Embryos | 0.0 |
| 12170_28-32hr_embryos | 2.40293e-37 |
| 12171_32-36hr_embryos | 1.95901e-220 |
| 11774_36-40hr_embryos | 2.98239e-30 |
| 11775_40-44hr_embryos | 0.308027 |
| 11776_44-48hr_embryos | 0.291046 |
| 12172_48-52hr_embryos | 0.206537 |
| 12173_52-56hr_embryos | 3.36705e-64 |
| 12106_56-60hr_Embryos | 7.02417e-12 |
| 11777_60-64hr_embryos | 0.35037 |
| 12174_64-68hr_embryos | 1.17656e-23 |
| 12175_68-72hr_embryos | 6.91664e-10 |
| 12107_72-76hr_embryos | 1.16311 |
| 11782_1st_Instar_larvae | 0.565513 |
| 11783_2nd_Instar_larvae | 0.202937 |
| 11784_3rd_Instar_Larvae | 0.0941009 |
| 11363_4th_instar_larvae | 0.0 |
| 11725_MalePupae | 6.91664e-10 |
| 11666_FemalePupae | 8.91288e-172 |Odorant Binding Proteins

## Slide 13
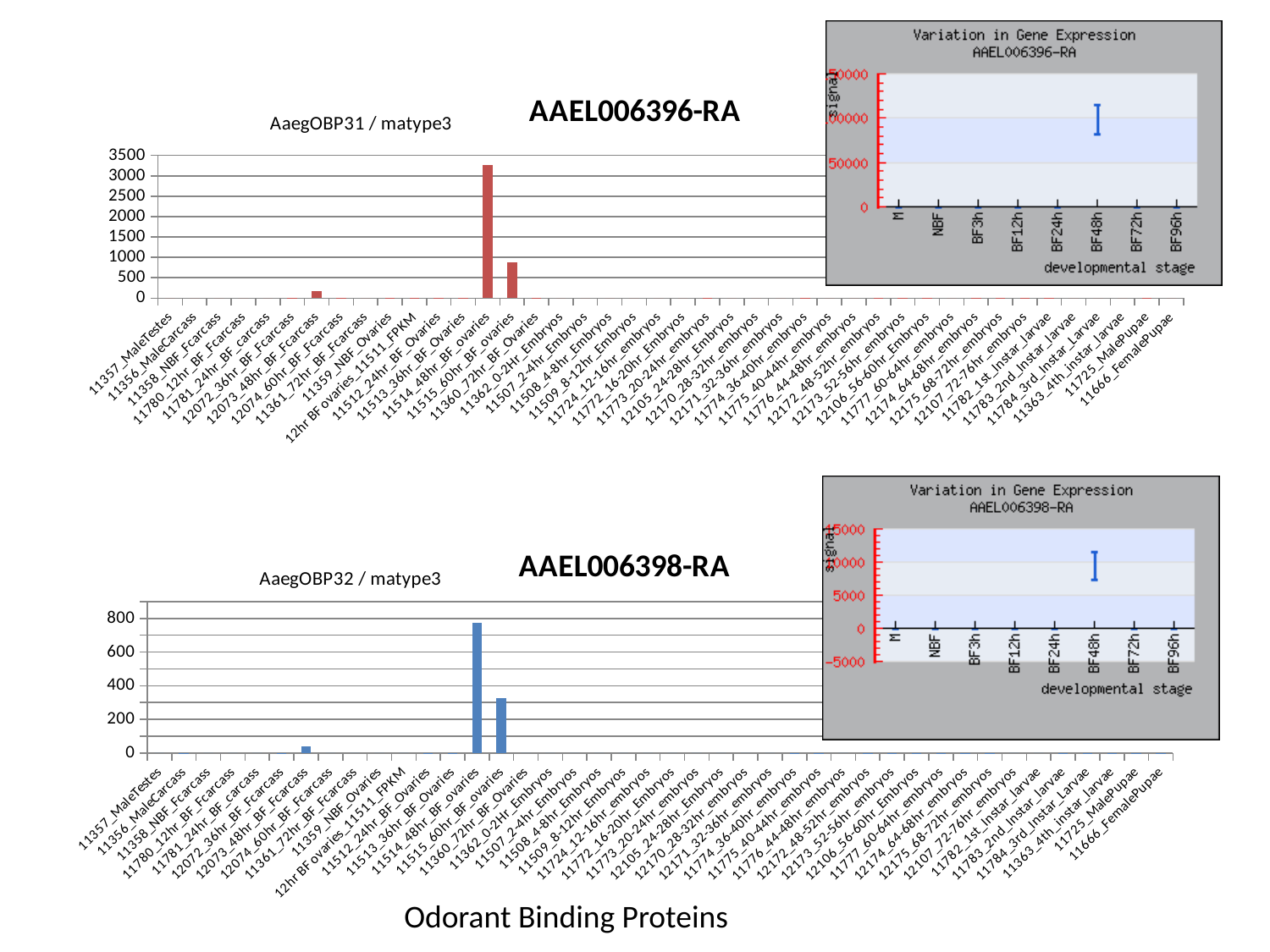

### Chart:
| Category | AAEL006396-RA |
|---|---|
| 11357_MaleTestes | 0.0 |
| 11356_MaleCarcass | 0.0 |
| 11358_NBF_Fcarcass | 0.0 |
| 11780_12hr_BF_Fcarcass | 0.0 |
| 11781_24hr_BF_carcass | 0.0 |
| 12072_36hr_BF_Fcarcass | 0.0664146 |
| 12073_48hr_BF_Fcarcass | 175.304 |
| 12074_60hr_BF_Fcarcass | 0.0470448 |
| 11361_72hr_BF_Fcarcass | 0.0 |
| 11359_NBF_Ovaries | 0.105979 |
| 12hr BF ovaries_11511_FPKM | 0.196708 |
| 11512_24hr_BF_Ovaries | 0.383683 |
| 11513_36hr_BF_Ovaries | 1.0817 |
| 11514_48hr_BF_ovaries | 3273.86 |
| 11515_60hr_BF_ovaries | 882.699 |
| 11360_72hr_BF_Ovaries | 0.0854632 |
| 11362_0-2Hr_Embryos | 0.0 |
| 11507_2-4hr_Embryos | 0.0 |
| 11508_4-8hr_Embryos | 0.0 |
| 11509_8-12hr_Embryos | 0.0 |
| 11724_12-16hr_embryos | 0.0 |
| 11772_16-20hr_Embryos | 0.0 |
| 11773_20-24hr_embryos | 0.0247079 |
| 12105_24-28hr_Embryos | 0.0 |
| 12170_28-32hr_embryos | 0.0 |
| 12171_32-36hr_embryos | 0.0 |
| 11774_36-40hr_embryos | 0.0521037 |
| 11775_40-44hr_embryos | 0.0 |
| 11776_44-48hr_embryos | 0.0 |
| 12172_48-52hr_embryos | 0.0272985 |
| 12173_52-56hr_embryos | 0.0458112 |
| 12106_56-60hr_Embryos | 0.0509699 |
| 11777_60-64hr_embryos | 0.0 |
| 12174_64-68hr_embryos | 0.0572685 |
| 12175_68-72hr_embryos | 0.0268305 |
| 12107_72-76hr_embryos | 0.141347 |
| 11782_1st_Instar_larvae | 0.031486 |
| 11783_2nd_Instar_larvae | 0.0 |
| 11784_3rd_Instar_Larvae | 0.0 |
| 11363_4th_instar_larvae | 0.0 |
| 11725_MalePupae | 0.0268305 |
| 11666_FemalePupae | 0.0 |
### Chart:
| Category | AAEL006398-RA |
|---|---|
| 11357_MaleTestes | 0.0 |
| 11356_MaleCarcass | 0.0972566 |
| 11358_NBF_Fcarcass | 0.0 |
| 11780_12hr_BF_Fcarcass | 0.0 |
| 11781_24hr_BF_carcass | 0.0 |
| 12072_36hr_BF_Fcarcass | 0.0651824 |
| 12073_48hr_BF_Fcarcass | 40.1965 |
| 12074_60hr_BF_Fcarcass | 0.0 |
| 11361_72hr_BF_Fcarcass | 0.0 |
| 11359_NBF_Ovaries | 0.0 |
| 12hr BF ovaries_11511_FPKM | 0.0 |
| 11512_24hr_BF_Ovaries | 0.215182 |
| 11513_36hr_BF_Ovaries | 1.17959 |
| 11514_48hr_BF_ovaries | 773.02 |
| 11515_60hr_BF_ovaries | 326.713 |
| 11360_72hr_BF_Ovaries | 0.0 |
| 11362_0-2Hr_Embryos | 0.0 |
| 11507_2-4hr_Embryos | 0.0 |
| 11508_4-8hr_Embryos | 0.0 |
| 11509_8-12hr_Embryos | 0.0 |
| 11724_12-16hr_embryos | 0.0 |
| 11772_16-20hr_Embryos | 0.0 |
| 11773_20-24hr_embryos | 0.0 |
| 12105_24-28hr_Embryos | 0.0 |
| 12170_28-32hr_embryos | 0.0 |
| 12171_32-36hr_embryos | 0.0 |
| 11774_36-40hr_embryos | 0.051137 |
| 11775_40-44hr_embryos | 0.0509743 |
| 11776_44-48hr_embryos | 0.0 |
| 12172_48-52hr_embryos | 0.214338 |
| 12173_52-56hr_embryos | 0.377676 |
| 12106_56-60hr_Embryos | 0.35017 |
| 11777_60-64hr_embryos | 0.240665 |
| 12174_64-68hr_embryos | 0.0562062 |
| 12175_68-72hr_embryos | 0.0526654 |
| 12107_72-76hr_embryos | 0.0 |
| 11782_1st_Instar_larvae | 0.0 |
| 11783_2nd_Instar_larvae | 0.0578168 |
| 11784_3rd_Instar_Larvae | 0.755432 |
| 11363_4th_instar_larvae | 0.515503 |
| 11725_MalePupae | 0.0526654 |
| 11666_FemalePupae | 0.692588 |Odorant Binding Proteins

## Slide 14
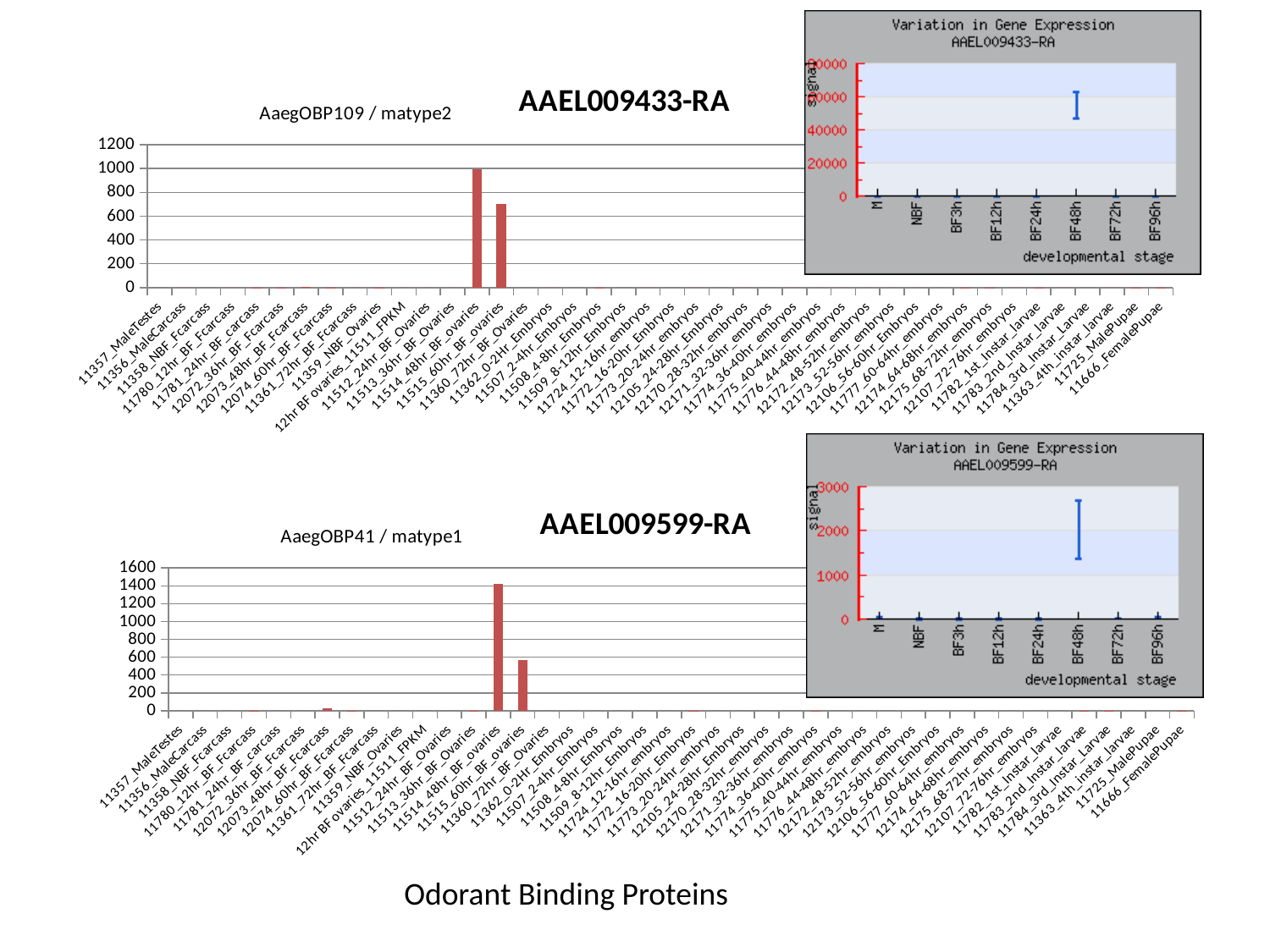

### Chart:
| Category | AAEL009433-RA |
|---|---|
| 11357_MaleTestes | 0.0 |
| 11356_MaleCarcass | 0.0 |
| 11358_NBF_Fcarcass | 0.0 |
| 11780_12hr_BF_Fcarcass | 0.0 |
| 11781_24hr_BF_carcass | 0.0320111 |
| 12072_36hr_BF_Fcarcass | 0.0353453 |
| 12073_48hr_BF_Fcarcass | 6.93757 |
| 12074_60hr_BF_Fcarcass | 0.112664 |
| 11361_72hr_BF_Fcarcass | 0.0 |
| 11359_NBF_Ovaries | 0.794321 |
| 12hr BF ovaries_11511_FPKM | 0.0 |
| 11512_24hr_BF_Ovaries | 0.0 |
| 11513_36hr_BF_Ovaries | 0.0 |
| 11514_48hr_BF_ovaries | 994.903 |
| 11515_60hr_BF_ovaries | 703.294 |
| 11360_72hr_BF_Ovaries | 0.0 |
| 11362_0-2Hr_Embryos | 0.0 |
| 11507_2-4hr_Embryos | 0.0 |
| 11508_4-8hr_Embryos | 0.0362092 |
| 11509_8-12hr_Embryos | 0.0 |
| 11724_12-16hr_embryos | 0.0 |
| 11772_16-20hr_Embryos | 0.0 |
| 11773_20-24hr_embryos | 0.0 |
| 12105_24-28hr_Embryos | 0.0 |
| 12170_28-32hr_embryos | 0.0 |
| 12171_32-36hr_embryos | 0.0 |
| 11774_36-40hr_embryos | 0.0 |
| 11775_40-44hr_embryos | 0.0 |
| 11776_44-48hr_embryos | 0.0 |
| 12172_48-52hr_embryos | 0.0 |
| 12173_52-56hr_embryos | 0.0 |
| 12106_56-60hr_Embryos | 0.0 |
| 11777_60-64hr_embryos | 0.0 |
| 12174_64-68hr_embryos | 0.0304776 |
| 12175_68-72hr_embryos | 0.0285579 |
| 12107_72-76hr_embryos | 0.0 |
| 11782_1st_Instar_larvae | 0.0335129 |
| 11783_2nd_Instar_larvae | 0.0 |
| 11784_3rd_Instar_Larvae | 0.0 |
| 11363_4th_instar_larvae | 0.0 |
| 11725_MalePupae | 0.0285579 |
| 11666_FemalePupae | 0.187779 |
### Chart:
| Category | AAEL009599-RA |
|---|---|
| 11357_MaleTestes | 0.0 |
| 11356_MaleCarcass | 0.0 |
| 11358_NBF_Fcarcass | 0.0 |
| 11780_12hr_BF_Fcarcass | 0.0572493 |
| 11781_24hr_BF_carcass | 0.0 |
| 12072_36hr_BF_Fcarcass | 0.0 |
| 12073_48hr_BF_Fcarcass | 28.1431 |
| 12074_60hr_BF_Fcarcass | 0.0482609 |
| 11361_72hr_BF_Fcarcass | 0.0 |
| 11359_NBF_Ovaries | 0.0 |
| 12hr BF ovaries_11511_FPKM | 0.0 |
| 11512_24hr_BF_Ovaries | 0.0 |
| 11513_36hr_BF_Ovaries | 0.369888 |
| 11514_48hr_BF_ovaries | 1422.96 |
| 11515_60hr_BF_ovaries | 569.181 |
| 11360_72hr_BF_Ovaries | 0.0 |
| 11362_0-2Hr_Embryos | 0.0 |
| 11507_2-4hr_Embryos | 0.0 |
| 11508_4-8hr_Embryos | 0.0 |
| 11509_8-12hr_Embryos | 0.0 |
| 11724_12-16hr_embryos | 0.0 |
| 11772_16-20hr_Embryos | 0.109708 |
| 11773_20-24hr_embryos | 0.0 |
| 12105_24-28hr_Embryos | 0.0 |
| 12170_28-32hr_embryos | 0.0 |
| 12171_32-36hr_embryos | 0.0 |
| 11774_36-40hr_embryos | 0.0534509 |
| 11775_40-44hr_embryos | 0.0 |
| 11776_44-48hr_embryos | 0.0 |
| 12172_48-52hr_embryos | 0.0 |
| 12173_52-56hr_embryos | 0.0 |
| 12106_56-60hr_Embryos | 0.0 |
| 11777_60-64hr_embryos | 0.0 |
| 12174_64-68hr_embryos | 0.0 |
| 12175_68-72hr_embryos | 0.0 |
| 12107_72-76hr_embryos | 0.0 |
| 11782_1st_Instar_larvae | 0.0 |
| 11783_2nd_Instar_larvae | 0.543893 |
| 11784_3rd_Instar_Larvae | 0.225603 |
| 11363_4th_instar_larvae | 0.0 |
| 11725_MalePupae | 0.0 |
| 11666_FemalePupae | 0.241309 |Odorant Binding Proteins

## Slide 15
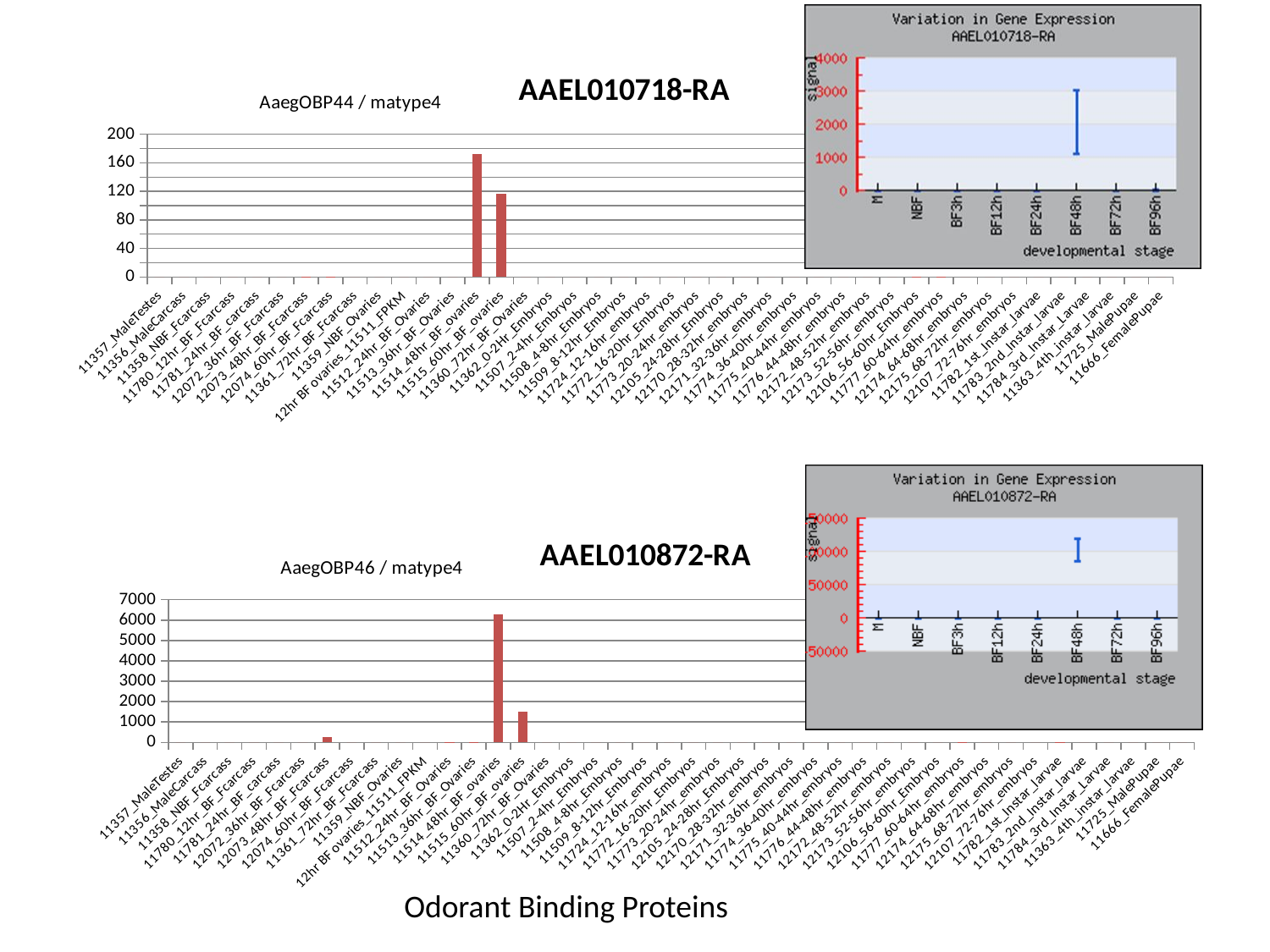

### Chart:
| Category | AAEL010718-RA |
|---|---|
| 11357_MaleTestes | 0.0 |
| 11356_MaleCarcass | 0.0 |
| 11358_NBF_Fcarcass | 0.0 |
| 11780_12hr_BF_Fcarcass | 0.0 |
| 11781_24hr_BF_carcass | 0.0 |
| 12072_36hr_BF_Fcarcass | 0.0 |
| 12073_48hr_BF_Fcarcass | 0.421779 |
| 12074_60hr_BF_Fcarcass | 0.0407896 |
| 11361_72hr_BF_Fcarcass | 0.0 |
| 11359_NBF_Ovaries | 0.0 |
| 12hr BF ovaries_11511_FPKM | 0.0 |
| 11512_24hr_BF_Ovaries | 0.0 |
| 11513_36hr_BF_Ovaries | 0.0 |
| 11514_48hr_BF_ovaries | 172.861 |
| 11515_60hr_BF_ovaries | 116.365 |
| 11360_72hr_BF_Ovaries | 0.0 |
| 11362_0-2Hr_Embryos | 0.0 |
| 11507_2-4hr_Embryos | 0.0 |
| 11508_4-8hr_Embryos | 0.0 |
| 11509_8-12hr_Embryos | 0.0 |
| 11724_12-16hr_embryos | 0.0 |
| 11772_16-20hr_Embryos | 0.0 |
| 11773_20-24hr_embryos | 0.0 |
| 12105_24-28hr_Embryos | 0.0 |
| 12170_28-32hr_embryos | 0.0 |
| 12171_32-36hr_embryos | 0.0 |
| 11774_36-40hr_embryos | 0.0 |
| 11775_40-44hr_embryos | 0.0 |
| 11776_44-48hr_embryos | 0.0 |
| 12172_48-52hr_embryos | 0.0 |
| 12173_52-56hr_embryos | 0.0 |
| 12106_56-60hr_Embryos | 0.0442009 |
| 11777_60-64hr_embryos | 0.0531622 |
| 12174_64-68hr_embryos | 0.0 |
| 12175_68-72hr_embryos | 0.0 |
| 12107_72-76hr_embryos | 0.0 |
| 11782_1st_Instar_larvae | 0.0 |
| 11783_2nd_Instar_larvae | 0.0 |
| 11784_3rd_Instar_Larvae | 0.0 |
| 11363_4th_instar_larvae | 0.0 |
| 11725_MalePupae | 0.0 |
| 11666_FemalePupae | 0.0 |
### Chart:
| Category | AAEL010872-RA |
|---|---|
| 11357_MaleTestes | 0.0 |
| 11356_MaleCarcass | 0.0 |
| 11358_NBF_Fcarcass | 0.0 |
| 11780_12hr_BF_Fcarcass | 0.0 |
| 11781_24hr_BF_carcass | 0.0 |
| 12072_36hr_BF_Fcarcass | 0.0 |
| 12073_48hr_BF_Fcarcass | 251.075 |
| 12074_60hr_BF_Fcarcass | 0.0 |
| 11361_72hr_BF_Fcarcass | 0.0 |
| 11359_NBF_Ovaries | 0.0 |
| 12hr BF ovaries_11511_FPKM | 0.0 |
| 11512_24hr_BF_Ovaries | 0.101445 |
| 11513_36hr_BF_Ovaries | 0.556099 |
| 11514_48hr_BF_ovaries | 6267.71 |
| 11515_60hr_BF_ovaries | 1498.95 |
| 11360_72hr_BF_Ovaries | 0.0 |
| 11362_0-2Hr_Embryos | 0.0 |
| 11507_2-4hr_Embryos | 0.0 |
| 11508_4-8hr_Embryos | 0.0 |
| 11509_8-12hr_Embryos | 0.0 |
| 11724_12-16hr_embryos | 0.0 |
| 11772_16-20hr_Embryos | 0.0 |
| 11773_20-24hr_embryos | 0.0 |
| 12105_24-28hr_Embryos | 0.0 |
| 12170_28-32hr_embryos | 0.0 |
| 12171_32-36hr_embryos | 0.0 |
| 11774_36-40hr_embryos | 0.0 |
| 11775_40-44hr_embryos | 0.0 |
| 11776_44-48hr_embryos | 0.0 |
| 12172_48-52hr_embryos | 0.0 |
| 12173_52-56hr_embryos | 0.0 |
| 12106_56-60hr_Embryos | 0.0 |
| 11777_60-64hr_embryos | 0.113456 |
| 12174_64-68hr_embryos | 0.0 |
| 12175_68-72hr_embryos | 0.0 |
| 12107_72-76hr_embryos | 0.0 |
| 11782_1st_Instar_larvae | 0.0582726 |
| 11783_2nd_Instar_larvae | 0.0 |
| 11784_3rd_Instar_Larvae | 0.0 |
| 11363_4th_instar_larvae | 0.0 |
| 11725_MalePupae | 0.0 |
| 11666_FemalePupae | 0.0 |Odorant Binding Proteins

## Slide 16
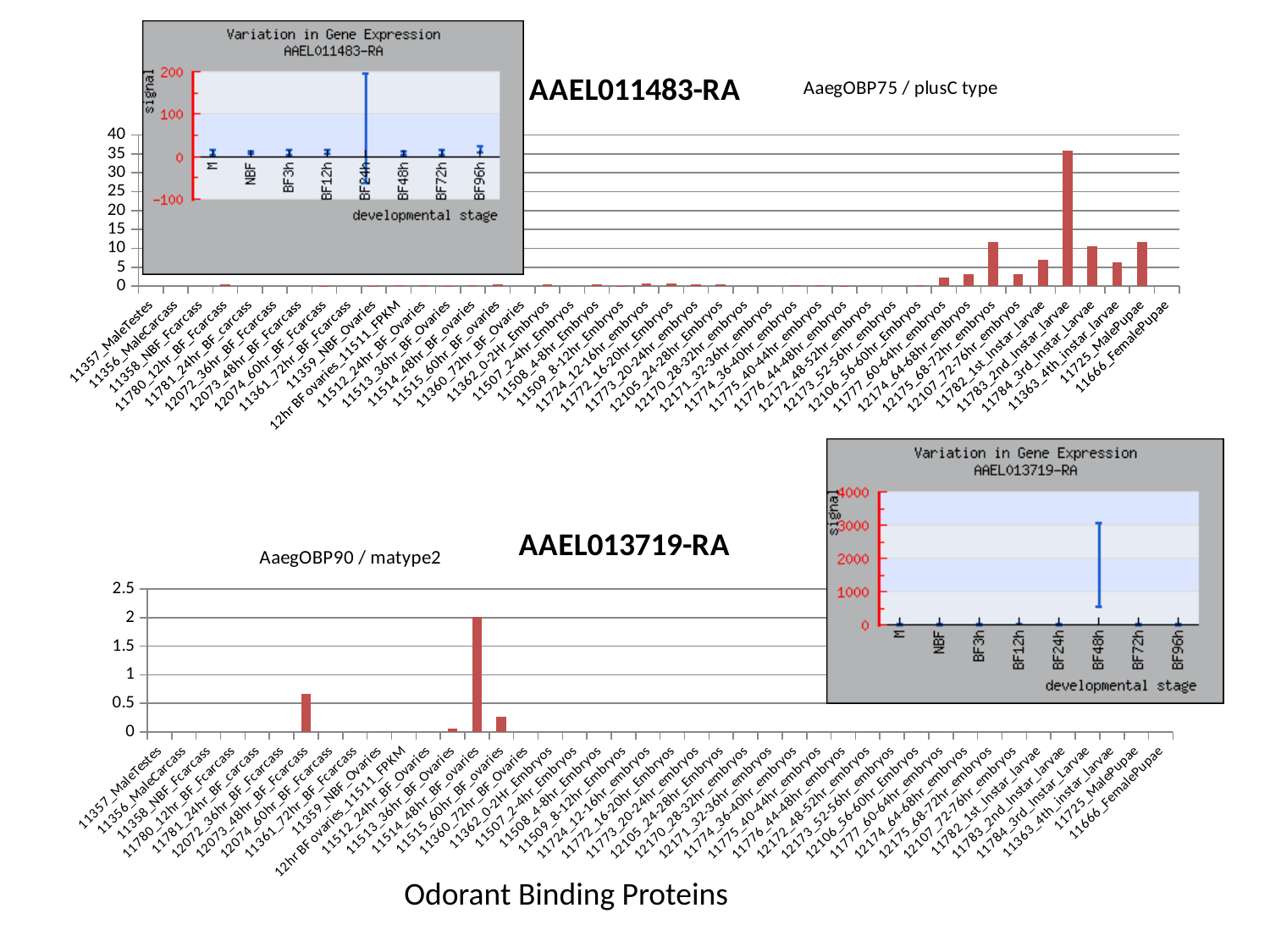

### Chart:
| Category | AAEL011483-RA |
|---|---|
| 11357_MaleTestes | 0.0 |
| 11356_MaleCarcass | 0.0 |
| 11358_NBF_Fcarcass | 0.0 |
| 11780_12hr_BF_Fcarcass | 0.377309 |
| 11781_24hr_BF_carcass | 0.0 |
| 12072_36hr_BF_Fcarcass | 0.0 |
| 12073_48hr_BF_Fcarcass | 0.0 |
| 12074_60hr_BF_Fcarcass | 8.31652e-05 |
| 11361_72hr_BF_Fcarcass | 0.0 |
| 11359_NBF_Ovaries | 0.0309451 |
| 12hr BF ovaries_11511_FPKM | 0.16419 |
| 11512_24hr_BF_Ovaries | 0.205812 |
| 11513_36hr_BF_Ovaries | 1.03204e-06 |
| 11514_48hr_BF_ovaries | 0.348547 |
| 11515_60hr_BF_ovaries | 0.471926 |
| 11360_72hr_BF_Ovaries | 0.0 |
| 11362_0-2Hr_Embryos | 0.582465 |
| 11507_2-4hr_Embryos | 0.0 |
| 11508_4-8hr_Embryos | 0.511117 |
| 11509_8-12hr_Embryos | 0.139987 |
| 11724_12-16hr_embryos | 0.751174 |
| 11772_16-20hr_Embryos | 0.627643 |
| 11773_20-24hr_embryos | 0.46403 |
| 12105_24-28hr_Embryos | 0.516335 |
| 12170_28-32hr_embryos | 6.25121e-30 |
| 12171_32-36hr_embryos | 0.0 |
| 11774_36-40hr_embryos | 0.293562 |
| 11775_40-44hr_embryos | 0.325113 |
| 11776_44-48hr_embryos | 6.4834e-07 |
| 12172_48-52hr_embryos | 0.0 |
| 12173_52-56hr_embryos | 0.0 |
| 12106_56-60hr_Embryos | 0.240879 |
| 11777_60-64hr_embryos | 2.20057 |
| 12174_64-68hr_embryos | 3.11277 |
| 12175_68-72hr_embryos | 11.6686 |
| 12107_72-76hr_embryos | 3.17983 |
| 11782_1st_Instar_larvae | 7.06637 |
| 11783_2nd_Instar_larvae | 35.872 |
| 11784_3rd_Instar_Larvae | 10.5441 |
| 11363_4th_instar_larvae | 6.31916 |
| 11725_MalePupae | 11.6686 |
| 11666_FemalePupae | 0.0 |
### Chart:
| Category | AAEL013719-RA |
|---|---|
| 11357_MaleTestes | 0.0 |
| 11356_MaleCarcass | 0.0 |
| 11358_NBF_Fcarcass | 0.0 |
| 11780_12hr_BF_Fcarcass | 0.0 |
| 11781_24hr_BF_carcass | 0.0 |
| 12072_36hr_BF_Fcarcass | 0.0 |
| 12073_48hr_BF_Fcarcass | 0.666559 |
| 12074_60hr_BF_Fcarcass | 0.0 |
| 11361_72hr_BF_Fcarcass | 0.0 |
| 11359_NBF_Ovaries | 0.0 |
| 12hr BF ovaries_11511_FPKM | 0.0 |
| 11512_24hr_BF_Ovaries | 0.0 |
| 11513_36hr_BF_Ovaries | 0.051469 |
| 11514_48hr_BF_ovaries | 2.01446 |
| 11515_60hr_BF_ovaries | 0.269016 |
| 11360_72hr_BF_Ovaries | 0.0 |
| 11362_0-2Hr_Embryos | 0.0 |
| 11507_2-4hr_Embryos | 0.0 |
| 11508_4-8hr_Embryos | 0.0 |
| 11509_8-12hr_Embryos | 0.0 |
| 11724_12-16hr_embryos | 0.0 |
| 11772_16-20hr_Embryos | 0.0 |
| 11773_20-24hr_embryos | 0.0 |
| 12105_24-28hr_Embryos | 0.0 |
| 12170_28-32hr_embryos | 0.0 |
| 12171_32-36hr_embryos | 0.0 |
| 11774_36-40hr_embryos | 0.0 |
| 11775_40-44hr_embryos | 0.0 |
| 11776_44-48hr_embryos | 0.0 |
| 12172_48-52hr_embryos | 0.0 |
| 12173_52-56hr_embryos | 0.0 |
| 12106_56-60hr_Embryos | 0.0 |
| 11777_60-64hr_embryos | 0.0 |
| 12174_64-68hr_embryos | 0.0 |
| 12175_68-72hr_embryos | 0.0 |
| 12107_72-76hr_embryos | 0.0 |
| 11782_1st_Instar_larvae | 0.0 |
| 11783_2nd_Instar_larvae | 0.0 |
| 11784_3rd_Instar_Larvae | 0.0 |
| 11363_4th_instar_larvae | 0.0 |
| 11725_MalePupae | 0.0 |
| 11666_FemalePupae | 0.0 |Odorant Binding Proteins

## Slide 17
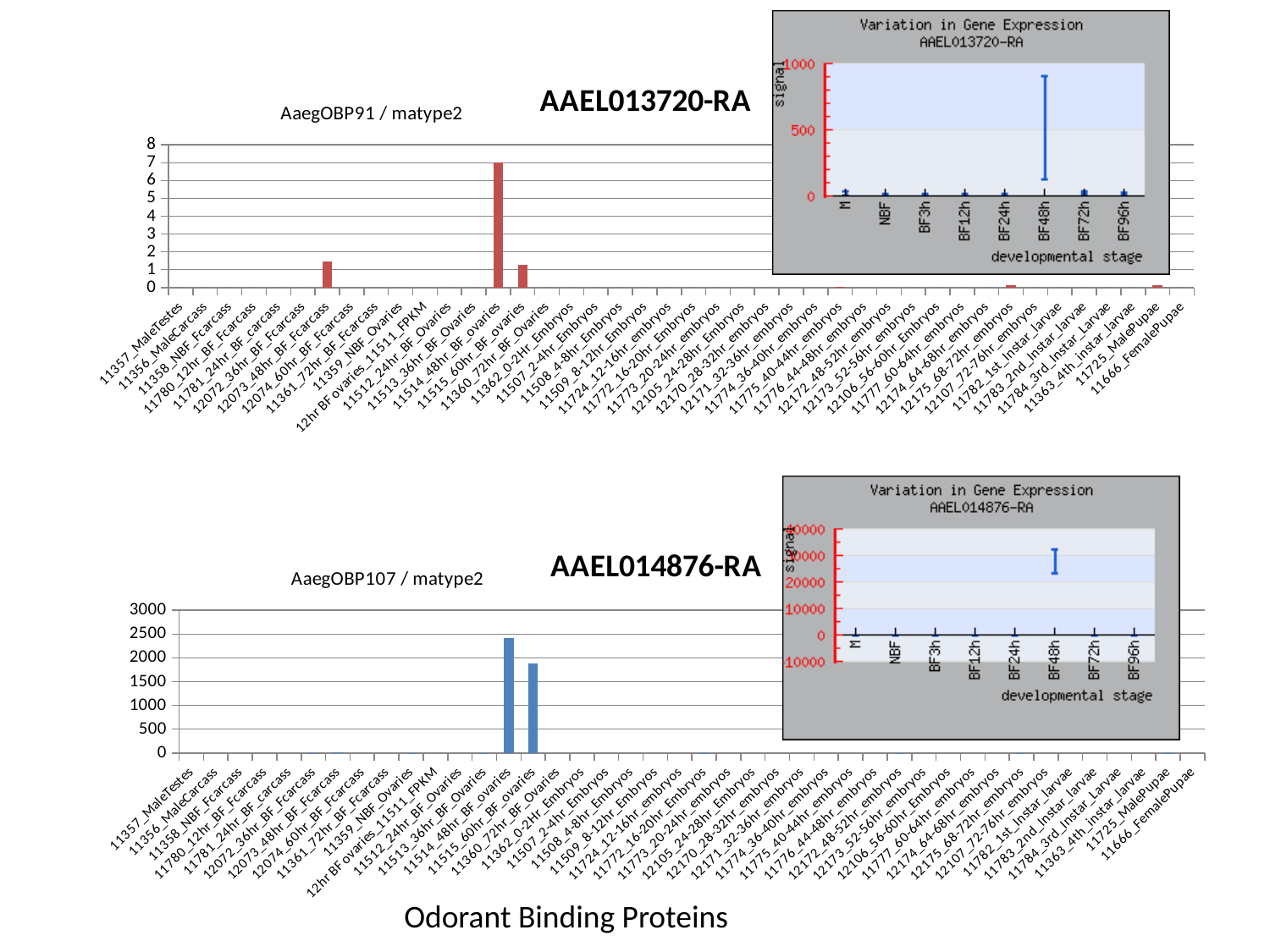

### Chart:
| Category | AAEL013720-RA |
|---|---|
| 11357_MaleTestes | 0.0 |
| 11356_MaleCarcass | 0.0 |
| 11358_NBF_Fcarcass | 0.0 |
| 11780_12hr_BF_Fcarcass | 0.0 |
| 11781_24hr_BF_carcass | 0.0 |
| 12072_36hr_BF_Fcarcass | 0.0 |
| 12073_48hr_BF_Fcarcass | 1.4653 |
| 12074_60hr_BF_Fcarcass | 0.0 |
| 11361_72hr_BF_Fcarcass | 0.0 |
| 11359_NBF_Ovaries | 0.0 |
| 12hr BF ovaries_11511_FPKM | 0.0 |
| 11512_24hr_BF_Ovaries | 0.0 |
| 11513_36hr_BF_Ovaries | 0.0 |
| 11514_48hr_BF_ovaries | 7.00919 |
| 11515_60hr_BF_ovaries | 1.28689 |
| 11360_72hr_BF_Ovaries | 0.0 |
| 11362_0-2Hr_Embryos | 0.0 |
| 11507_2-4hr_Embryos | 0.0 |
| 11508_4-8hr_Embryos | 0.0 |
| 11509_8-12hr_Embryos | 0.0 |
| 11724_12-16hr_embryos | 0.0 |
| 11772_16-20hr_Embryos | 0.0 |
| 11773_20-24hr_embryos | 0.0 |
| 12105_24-28hr_Embryos | 0.0 |
| 12170_28-32hr_embryos | 0.0 |
| 12171_32-36hr_embryos | 0.0 |
| 11774_36-40hr_embryos | 0.0 |
| 11775_40-44hr_embryos | 0.0625854 |
| 11776_44-48hr_embryos | 0.0 |
| 12172_48-52hr_embryos | 0.0 |
| 12173_52-56hr_embryos | 0.0 |
| 12106_56-60hr_Embryos | 0.0 |
| 11777_60-64hr_embryos | 0.0 |
| 12174_64-68hr_embryos | 0.0 |
| 12175_68-72hr_embryos | 0.129323 |
| 12107_72-76hr_embryos | 0.0 |
| 11782_1st_Instar_larvae | 0.0 |
| 11783_2nd_Instar_larvae | 0.0 |
| 11784_3rd_Instar_Larvae | 0.0 |
| 11363_4th_instar_larvae | 0.0 |
| 11725_MalePupae | 0.129323 |
| 11666_FemalePupae | 0.0 |
### Chart:
| Category | AAEL014876-RA |
|---|---|
| 11357_MaleTestes | 0.0 |
| 11356_MaleCarcass | 0.0 |
| 11358_NBF_Fcarcass | 0.0 |
| 11780_12hr_BF_Fcarcass | 0.0 |
| 11781_24hr_BF_carcass | 0.0 |
| 12072_36hr_BF_Fcarcass | 0.0712642 |
| 12073_48hr_BF_Fcarcass | 16.1276 |
| 12074_60hr_BF_Fcarcass | 0.0 |
| 11361_72hr_BF_Fcarcass | 0.0 |
| 11359_NBF_Ovaries | 0.113718 |
| 12hr BF ovaries_11511_FPKM | 0.0 |
| 11512_24hr_BF_Ovaries | 0.0 |
| 11513_36hr_BF_Ovaries | 0.128964 |
| 11514_48hr_BF_ovaries | 2411.58 |
| 11515_60hr_BF_ovaries | 1890.94 |
| 11360_72hr_BF_Ovaries | 0.0 |
| 11362_0-2Hr_Embryos | 0.0 |
| 11507_2-4hr_Embryos | 0.0 |
| 11508_4-8hr_Embryos | 0.0 |
| 11509_8-12hr_Embryos | 0.0 |
| 11724_12-16hr_embryos | 0.0 |
| 11772_16-20hr_Embryos | 0.0573761 |
| 11773_20-24hr_embryos | 0.0 |
| 12105_24-28hr_Embryos | 0.0 |
| 12170_28-32hr_embryos | 0.0 |
| 12171_32-36hr_embryos | 0.0 |
| 11774_36-40hr_embryos | 0.0 |
| 11775_40-44hr_embryos | 0.0 |
| 11776_44-48hr_embryos | 0.0 |
| 12172_48-52hr_embryos | 0.0585822 |
| 12173_52-56hr_embryos | 0.0 |
| 12106_56-60hr_Embryos | 0.0 |
| 11777_60-64hr_embryos | 0.0 |
| 12174_64-68hr_embryos | 0.0 |
| 12175_68-72hr_embryos | 0.0575793 |
| 12107_72-76hr_embryos | 0.0 |
| 11782_1st_Instar_larvae | 0.0 |
| 11783_2nd_Instar_larvae | 0.0 |
| 11784_3rd_Instar_Larvae | 0.0 |
| 11363_4th_instar_larvae | 0.0 |
| 11725_MalePupae | 0.0575793 |
| 11666_FemalePupae | 0.0 |Odorant Binding Proteins

## Slide 18
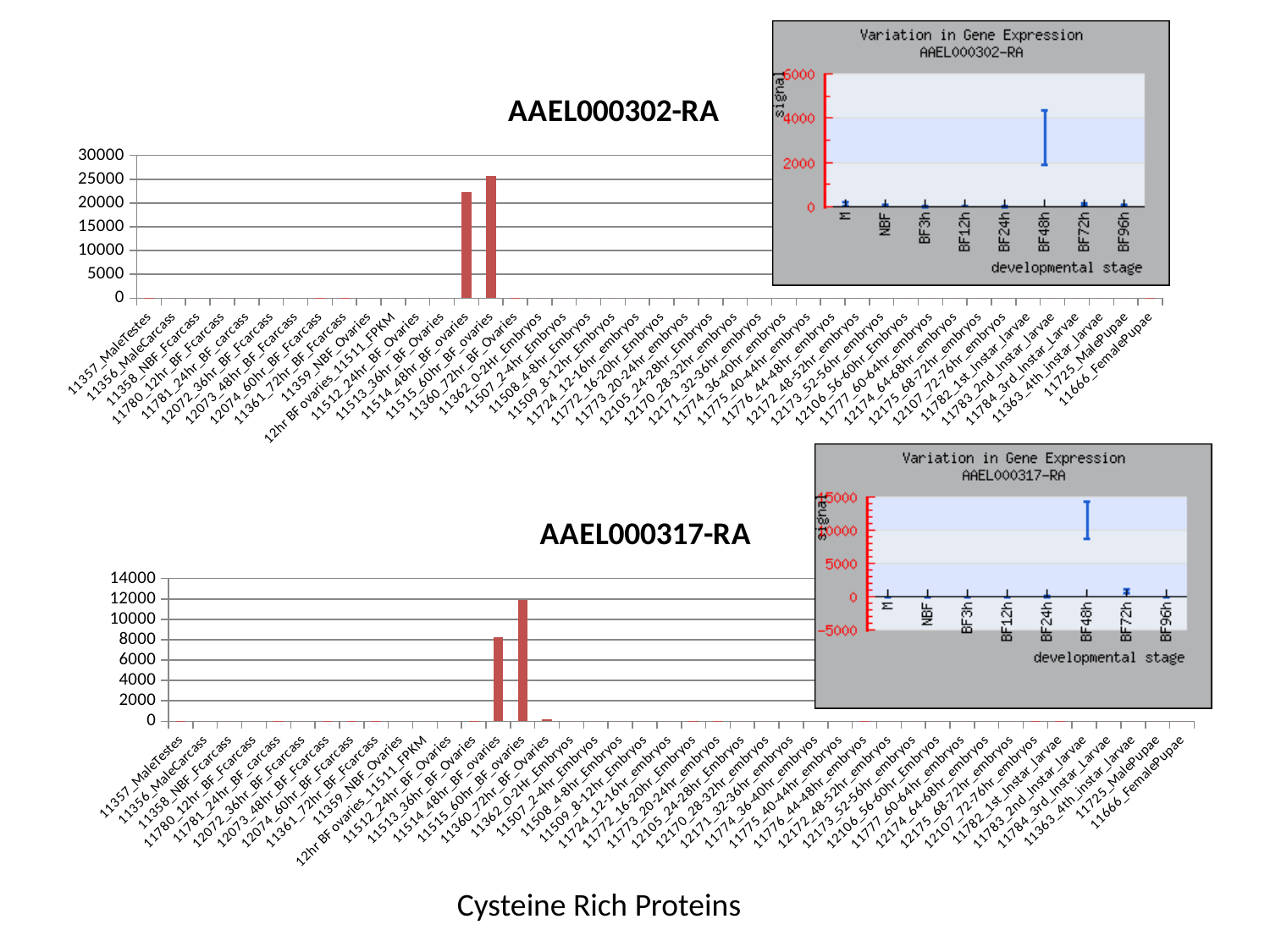

### Chart:
| Category | AAEL000302-RA |
|---|---|
| 11357_MaleTestes | 2.90719e-08 |
| 11356_MaleCarcass | 0.0 |
| 11358_NBF_Fcarcass | 0.0 |
| 11780_12hr_BF_Fcarcass | 0.0 |
| 11781_24hr_BF_carcass | 0.0 |
| 12072_36hr_BF_Fcarcass | 0.0 |
| 12073_48hr_BF_Fcarcass | 0.0 |
| 12074_60hr_BF_Fcarcass | 34.3305 |
| 11361_72hr_BF_Fcarcass | 2.06865 |
| 11359_NBF_Ovaries | 0.0 |
| 12hr BF ovaries_11511_FPKM | 0.0 |
| 11512_24hr_BF_Ovaries | 0.0 |
| 11513_36hr_BF_Ovaries | 0.0 |
| 11514_48hr_BF_ovaries | 22353.3 |
| 11515_60hr_BF_ovaries | 25699.2 |
| 11360_72hr_BF_Ovaries | 55.1165 |
| 11362_0-2Hr_Embryos | 0.0 |
| 11507_2-4hr_Embryos | 0.0 |
| 11508_4-8hr_Embryos | 0.0 |
| 11509_8-12hr_Embryos | 0.0 |
| 11724_12-16hr_embryos | 0.0 |
| 11772_16-20hr_Embryos | 0.0 |
| 11773_20-24hr_embryos | 0.0 |
| 12105_24-28hr_Embryos | 0.0 |
| 12170_28-32hr_embryos | 0.0 |
| 12171_32-36hr_embryos | 0.0 |
| 11774_36-40hr_embryos | 0.0 |
| 11775_40-44hr_embryos | 0.0 |
| 11776_44-48hr_embryos | 0.0 |
| 12172_48-52hr_embryos | 0.0 |
| 12173_52-56hr_embryos | 0.0 |
| 12106_56-60hr_Embryos | 0.0 |
| 11777_60-64hr_embryos | 0.0 |
| 12174_64-68hr_embryos | 0.0 |
| 12175_68-72hr_embryos | 0.0 |
| 12107_72-76hr_embryos | 0.0 |
| 11782_1st_Instar_larvae | 0.0 |
| 11783_2nd_Instar_larvae | 0.0 |
| 11784_3rd_Instar_Larvae | 0.0 |
| 11363_4th_instar_larvae | 0.0 |
| 11725_MalePupae | 0.0 |
| 11666_FemalePupae | 0.673071 |
### Chart:
| Category | AAEL000317-RA |
|---|---|
| 11357_MaleTestes | 29.5093 |
| 11356_MaleCarcass | 0.0 |
| 11358_NBF_Fcarcass | 0.0 |
| 11780_12hr_BF_Fcarcass | 0.0 |
| 11781_24hr_BF_carcass | 0.300126 |
| 12072_36hr_BF_Fcarcass | 0.0 |
| 12073_48hr_BF_Fcarcass | 0.849044 |
| 12074_60hr_BF_Fcarcass | 22.4609 |
| 11361_72hr_BF_Fcarcass | 7.97854 |
| 11359_NBF_Ovaries | 0.0 |
| 12hr BF ovaries_11511_FPKM | 0.0 |
| 11512_24hr_BF_Ovaries | 0.0 |
| 11513_36hr_BF_Ovaries | 2.79902e-07 |
| 11514_48hr_BF_ovaries | 8274.9 |
| 11515_60hr_BF_ovaries | 11889.0 |
| 11360_72hr_BF_Ovaries | 175.811 |
| 11362_0-2Hr_Embryos | 0.0 |
| 11507_2-4hr_Embryos | 0.0 |
| 11508_4-8hr_Embryos | 0.0 |
| 11509_8-12hr_Embryos | 0.0 |
| 11724_12-16hr_embryos | 0.0 |
| 11772_16-20hr_Embryos | 0.266805 |
| 11773_20-24hr_embryos | 0.0538728 |
| 12105_24-28hr_Embryos | 0.0 |
| 12170_28-32hr_embryos | 0.0 |
| 12171_32-36hr_embryos | 0.0 |
| 11774_36-40hr_embryos | 0.0 |
| 11775_40-44hr_embryos | 0.0 |
| 11776_44-48hr_embryos | 0.141261 |
| 12172_48-52hr_embryos | 0.0 |
| 12173_52-56hr_embryos | 0.0 |
| 12106_56-60hr_Embryos | 0.0 |
| 11777_60-64hr_embryos | 0.0 |
| 12174_64-68hr_embryos | 0.0 |
| 12175_68-72hr_embryos | 0.0 |
| 12107_72-76hr_embryos | 0.45679 |
| 11782_1st_Instar_larvae | 0.418736 |
| 11783_2nd_Instar_larvae | 0.0 |
| 11784_3rd_Instar_Larvae | 0.0 |
| 11363_4th_instar_larvae | 0.0 |
| 11725_MalePupae | 0.0 |
| 11666_FemalePupae | 0.0 |Cysteine Rich Proteins

## Slide 19
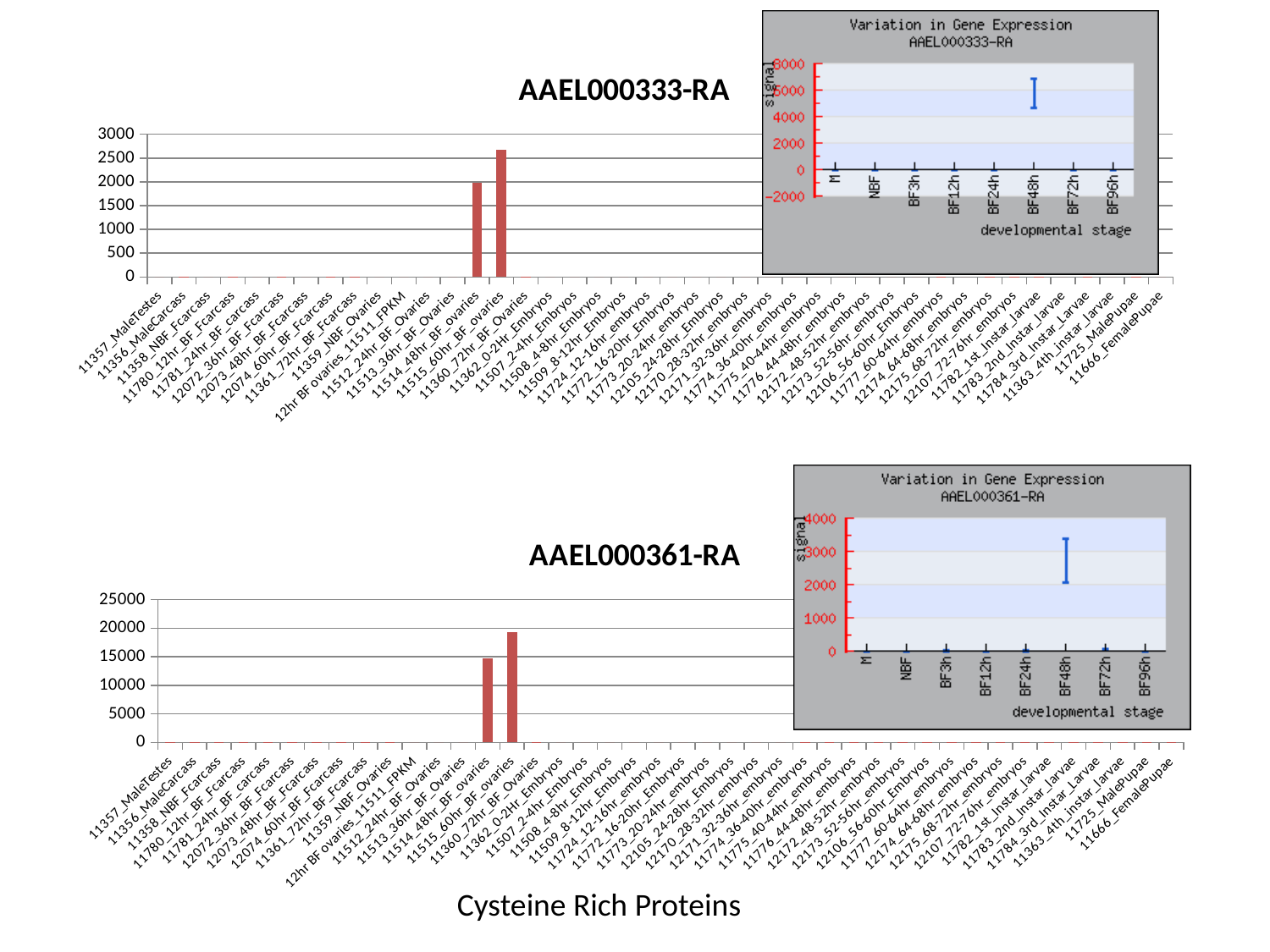

### Chart:
| Category | AAEL000333-RA |
|---|---|
| 11357_MaleTestes | 0.0 |
| 11356_MaleCarcass | 0.157999 |
| 11358_NBF_Fcarcass | 0.0 |
| 11780_12hr_BF_Fcarcass | 0.2966 |
| 11781_24hr_BF_carcass | 0.0 |
| 12072_36hr_BF_Fcarcass | 0.0141192 |
| 12073_48hr_BF_Fcarcass | 2.94218e-42 |
| 12074_60hr_BF_Fcarcass | 6.38605 |
| 11361_72hr_BF_Fcarcass | 0.0298747 |
| 11359_NBF_Ovaries | 0.0 |
| 12hr BF ovaries_11511_FPKM | 0.0 |
| 11512_24hr_BF_Ovaries | 0.0 |
| 11513_36hr_BF_Ovaries | 0.0 |
| 11514_48hr_BF_ovaries | 1977.22 |
| 11515_60hr_BF_ovaries | 2668.27 |
| 11360_72hr_BF_Ovaries | 0.116451 |
| 11362_0-2Hr_Embryos | 0.0 |
| 11507_2-4hr_Embryos | 0.0 |
| 11508_4-8hr_Embryos | 0.0 |
| 11509_8-12hr_Embryos | 0.0 |
| 11724_12-16hr_embryos | 0.0 |
| 11772_16-20hr_Embryos | 0.0 |
| 11773_20-24hr_embryos | 0.0 |
| 12105_24-28hr_Embryos | 0.0 |
| 12170_28-32hr_embryos | 0.0 |
| 12171_32-36hr_embryos | 0.0 |
| 11774_36-40hr_embryos | 0.0 |
| 11775_40-44hr_embryos | 0.0 |
| 11776_44-48hr_embryos | 0.0 |
| 12172_48-52hr_embryos | 0.0 |
| 12173_52-56hr_embryos | 0.0 |
| 12106_56-60hr_Embryos | 0.0 |
| 11777_60-64hr_embryos | 0.130326 |
| 12174_64-68hr_embryos | 0.0 |
| 12175_68-72hr_embryos | 0.101507 |
| 12107_72-76hr_embryos | 0.146038 |
| 11782_1st_Instar_larvae | 0.267744 |
| 11783_2nd_Instar_larvae | 0.0 |
| 11784_3rd_Instar_Larvae | 0.0584402 |
| 11363_4th_instar_larvae | 0.0 |
| 11725_MalePupae | 0.101507 |
| 11666_FemalePupae | 0.0 |
### Chart:
| Category | AAEL000361-RA |
|---|---|
| 11357_MaleTestes | 14.8082 |
| 11356_MaleCarcass | 2.99983 |
| 11358_NBF_Fcarcass | 5.22973 |
| 11780_12hr_BF_Fcarcass | 0.338092 |
| 11781_24hr_BF_carcass | 1.45761 |
| 12072_36hr_BF_Fcarcass | 3.62123 |
| 12073_48hr_BF_Fcarcass | 5.59549 |
| 12074_60hr_BF_Fcarcass | 38.9978 |
| 11361_72hr_BF_Fcarcass | 6.74058 |
| 11359_NBF_Ovaries | 0.642055 |
| 12hr BF ovaries_11511_FPKM | 0.0 |
| 11512_24hr_BF_Ovaries | 0.0 |
| 11513_36hr_BF_Ovaries | 0.0 |
| 11514_48hr_BF_ovaries | 14690.1 |
| 11515_60hr_BF_ovaries | 19370.1 |
| 11360_72hr_BF_Ovaries | 60.5781 |
| 11362_0-2Hr_Embryos | 0.0 |
| 11507_2-4hr_Embryos | 0.0 |
| 11508_4-8hr_Embryos | 0.0 |
| 11509_8-12hr_Embryos | 0.0 |
| 11724_12-16hr_embryos | 0.0 |
| 11772_16-20hr_Embryos | 0.0 |
| 11773_20-24hr_embryos | 0.0 |
| 12105_24-28hr_Embryos | 0.0 |
| 12170_28-32hr_embryos | 0.0 |
| 12171_32-36hr_embryos | 0.0 |
| 11774_36-40hr_embryos | 1.5783 |
| 11775_40-44hr_embryos | 2.8319 |
| 11776_44-48hr_embryos | 1.71516 |
| 12172_48-52hr_embryos | 2.31107 |
| 12173_52-56hr_embryos | 3.32835 |
| 12106_56-60hr_Embryos | 6.17582 |
| 11777_60-64hr_embryos | 8.91348 |
| 12174_64-68hr_embryos | 5.20096 |
| 12175_68-72hr_embryos | 6.82698 |
| 12107_72-76hr_embryos | 0.231061 |
| 11782_1st_Instar_larvae | 0.762523 |
| 11783_2nd_Instar_larvae | 5.70666 |
| 11784_3rd_Instar_Larvae | 6.99029 |
| 11363_4th_instar_larvae | 6.89456 |
| 11725_MalePupae | 6.82698 |
| 11666_FemalePupae | 0.950049 |Cysteine Rich Proteins

## Slide 20
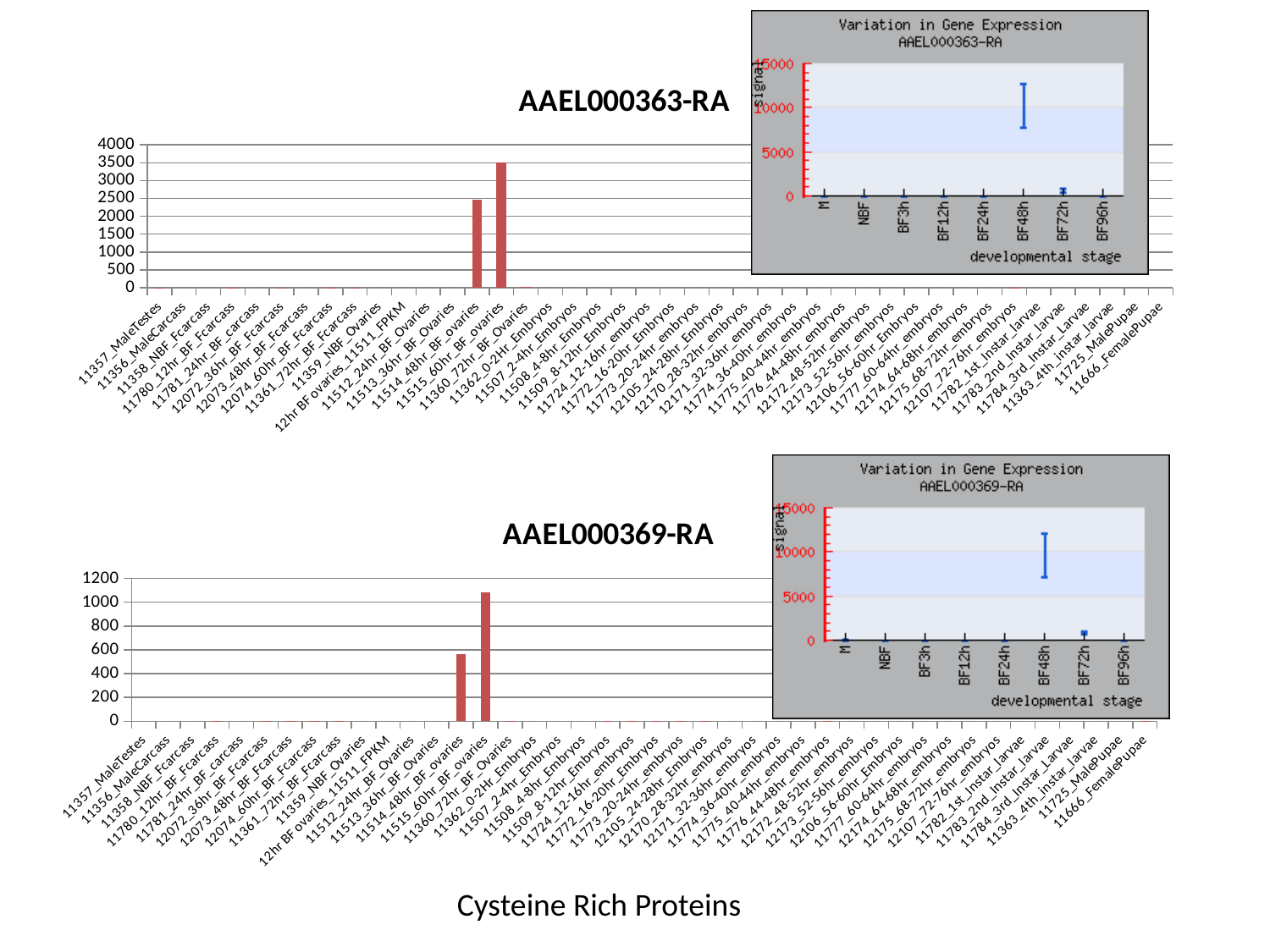

### Chart:
| Category | AAEL000363-RA |
|---|---|
| 11357_MaleTestes | 5.85059 |
| 11356_MaleCarcass | 0.0 |
| 11358_NBF_Fcarcass | 0.0 |
| 11780_12hr_BF_Fcarcass | 0.133577 |
| 11781_24hr_BF_carcass | 0.0 |
| 12072_36hr_BF_Fcarcass | 0.158969 |
| 12073_48hr_BF_Fcarcass | 0.0 |
| 12074_60hr_BF_Fcarcass | 7.9922 |
| 11361_72hr_BF_Fcarcass | 1.33157 |
| 11359_NBF_Ovaries | 0.0 |
| 12hr BF ovaries_11511_FPKM | 0.0 |
| 11512_24hr_BF_Ovaries | 0.0 |
| 11513_36hr_BF_Ovaries | 0.0 |
| 11514_48hr_BF_ovaries | 2465.5 |
| 11515_60hr_BF_ovaries | 3517.79 |
| 11360_72hr_BF_Ovaries | 26.3886 |
| 11362_0-2Hr_Embryos | 0.0 |
| 11507_2-4hr_Embryos | 0.0 |
| 11508_4-8hr_Embryos | 0.0 |
| 11509_8-12hr_Embryos | 0.0 |
| 11724_12-16hr_embryos | 0.0 |
| 11772_16-20hr_Embryos | 0.0 |
| 11773_20-24hr_embryos | 0.0 |
| 12105_24-28hr_Embryos | 0.0 |
| 12170_28-32hr_embryos | 0.0 |
| 12171_32-36hr_embryos | 0.0 |
| 11774_36-40hr_embryos | 0.0 |
| 11775_40-44hr_embryos | 0.0 |
| 11776_44-48hr_embryos | 0.0 |
| 12172_48-52hr_embryos | 0.0 |
| 12173_52-56hr_embryos | 0.0 |
| 12106_56-60hr_Embryos | 0.0 |
| 11777_60-64hr_embryos | 0.0 |
| 12174_64-68hr_embryos | 0.0 |
| 12175_68-72hr_embryos | 0.0 |
| 12107_72-76hr_embryos | 0.0913319 |
| 11782_1st_Instar_larvae | 0.0 |
| 11783_2nd_Instar_larvae | 0.0 |
| 11784_3rd_Instar_Larvae | 0.0 |
| 11363_4th_instar_larvae | 0.0 |
| 11725_MalePupae | 0.0 |
| 11666_FemalePupae | 0.0 |
### Chart:
| Category | AAEL000369-RA |
|---|---|
| 11357_MaleTestes | 0.0 |
| 11356_MaleCarcass | 0.0 |
| 11358_NBF_Fcarcass | 0.0 |
| 11780_12hr_BF_Fcarcass | 0.30278 |
| 11781_24hr_BF_carcass | 0.0 |
| 12072_36hr_BF_Fcarcass | 0.108101 |
| 12073_48hr_BF_Fcarcass | 0.0729598 |
| 12074_60hr_BF_Fcarcass | 1.3326 |
| 11361_72hr_BF_Fcarcass | 7.49399e-08 |
| 11359_NBF_Ovaries | 0.0 |
| 12hr BF ovaries_11511_FPKM | 0.0 |
| 11512_24hr_BF_Ovaries | 0.0 |
| 11513_36hr_BF_Ovaries | 0.0 |
| 11514_48hr_BF_ovaries | 567.482 |
| 11515_60hr_BF_ovaries | 1086.99 |
| 11360_72hr_BF_Ovaries | 1.76191 |
| 11362_0-2Hr_Embryos | 0.0 |
| 11507_2-4hr_Embryos | 0.0 |
| 11508_4-8hr_Embryos | 0.0 |
| 11509_8-12hr_Embryos | 0.758265 |
| 11724_12-16hr_embryos | 0.133021 |
| 11772_16-20hr_Embryos | 0.261101 |
| 11773_20-24hr_embryos | 7.40773e-07 |
| 12105_24-28hr_Embryos | 0.0839075 |
| 12170_28-32hr_embryos | 0.0 |
| 12171_32-36hr_embryos | 0.0 |
| 11774_36-40hr_embryos | 0.0 |
| 11775_40-44hr_embryos | 0.0 |
| 11776_44-48hr_embryos | 0.0460793 |
| 12172_48-52hr_embryos | 0.0 |
| 12173_52-56hr_embryos | 0.0 |
| 12106_56-60hr_Embryos | 0.0 |
| 11777_60-64hr_embryos | 0.0 |
| 12174_64-68hr_embryos | 0.0 |
| 12175_68-72hr_embryos | 0.0 |
| 12107_72-76hr_embryos | 0.0 |
| 11782_1st_Instar_larvae | 0.0 |
| 11783_2nd_Instar_larvae | 0.0 |
| 11784_3rd_Instar_Larvae | 0.0 |
| 11363_4th_instar_larvae | 0.0 |
| 11725_MalePupae | 0.0 |
| 11666_FemalePupae | 0.127623 |Cysteine Rich Proteins

## Slide 21
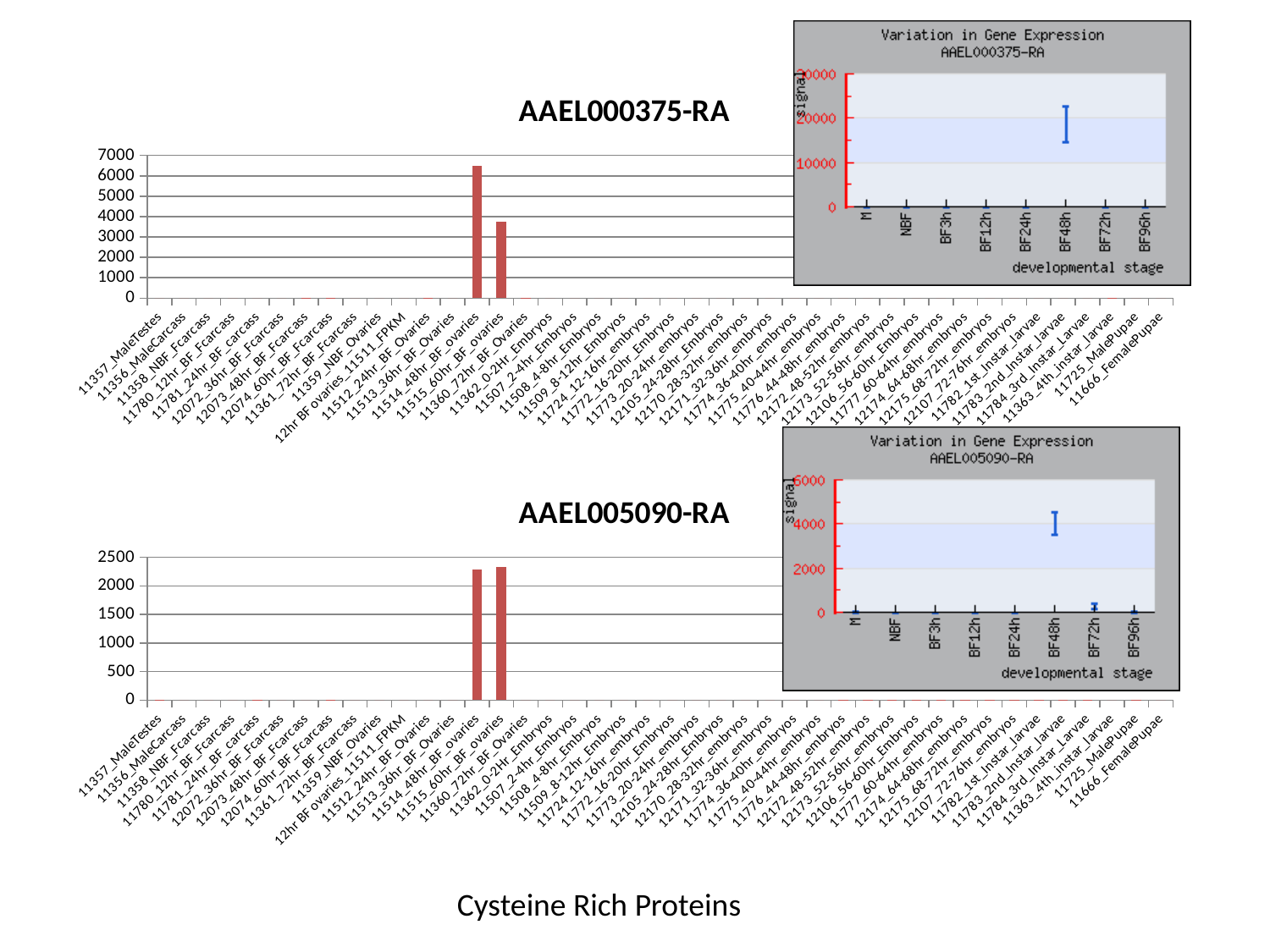

### Chart:
| Category | AAEL000375-RA |
|---|---|
| 11357_MaleTestes | 0.0 |
| 11356_MaleCarcass | 0.0 |
| 11358_NBF_Fcarcass | 0.0 |
| 11780_12hr_BF_Fcarcass | 0.0 |
| 11781_24hr_BF_carcass | 0.0 |
| 12072_36hr_BF_Fcarcass | 0.0 |
| 12073_48hr_BF_Fcarcass | 3.98195 |
| 12074_60hr_BF_Fcarcass | 5.2209 |
| 11361_72hr_BF_Fcarcass | 0.0 |
| 11359_NBF_Ovaries | 0.0 |
| 12hr BF ovaries_11511_FPKM | 0.0 |
| 11512_24hr_BF_Ovaries | 0.231502 |
| 11513_36hr_BF_Ovaries | 0.0 |
| 11514_48hr_BF_ovaries | 6506.32 |
| 11515_60hr_BF_ovaries | 3740.92 |
| 11360_72hr_BF_Ovaries | 2.65378 |
| 11362_0-2Hr_Embryos | 0.0 |
| 11507_2-4hr_Embryos | 0.0 |
| 11508_4-8hr_Embryos | 0.0 |
| 11509_8-12hr_Embryos | 0.0 |
| 11724_12-16hr_embryos | 0.0 |
| 11772_16-20hr_Embryos | 0.0 |
| 11773_20-24hr_embryos | 0.0 |
| 12105_24-28hr_Embryos | 0.0 |
| 12170_28-32hr_embryos | 0.0 |
| 12171_32-36hr_embryos | 0.0 |
| 11774_36-40hr_embryos | 0.0 |
| 11775_40-44hr_embryos | 0.0 |
| 11776_44-48hr_embryos | 0.0 |
| 12172_48-52hr_embryos | 0.0 |
| 12173_52-56hr_embryos | 0.0 |
| 12106_56-60hr_Embryos | 0.0 |
| 11777_60-64hr_embryos | 0.0 |
| 12174_64-68hr_embryos | 0.0 |
| 12175_68-72hr_embryos | 0.0 |
| 12107_72-76hr_embryos | 0.0 |
| 11782_1st_Instar_larvae | 0.0 |
| 11783_2nd_Instar_larvae | 0.0 |
| 11784_3rd_Instar_Larvae | 0.0 |
| 11363_4th_instar_larvae | 1.89327 |
| 11725_MalePupae | 0.0 |
| 11666_FemalePupae | 0.0 |
### Chart:
| Category | AAEL005090-RA |
|---|---|
| 11357_MaleTestes | 0.740312 |
| 11356_MaleCarcass | 0.0 |
| 11358_NBF_Fcarcass | 0.0 |
| 11780_12hr_BF_Fcarcass | 0.0 |
| 11781_24hr_BF_carcass | 0.095643 |
| 12072_36hr_BF_Fcarcass | 0.0 |
| 12073_48hr_BF_Fcarcass | 0.0 |
| 12074_60hr_BF_Fcarcass | 4.18821 |
| 11361_72hr_BF_Fcarcass | 0.0 |
| 11359_NBF_Ovaries | 0.0 |
| 12hr BF ovaries_11511_FPKM | 0.0 |
| 11512_24hr_BF_Ovaries | 0.0 |
| 11513_36hr_BF_Ovaries | 0.0 |
| 11514_48hr_BF_ovaries | 2288.63 |
| 11515_60hr_BF_ovaries | 2334.99 |
| 11360_72hr_BF_Ovaries | 0.0 |
| 11362_0-2Hr_Embryos | 0.0 |
| 11507_2-4hr_Embryos | 0.0 |
| 11508_4-8hr_Embryos | 0.0 |
| 11509_8-12hr_Embryos | 0.0 |
| 11724_12-16hr_embryos | 0.0 |
| 11772_16-20hr_Embryos | 0.0 |
| 11773_20-24hr_embryos | 0.0 |
| 12105_24-28hr_Embryos | 0.0 |
| 12170_28-32hr_embryos | 0.0 |
| 12171_32-36hr_embryos | 0.0 |
| 11774_36-40hr_embryos | 0.0 |
| 11775_40-44hr_embryos | 0.0 |
| 11776_44-48hr_embryos | 0.180068 |
| 12172_48-52hr_embryos | 0.390349 |
| 12173_52-56hr_embryos | 1.0574 |
| 12106_56-60hr_Embryos | 3.24187 |
| 11777_60-64hr_embryos | 5.46058 |
| 12174_64-68hr_embryos | 1.4631 |
| 12175_68-72hr_embryos | 1.02391 |
| 12107_72-76hr_embryos | 0.121334 |
| 11782_1st_Instar_larvae | 0.625647 |
| 11783_2nd_Instar_larvae | 0.187292 |
| 11784_3rd_Instar_Larvae | 0.087398 |
| 11363_4th_instar_larvae | 0.0 |
| 11725_MalePupae | 1.02391 |
| 11666_FemalePupae | 0.0 |Cysteine Rich Proteins

## Slide 22
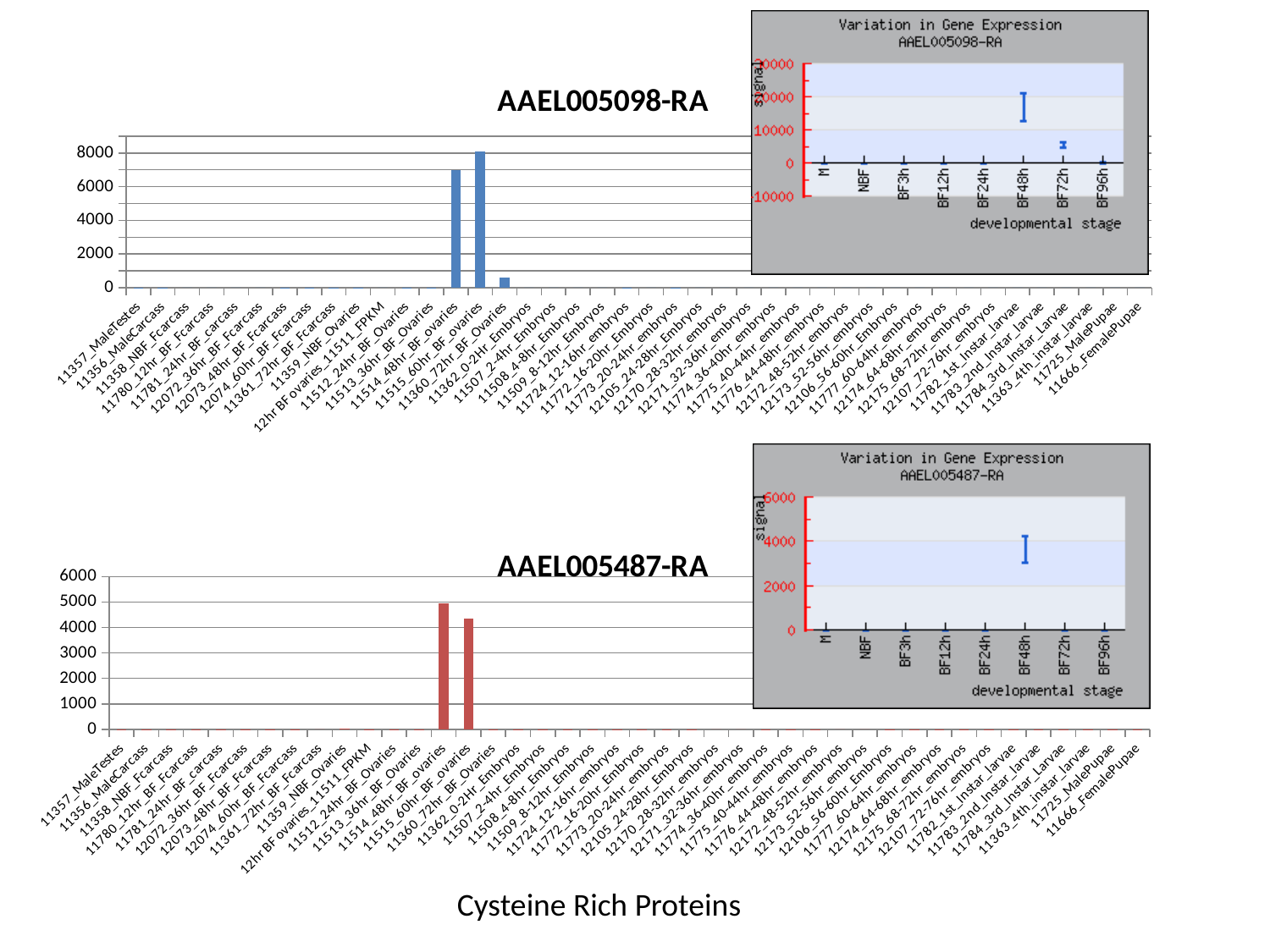

### Chart:
| Category | AAEL005098-RA |
|---|---|
| 11357_MaleTestes | 6.24841 |
| 11356_MaleCarcass | 0.220525 |
| 11358_NBF_Fcarcass | 0.0 |
| 11780_12hr_BF_Fcarcass | 0.0 |
| 11781_24hr_BF_carcass | 0.0 |
| 12072_36hr_BF_Fcarcass | 0.0 |
| 12073_48hr_BF_Fcarcass | 0.800328 |
| 12074_60hr_BF_Fcarcass | 10.9919 |
| 11361_72hr_BF_Fcarcass | 14.6528 |
| 11359_NBF_Ovaries | 1.86767e-06 |
| 12hr BF ovaries_11511_FPKM | 0.0 |
| 11512_24hr_BF_Ovaries | 0.273403 |
| 11513_36hr_BF_Ovaries | 0.891558 |
| 11514_48hr_BF_ovaries | 6968.33 |
| 11515_60hr_BF_ovaries | 8108.35 |
| 11360_72hr_BF_Ovaries | 596.716 |
| 11362_0-2Hr_Embryos | 0.0 |
| 11507_2-4hr_Embryos | 0.0 |
| 11508_4-8hr_Embryos | 0.0 |
| 11509_8-12hr_Embryos | 0.0 |
| 11724_12-16hr_embryos | 0.181899 |
| 11772_16-20hr_Embryos | 0.0 |
| 11773_20-24hr_embryos | 0.109986 |
| 12105_24-28hr_Embryos | 0.0 |
| 12170_28-32hr_embryos | 0.0 |
| 12171_32-36hr_embryos | 0.0 |
| 11774_36-40hr_embryos | 0.0 |
| 11775_40-44hr_embryos | 0.0 |
| 11776_44-48hr_embryos | 0.0 |
| 12172_48-52hr_embryos | 0.0 |
| 12173_52-56hr_embryos | 0.0 |
| 12106_56-60hr_Embryos | 0.0 |
| 11777_60-64hr_embryos | 0.0 |
| 12174_64-68hr_embryos | 0.0 |
| 12175_68-72hr_embryos | 0.0 |
| 12107_72-76hr_embryos | 0.0 |
| 11782_1st_Instar_larvae | 0.0 |
| 11783_2nd_Instar_larvae | 0.0 |
| 11784_3rd_Instar_Larvae | 0.0 |
| 11363_4th_instar_larvae | 0.0 |
| 11725_MalePupae | 0.0 |
| 11666_FemalePupae | 0.0 |
### Chart:
| Category | AAEL005487-RA |
|---|---|
| 11357_MaleTestes | 0.597559 |
| 11356_MaleCarcass | 0.494331 |
| 11358_NBF_Fcarcass | 0.876039 |
| 11780_12hr_BF_Fcarcass | 0.679611 |
| 11781_24hr_BF_carcass | 0.137344 |
| 12072_36hr_BF_Fcarcass | 0.407504 |
| 12073_48hr_BF_Fcarcass | 0.58977 |
| 12074_60hr_BF_Fcarcass | 9.76656 |
| 11361_72hr_BF_Fcarcass | 0.0 |
| 11359_NBF_Ovaries | 18.512 |
| 12hr BF ovaries_11511_FPKM | 5.1123 |
| 11512_24hr_BF_Ovaries | 2.72766 |
| 11513_36hr_BF_Ovaries | 4.22353 |
| 11514_48hr_BF_ovaries | 4949.19 |
| 11515_60hr_BF_ovaries | 4350.2 |
| 11360_72hr_BF_Ovaries | 2.88264 |
| 11362_0-2Hr_Embryos | 0.893236 |
| 11507_2-4hr_Embryos | 2.79493 |
| 11508_4-8hr_Embryos | 1.93311 |
| 11509_8-12hr_Embryos | 1.38106 |
| 11724_12-16hr_embryos | 1.52398 |
| 11772_16-20hr_Embryos | 1.55836 |
| 11773_20-24hr_embryos | 1.32707 |
| 12105_24-28hr_Embryos | 0.961222 |
| 12170_28-32hr_embryos | 0.0 |
| 12171_32-36hr_embryos | 0.0 |
| 11774_36-40hr_embryos | 0.634519 |
| 11775_40-44hr_embryos | 0.184129 |
| 11776_44-48hr_embryos | 1.05586 |
| 12172_48-52hr_embryos | 0.0 |
| 12173_52-56hr_embryos | 0.0 |
| 12106_56-60hr_Embryos | 0.116383 |
| 11777_60-64hr_embryos | 4.17993 |
| 12174_64-68hr_embryos | 3.14381 |
| 12175_68-72hr_embryos | 2.88253 |
| 12107_72-76hr_embryos | 0.348513 |
| 11782_1st_Instar_larvae | 0.575061 |
| 11783_2nd_Instar_larvae | 1.22963 |
| 11784_3rd_Instar_Larvae | 0.784489 |
| 11363_4th_instar_larvae | 2.41866 |
| 11725_MalePupae | 2.88253 |
| 11666_FemalePupae | 3.25317 |Cysteine Rich Proteins

## Slide 23
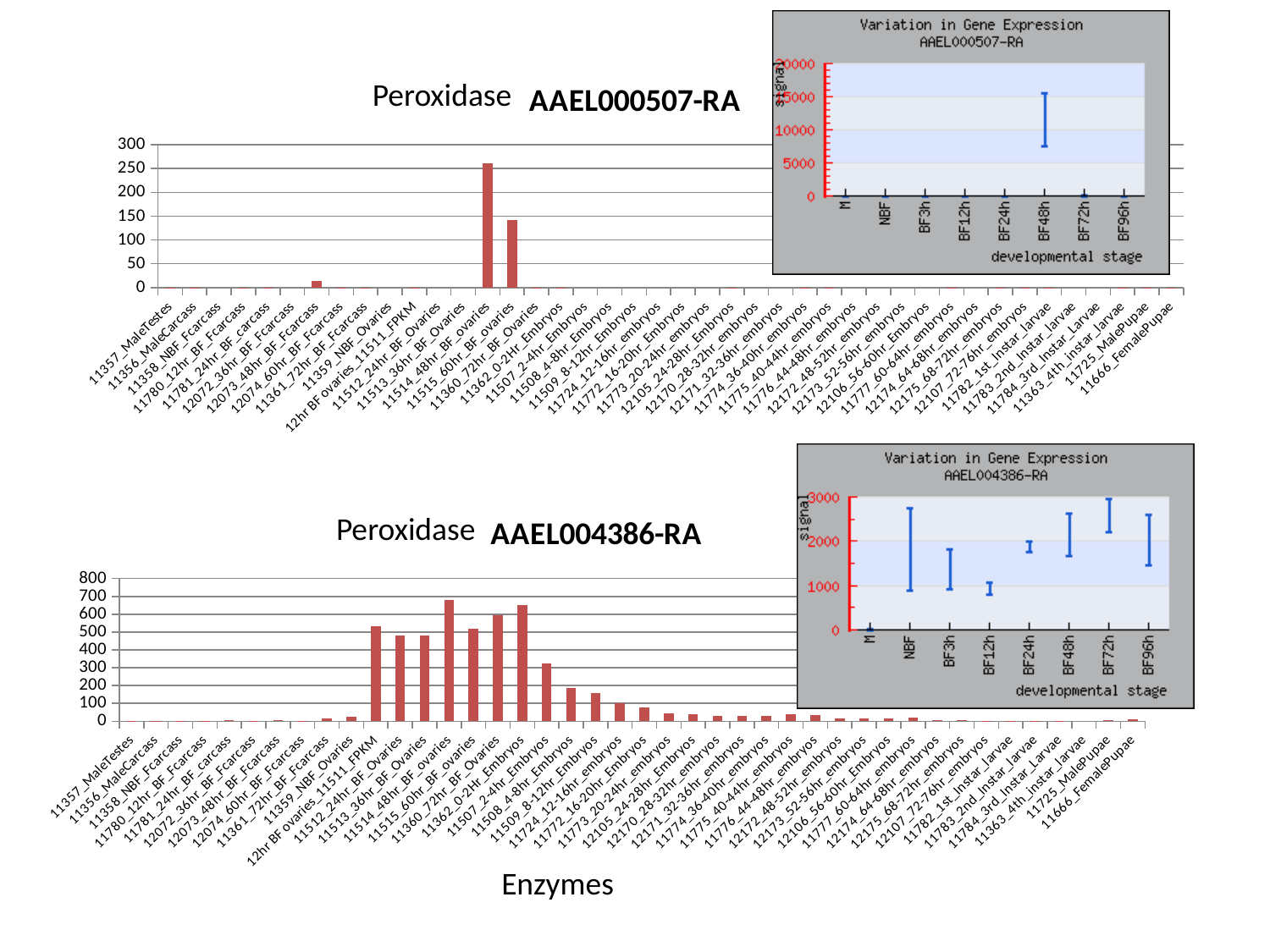

### Chart:
| Category | AAEL000507-RA |
|---|---|
| 11357_MaleTestes | 2.11304e-06 |
| 11356_MaleCarcass | 2.26084e-06 |
| 11358_NBF_Fcarcass | 0.0 |
| 11780_12hr_BF_Fcarcass | 1.01874e-06 |
| 11781_24hr_BF_carcass | 9.62475e-07 |
| 12072_36hr_BF_Fcarcass | 0.0 |
| 12073_48hr_BF_Fcarcass | 14.5755 |
| 12074_60hr_BF_Fcarcass | 0.069609 |
| 11361_72hr_BF_Fcarcass | 2.14833e-06 |
| 11359_NBF_Ovaries | 0.0 |
| 12hr BF ovaries_11511_FPKM | 2.71231e-06 |
| 11512_24hr_BF_Ovaries | 0.0 |
| 11513_36hr_BF_Ovaries | 0.0 |
| 11514_48hr_BF_ovaries | 261.764 |
| 11515_60hr_BF_ovaries | 141.175 |
| 11360_72hr_BF_Ovaries | 1.29445e-06 |
| 11362_0-2Hr_Embryos | 1.87677e-06 |
| 11507_2-4hr_Embryos | 0.0 |
| 11508_4-8hr_Embryos | 0.0 |
| 11509_8-12hr_Embryos | 0.0 |
| 11724_12-16hr_embryos | 0.0 |
| 11772_16-20hr_Embryos | 0.0 |
| 11773_20-24hr_embryos | 0.0 |
| 12105_24-28hr_Embryos | 9.36009e-07 |
| 12170_28-32hr_embryos | 0.0 |
| 12171_32-36hr_embryos | 0.0 |
| 11774_36-40hr_embryos | 1.61647e-06 |
| 11775_40-44hr_embryos | 9.42883e-07 |
| 11776_44-48hr_embryos | 0.0 |
| 12172_48-52hr_embryos | 0.0 |
| 12173_52-56hr_embryos | 0.0 |
| 12106_56-60hr_Embryos | 0.0 |
| 11777_60-64hr_embryos | 9.80942e-07 |
| 12174_64-68hr_embryos | 0.0 |
| 12175_68-72hr_embryos | 1.66479e-06 |
| 12107_72-76hr_embryos | 6.10722e-07 |
| 11782_1st_Instar_larvae | 1.14329e-06 |
| 11783_2nd_Instar_larvae | 0.0 |
| 11784_3rd_Instar_Larvae | 0.0 |
| 11363_4th_instar_larvae | 1.58923e-06 |
| 11725_MalePupae | 1.66479e-06 |
| 11666_FemalePupae | 1.42344e-06 |Peroxidase
### Chart:
| Category | AAEL004386-RA |
|---|---|
| 11357_MaleTestes | 2.36241 |
| 11356_MaleCarcass | 0.643688 |
| 11358_NBF_Fcarcass | 1.08389 |
| 11780_12hr_BF_Fcarcass | 0.274242 |
| 11781_24hr_BF_carcass | 5.43067 |
| 12072_36hr_BF_Fcarcass | 0.834258 |
| 12073_48hr_BF_Fcarcass | 6.34501 |
| 12074_60hr_BF_Fcarcass | 1.9722 |
| 11361_72hr_BF_Fcarcass | 14.8465 |
| 11359_NBF_Ovaries | 24.4781 |
| 12hr BF ovaries_11511_FPKM | 534.303 |
| 11512_24hr_BF_Ovaries | 480.745 |
| 11513_36hr_BF_Ovaries | 478.772 |
| 11514_48hr_BF_ovaries | 677.865 |
| 11515_60hr_BF_ovaries | 518.097 |
| 11360_72hr_BF_Ovaries | 596.275 |
| 11362_0-2Hr_Embryos | 652.603 |
| 11507_2-4hr_Embryos | 324.062 |
| 11508_4-8hr_Embryos | 188.298 |
| 11509_8-12hr_Embryos | 160.146 |
| 11724_12-16hr_embryos | 100.983 |
| 11772_16-20hr_Embryos | 75.7648 |
| 11773_20-24hr_embryos | 44.0024 |
| 12105_24-28hr_Embryos | 38.2656 |
| 12170_28-32hr_embryos | 29.3914 |
| 12171_32-36hr_embryos | 28.8246 |
| 11774_36-40hr_embryos | 32.1244 |
| 11775_40-44hr_embryos | 40.7324 |
| 11776_44-48hr_embryos | 36.229 |
| 12172_48-52hr_embryos | 17.9281 |
| 12173_52-56hr_embryos | 16.1491 |
| 12106_56-60hr_Embryos | 15.0124 |
| 11777_60-64hr_embryos | 21.5919 |
| 12174_64-68hr_embryos | 6.89928 |
| 12175_68-72hr_embryos | 5.26062 |
| 12107_72-76hr_embryos | 0.0289802 |
| 11782_1st_Instar_larvae | 0.907498 |
| 11783_2nd_Instar_larvae | 0.0215385 |
| 11784_3rd_Instar_Larvae | 0.0201015 |
| 11363_4th_instar_larvae | 0.0 |
| 11725_MalePupae | 5.26062 |
| 11666_FemalePupae | 9.4023 |Peroxidase
Enzymes

## Slide 24
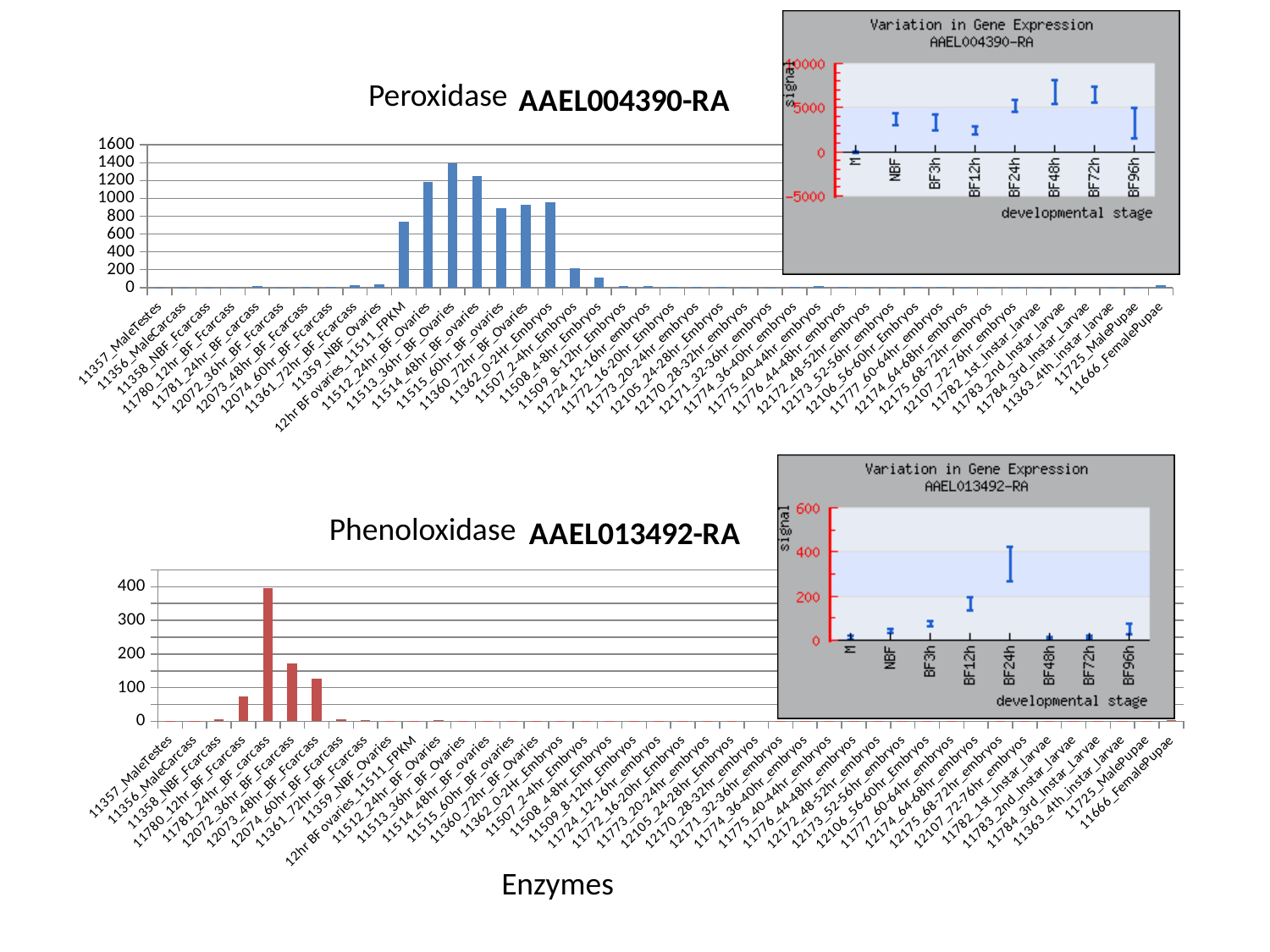

### Chart:
| Category | AAEL004390-RA |
|---|---|
| 11357_MaleTestes | 2.08828 |
| 11356_MaleCarcass | 0.2413 |
| 11358_NBF_Fcarcass | 1.70851 |
| 11780_12hr_BF_Fcarcass | 0.299564 |
| 11781_24hr_BF_carcass | 14.4266 |
| 12072_36hr_BF_Fcarcass | 1.87074 |
| 12073_48hr_BF_Fcarcass | 10.8417 |
| 12074_60hr_BF_Fcarcass | 3.47801 |
| 11361_72hr_BF_Fcarcass | 24.1888 |
| 11359_NBF_Ovaries | 34.8034 |
| 12hr BF ovaries_11511_FPKM | 734.896 |
| 11512_24hr_BF_Ovaries | 1181.17 |
| 11513_36hr_BF_Ovaries | 1392.79 |
| 11514_48hr_BF_ovaries | 1251.83 |
| 11515_60hr_BF_ovaries | 885.977 |
| 11360_72hr_BF_Ovaries | 930.693 |
| 11362_0-2Hr_Embryos | 957.469 |
| 11507_2-4hr_Embryos | 216.548 |
| 11508_4-8hr_Embryos | 107.657 |
| 11509_8-12hr_Embryos | 19.9599 |
| 11724_12-16hr_embryos | 15.4898 |
| 11772_16-20hr_Embryos | 10.8095 |
| 11773_20-24hr_embryos | 5.88138 |
| 12105_24-28hr_Embryos | 5.76959 |
| 12170_28-32hr_embryos | 2.79846 |
| 12171_32-36hr_embryos | 2.93288 |
| 11774_36-40hr_embryos | 8.49898 |
| 11775_40-44hr_embryos | 13.8621 |
| 11776_44-48hr_embryos | 12.3221 |
| 12172_48-52hr_embryos | 2.99412 |
| 12173_52-56hr_embryos | 2.2232 |
| 12106_56-60hr_Embryos | 4.98381 |
| 11777_60-64hr_embryos | 8.9847 |
| 12174_64-68hr_embryos | 0.974256 |
| 12175_68-72hr_embryos | 2.03273 |
| 12107_72-76hr_embryos | 0.167098 |
| 11782_1st_Instar_larvae | 0.61082 |
| 11783_2nd_Instar_larvae | 0.0215982 |
| 11784_3rd_Instar_Larvae | 0.0 |
| 11363_4th_instar_larvae | 0.144418 |
| 11725_MalePupae | 2.03273 |
| 11666_FemalePupae | 24.0504 |Peroxidase
### Chart:
| Category | AAEL013492-RA |
|---|---|
| 11357_MaleTestes | 0.348436 |
| 11356_MaleCarcass | 0.63572 |
| 11358_NBF_Fcarcass | 5.66054 |
| 11780_12hr_BF_Fcarcass | 72.6953 |
| 11781_24hr_BF_carcass | 395.776 |
| 12072_36hr_BF_Fcarcass | 171.04 |
| 12073_48hr_BF_Fcarcass | 127.545 |
| 12074_60hr_BF_Fcarcass | 4.84935 |
| 11361_72hr_BF_Fcarcass | 3.0928 |
| 11359_NBF_Ovaries | 0.135968 |
| 12hr BF ovaries_11511_FPKM | 2.05634 |
| 11512_24hr_BF_Ovaries | 3.04792 |
| 11513_36hr_BF_Ovaries | 1.00236 |
| 11514_48hr_BF_ovaries | 0.502933 |
| 11515_60hr_BF_ovaries | 0.429851 |
| 11360_72hr_BF_Ovaries | 0.475133 |
| 11362_0-2Hr_Embryos | 0.574743 |
| 11507_2-4hr_Embryos | 0.474525 |
| 11508_4-8hr_Embryos | 0.43645 |
| 11509_8-12hr_Embryos | 0.191258 |
| 11724_12-16hr_embryos | 0.0349502 |
| 11772_16-20hr_Embryos | 0.137204 |
| 11773_20-24hr_embryos | 0.169063 |
| 12105_24-28hr_Embryos | 0.066148 |
| 12170_28-32hr_embryos | 0.0 |
| 12171_32-36hr_embryos | 0.0759405 |
| 11774_36-40hr_embryos | 0.289671 |
| 11775_40-44hr_embryos | 0.377594 |
| 11776_44-48hr_embryos | 0.508506 |
| 12172_48-52hr_embryos | 0.0467072 |
| 12173_52-56hr_embryos | 0.0940452 |
| 12106_56-60hr_Embryos | 0.0217975 |
| 11777_60-64hr_embryos | 0.209734 |
| 12174_64-68hr_embryos | 0.0 |
| 12175_68-72hr_embryos | 0.0229483 |
| 12107_72-76hr_embryos | 0.359085 |
| 11782_1st_Instar_larvae | 0.484778 |
| 11783_2nd_Instar_larvae | 0.100779 |
| 11784_3rd_Instar_Larvae | 0.141083 |
| 11363_4th_instar_larvae | 0.299499 |
| 11725_MalePupae | 0.0229483 |
| 11666_FemalePupae | 2.61549 |Phenoloxidase
Enzymes

## Slide 25
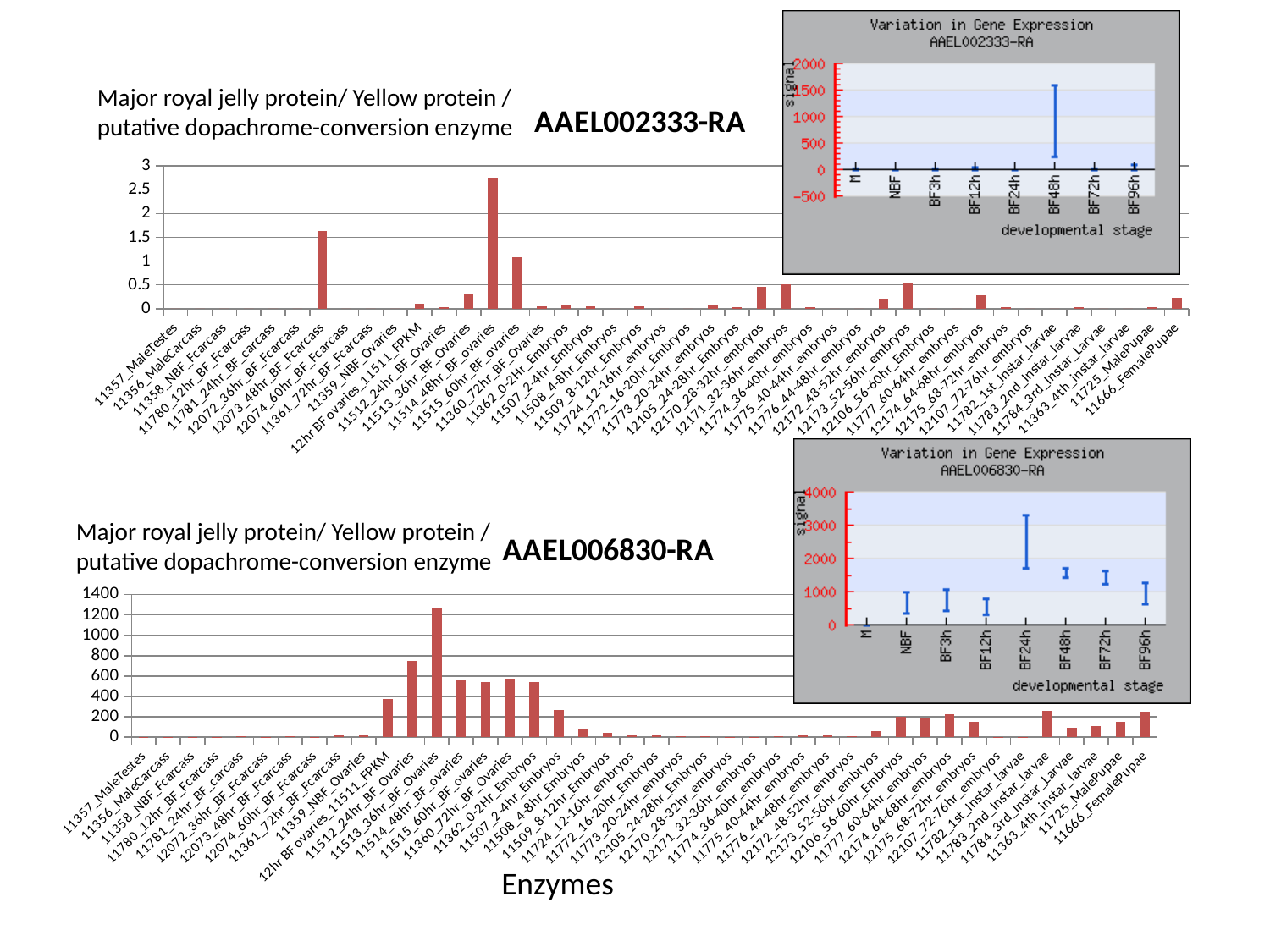

Major royal jelly protein/ Yellow protein / putative dopachrome-conversion enzyme
### Chart:
| Category | AAEL002333-RA |
|---|---|
| 11357_MaleTestes | 0.0 |
| 11356_MaleCarcass | 0.0 |
| 11358_NBF_Fcarcass | 0.0 |
| 11780_12hr_BF_Fcarcass | 0.0 |
| 11781_24hr_BF_carcass | 0.0 |
| 12072_36hr_BF_Fcarcass | 0.0 |
| 12073_48hr_BF_Fcarcass | 1.62699 |
| 12074_60hr_BF_Fcarcass | 0.0 |
| 11361_72hr_BF_Fcarcass | 0.0 |
| 11359_NBF_Ovaries | 0.0 |
| 12hr BF ovaries_11511_FPKM | 0.108309 |
| 11512_24hr_BF_Ovaries | 0.0339494 |
| 11513_36hr_BF_Ovaries | 0.297787 |
| 11514_48hr_BF_ovaries | 2.76024 |
| 11515_60hr_BF_ovaries | 1.08958 |
| 11360_72hr_BF_Ovaries | 0.0529388 |
| 11362_0-2Hr_Embryos | 0.0640373 |
| 11507_2-4hr_Embryos | 0.0458216 |
| 11508_4-8hr_Embryos | 0.0 |
| 11509_8-12hr_Embryos | 0.0461712 |
| 11724_12-16hr_embryos | 0.0 |
| 11772_16-20hr_Embryos | 0.0 |
| 11773_20-24hr_embryos | 0.0612196 |
| 12105_24-28hr_Embryos | 0.031934 |
| 12170_28-32hr_embryos | 0.460418 |
| 12171_32-36hr_embryos | 0.513261 |
| 11774_36-40hr_embryos | 0.0322748 |
| 11775_40-44hr_embryos | 0.0 |
| 11776_44-48hr_embryos | 0.0 |
| 12172_48-52hr_embryos | 0.211352 |
| 12173_52-56hr_embryos | 0.547254 |
| 12106_56-60hr_Embryos | 0.0 |
| 11777_60-64hr_embryos | 0.0 |
| 12174_64-68hr_embryos | 0.289696 |
| 12175_68-72hr_embryos | 0.0332394 |
| 12107_72-76hr_embryos | 0.0 |
| 11782_1st_Instar_larvae | 0.0 |
| 11783_2nd_Instar_larvae | 0.0364895 |
| 11784_3rd_Instar_Larvae | 0.0 |
| 11363_4th_instar_larvae | 0.0 |
| 11725_MalePupae | 0.0332394 |
| 11666_FemalePupae | 0.218561 |
Major royal jelly protein/ Yellow protein / putative dopachrome-conversion enzyme
### Chart:
| Category | AAEL006830-RA |
|---|---|
| 11357_MaleTestes | 3.75901 |
| 11356_MaleCarcass | 1.0373 |
| 11358_NBF_Fcarcass | 1.17796 |
| 11780_12hr_BF_Fcarcass | 0.482655 |
| 11781_24hr_BF_carcass | 5.58141 |
| 12072_36hr_BF_Fcarcass | 2.71887 |
| 12073_48hr_BF_Fcarcass | 6.37256 |
| 12074_60hr_BF_Fcarcass | 1.84063 |
| 11361_72hr_BF_Fcarcass | 18.0506 |
| 11359_NBF_Ovaries | 22.6088 |
| 12hr BF ovaries_11511_FPKM | 374.206 |
| 11512_24hr_BF_Ovaries | 746.241 |
| 11513_36hr_BF_Ovaries | 1259.76 |
| 11514_48hr_BF_ovaries | 558.577 |
| 11515_60hr_BF_ovaries | 540.183 |
| 11360_72hr_BF_Ovaries | 571.683 |
| 11362_0-2Hr_Embryos | 539.695 |
| 11507_2-4hr_Embryos | 268.813 |
| 11508_4-8hr_Embryos | 71.6447 |
| 11509_8-12hr_Embryos | 44.1437 |
| 11724_12-16hr_embryos | 21.9327 |
| 11772_16-20hr_Embryos | 18.3877 |
| 11773_20-24hr_embryos | 9.59789 |
| 12105_24-28hr_Embryos | 10.9053 |
| 12170_28-32hr_embryos | 3.77016 |
| 12171_32-36hr_embryos | 4.65786 |
| 11774_36-40hr_embryos | 7.46554 |
| 11775_40-44hr_embryos | 14.9544 |
| 11776_44-48hr_embryos | 13.47 |
| 12172_48-52hr_embryos | 5.96146 |
| 12173_52-56hr_embryos | 59.3921 |
| 12106_56-60hr_Embryos | 202.099 |
| 11777_60-64hr_embryos | 180.749 |
| 12174_64-68hr_embryos | 221.878 |
| 12175_68-72hr_embryos | 146.207 |
| 12107_72-76hr_embryos | 2.43051 |
| 11782_1st_Instar_larvae | 4.86981 |
| 11783_2nd_Instar_larvae | 254.9 |
| 11784_3rd_Instar_Larvae | 93.4369 |
| 11363_4th_instar_larvae | 106.397 |
| 11725_MalePupae | 146.207 |
| 11666_FemalePupae | 251.941 |Enzymes

## Slide 26
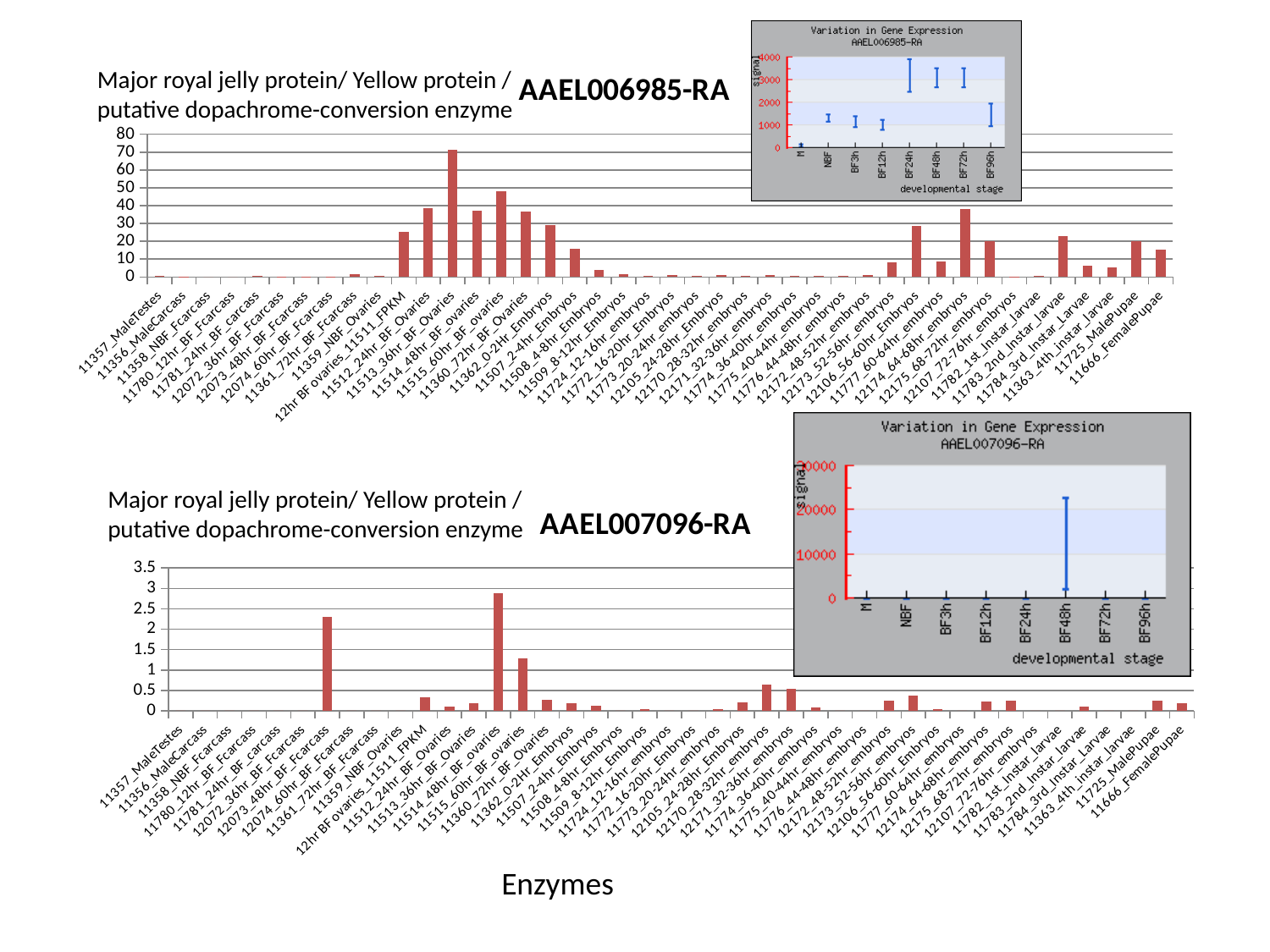

### Chart:
| Category | AAEL006985-RA |
|---|---|
| 11357_MaleTestes | 0.54527 |
| 11356_MaleCarcass | 0.374441 |
| 11358_NBF_Fcarcass | 0.0 |
| 11780_12hr_BF_Fcarcass | 0.0 |
| 11781_24hr_BF_carcass | 0.556603 |
| 12072_36hr_BF_Fcarcass | 0.250953 |
| 12073_48hr_BF_Fcarcass | 0.135035 |
| 12074_60hr_BF_Fcarcass | 0.177766 |
| 11361_72hr_BF_Fcarcass | 1.3742 |
| 11359_NBF_Ovaries | 0.490349 |
| 12hr BF ovaries_11511_FPKM | 25.2411 |
| 11512_24hr_BF_Ovaries | 38.7013 |
| 11513_36hr_BF_Ovaries | 71.2919 |
| 11514_48hr_BF_ovaries | 37.3948 |
| 11515_60hr_BF_ovaries | 47.9593 |
| 11360_72hr_BF_Ovaries | 36.4581 |
| 11362_0-2Hr_Embryos | 28.9864 |
| 11507_2-4hr_Embryos | 15.881 |
| 11508_4-8hr_Embryos | 3.84082 |
| 11509_8-12hr_Embryos | 1.79334 |
| 11724_12-16hr_embryos | 0.682997 |
| 11772_16-20hr_Embryos | 0.891382 |
| 11773_20-24hr_embryos | 0.777371 |
| 12105_24-28hr_Embryos | 1.33582 |
| 12170_28-32hr_embryos | 0.831121 |
| 12171_32-36hr_embryos | 1.0414 |
| 11774_36-40hr_embryos | 0.433935 |
| 11775_40-44hr_embryos | 0.768985 |
| 11776_44-48hr_embryos | 0.681148 |
| 12172_48-52hr_embryos | 1.01047 |
| 12173_52-56hr_embryos | 8.3938 |
| 12106_56-60hr_Embryos | 28.6297 |
| 11777_60-64hr_embryos | 8.90633 |
| 12174_64-68hr_embryos | 37.9973 |
| 12175_68-72hr_embryos | 19.9121 |
| 12107_72-76hr_embryos | 0.0794605 |
| 11782_1st_Instar_larvae | 0.410336 |
| 11783_2nd_Instar_larvae | 22.7321 |
| 11784_3rd_Instar_Larvae | 6.25791 |
| 11363_4th_instar_larvae | 5.50883 |
| 11725_MalePupae | 19.9121 |
| 11666_FemalePupae | 15.1645 |Major royal jelly protein/ Yellow protein / putative dopachrome-conversion enzyme
Major royal jelly protein/ Yellow protein / putative dopachrome-conversion enzyme
### Chart:
| Category | AAEL007096-RA |
|---|---|
| 11357_MaleTestes | 0.0 |
| 11356_MaleCarcass | 0.0 |
| 11358_NBF_Fcarcass | 0.0 |
| 11780_12hr_BF_Fcarcass | 0.0 |
| 11781_24hr_BF_carcass | 0.0 |
| 12072_36hr_BF_Fcarcass | 0.0 |
| 12073_48hr_BF_Fcarcass | 2.29775 |
| 12074_60hr_BF_Fcarcass | 0.0 |
| 11361_72hr_BF_Fcarcass | 0.0 |
| 11359_NBF_Ovaries | 0.0 |
| 12hr BF ovaries_11511_FPKM | 0.333042 |
| 11512_24hr_BF_Ovaries | 0.0939693 |
| 11513_36hr_BF_Ovaries | 0.183146 |
| 11514_48hr_BF_ovaries | 2.87647 |
| 11515_60hr_BF_ovaries | 1.29229 |
| 11360_72hr_BF_Ovaries | 0.265879 |
| 11362_0-2Hr_Embryos | 0.177219 |
| 11507_2-4hr_Embryos | 0.126809 |
| 11508_4-8hr_Embryos | 0.0 |
| 11509_8-12hr_Embryos | 0.0283946 |
| 11724_12-16hr_embryos | 0.0 |
| 11772_16-20hr_Embryos | 0.0 |
| 11773_20-24hr_embryos | 0.0376492 |
| 12105_24-28hr_Embryos | 0.198857 |
| 12170_28-32hr_embryos | 0.644156 |
| 12171_32-36hr_embryos | 0.541145 |
| 11774_36-40hr_embryos | 0.0893185 |
| 11775_40-44hr_embryos | 0.0 |
| 11776_44-48hr_embryos | 0.0 |
| 12172_48-52hr_embryos | 0.251683 |
| 12173_52-56hr_embryos | 0.378272 |
| 12106_56-60hr_Embryos | 0.0388333 |
| 11777_60-64hr_embryos | 0.0 |
| 12174_64-68hr_embryos | 0.220894 |
| 12175_68-72hr_embryos | 0.247346 |
| 12107_72-76hr_embryos | 0.0 |
| 11782_1st_Instar_larvae | 0.0 |
| 11783_2nd_Instar_larvae | 0.100989 |
| 11784_3rd_Instar_Larvae | 0.0 |
| 11363_4th_instar_larvae | 0.0 |
| 11725_MalePupae | 0.247346 |
| 11666_FemalePupae | 0.186683 |Enzymes

## Slide 27
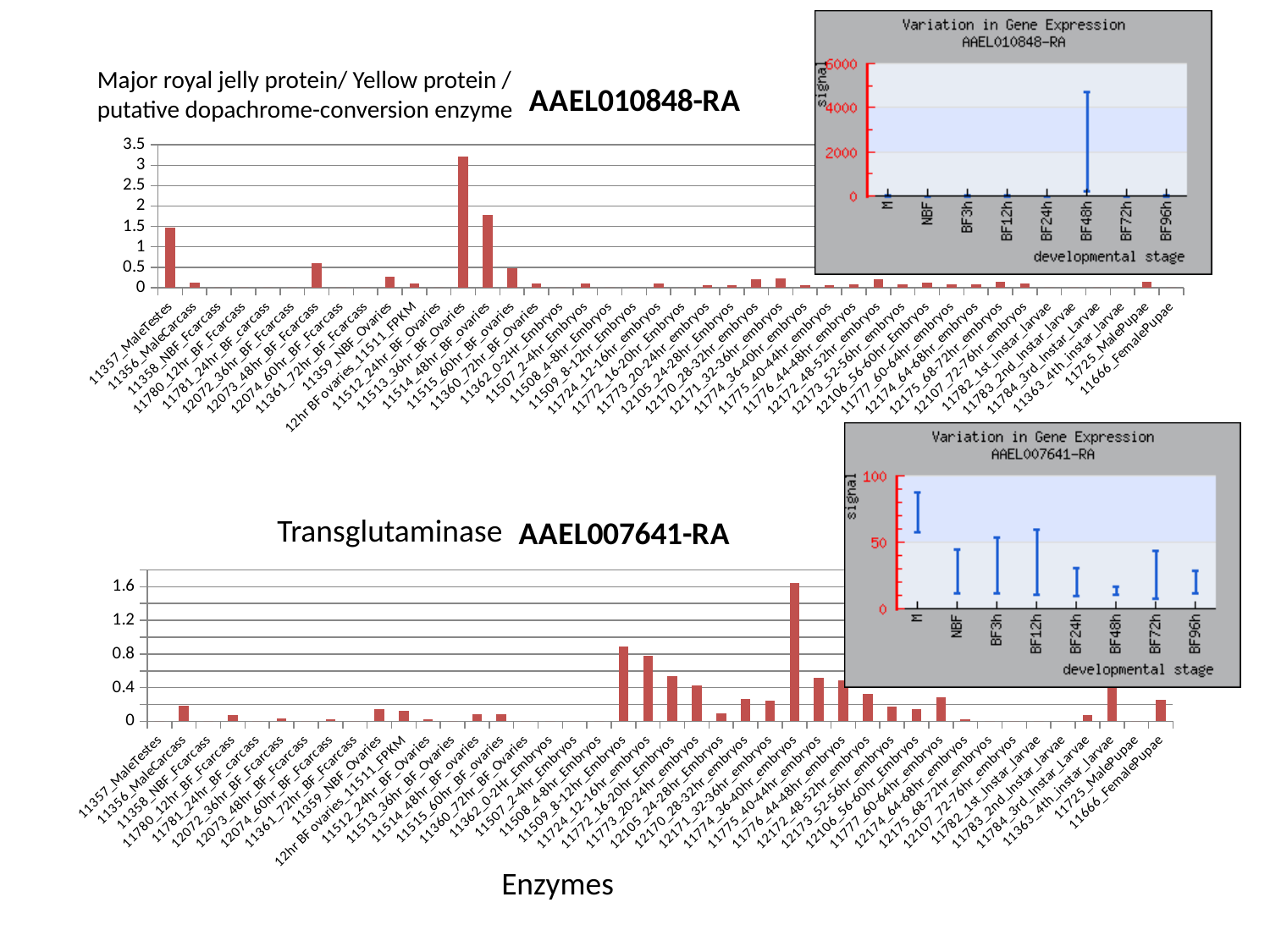

Major royal jelly protein/ Yellow protein / putative dopachrome-conversion enzyme
### Chart:
| Category | AAEL010848-RA |
|---|---|
| 11357_MaleTestes | 1.47368 |
| 11356_MaleCarcass | 0.124203 |
| 11358_NBF_Fcarcass | 0.0 |
| 11780_12hr_BF_Fcarcass | 0.0 |
| 11781_24hr_BF_carcass | 0.0 |
| 12072_36hr_BF_Fcarcass | 0.0 |
| 12073_48hr_BF_Fcarcass | 0.606527 |
| 12074_60hr_BF_Fcarcass | 0.0 |
| 11361_72hr_BF_Fcarcass | 0.0 |
| 11359_NBF_Ovaries | 0.264139 |
| 12hr BF ovaries_11511_FPKM | 0.109106 |
| 11512_24hr_BF_Ovaries | 0.0 |
| 11513_36hr_BF_Ovaries | 3.21697 |
| 11514_48hr_BF_ovaries | 1.77358 |
| 11515_60hr_BF_ovaries | 0.469472 |
| 11360_72hr_BF_Ovaries | 0.10658 |
| 11362_0-2Hr_Embryos | 0.0 |
| 11507_2-4hr_Embryos | 0.0922351 |
| 11508_4-8hr_Embryos | 0.0 |
| 11509_8-12hr_Embryos | 0.0 |
| 11724_12-16hr_embryos | 0.102155 |
| 11772_16-20hr_Embryos | 0.0 |
| 11773_20-24hr_embryos | 0.0618133 |
| 12105_24-28hr_Embryos | 0.0645975 |
| 12170_28-32hr_embryos | 0.197489 |
| 12171_32-36hr_embryos | 0.221748 |
| 11774_36-40hr_embryos | 0.0650074 |
| 11775_40-44hr_embryos | 0.0649056 |
| 11776_44-48hr_embryos | 0.0709274 |
| 12172_48-52hr_embryos | 0.204842 |
| 12173_52-56hr_embryos | 0.068831 |
| 12106_56-60hr_Embryos | 0.127854 |
| 11777_60-64hr_embryos | 0.076824 |
| 12174_64-68hr_embryos | 0.0716512 |
| 12175_68-72hr_embryos | 0.133794 |
| 12107_72-76hr_embryos | 0.0951458 |
| 11782_1st_Instar_larvae | 0.0 |
| 11783_2nd_Instar_larvae | 0.0 |
| 11784_3rd_Instar_Larvae | 0.0 |
| 11363_4th_instar_larvae | 0.0 |
| 11725_MalePupae | 0.133794 |
| 11666_FemalePupae | 0.0 |
### Chart:
| Category | AAEL007641-RA |
|---|---|
| 11357_MaleTestes | 0.0 |
| 11356_MaleCarcass | 0.180934 |
| 11358_NBF_Fcarcass | 0.0 |
| 11780_12hr_BF_Fcarcass | 0.0764159 |
| 11781_24hr_BF_carcass | 0.0 |
| 12072_36hr_BF_Fcarcass | 0.0303139 |
| 12073_48hr_BF_Fcarcass | 0.0 |
| 12074_60hr_BF_Fcarcass | 0.0214758 |
| 11361_72hr_BF_Fcarcass | 0.0 |
| 11359_NBF_Ovaries | 0.145118 |
| 12hr BF ovaries_11511_FPKM | 0.119712 |
| 11512_24hr_BF_Ovaries | 0.0250231 |
| 11513_36hr_BF_Ovaries | 0.0 |
| 11514_48hr_BF_ovaries | 0.0847537 |
| 11515_60hr_BF_ovaries | 0.0860211 |
| 11360_72hr_BF_Ovaries | 0.0 |
| 11362_0-2Hr_Embryos | 0.0 |
| 11507_2-4hr_Embryos | 0.0 |
| 11508_4-8hr_Embryos | 0.0 |
| 11509_8-12hr_Embryos | 0.884561 |
| 11724_12-16hr_embryos | 0.783348 |
| 11772_16-20hr_Embryos | 0.536938 |
| 11773_20-24hr_embryos | 0.428546 |
| 12105_24-28hr_Embryos | 0.0941325 |
| 12170_28-32hr_embryos | 0.265063 |
| 12171_32-36hr_embryos | 0.243153 |
| 11774_36-40hr_embryos | 1.64095 |
| 11775_40-44hr_embryos | 0.521537 |
| 11776_44-48hr_embryos | 0.491039 |
| 12172_48-52hr_embryos | 0.324025 |
| 12173_52-56hr_embryos | 0.175654 |
| 12106_56-60hr_Embryos | 0.139587 |
| 11777_60-64hr_embryos | 0.27981 |
| 12174_64-68hr_embryos | 0.0261411 |
| 12175_68-72hr_embryos | 0.0 |
| 12107_72-76hr_embryos | 0.0 |
| 11782_1st_Instar_larvae | 0.0 |
| 11783_2nd_Instar_larvae | 0.0 |
| 11784_3rd_Instar_Larvae | 0.0752885 |
| 11363_4th_instar_larvae | 0.399568 |
| 11725_MalePupae | 0.0 |
| 11666_FemalePupae | 0.25052 |Transglutaminase
Enzymes

## Slide 28
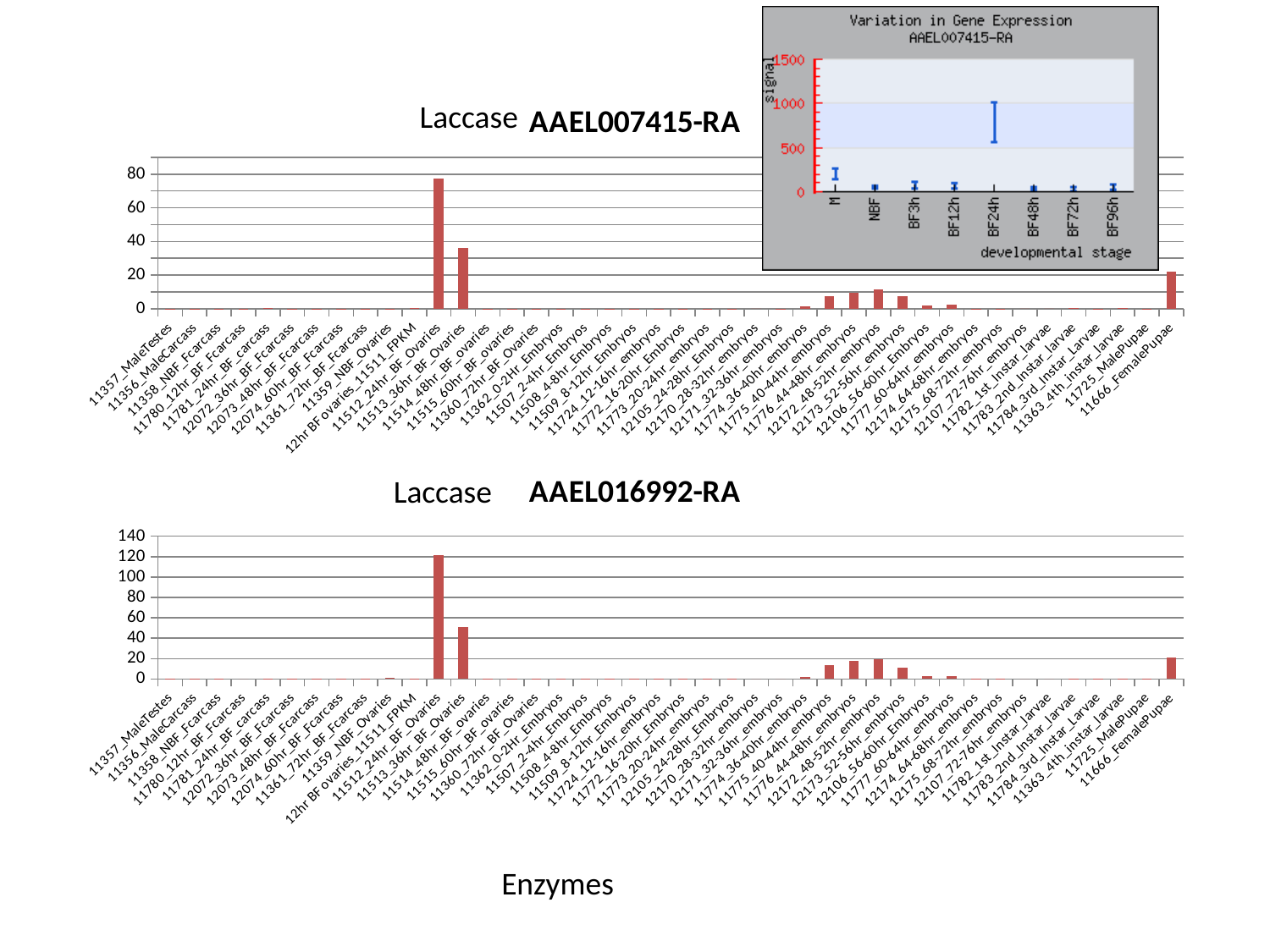

### Chart:
| Category | AAEL007415-RA |
|---|---|
| 11357_MaleTestes | 0.0146715 |
| 11356_MaleCarcass | 0.0655269 |
| 11358_NBF_Fcarcass | 0.0364549 |
| 11780_12hr_BF_Fcarcass | 0.0769662 |
| 11781_24hr_BF_carcass | 0.188913 |
| 12072_36hr_BF_Fcarcass | 0.0663376 |
| 12073_48hr_BF_Fcarcass | 0.0286537 |
| 12074_60hr_BF_Fcarcass | 0.0132195 |
| 11361_72hr_BF_Fcarcass | 0.0380645 |
| 11359_NBF_Ovaries | 0.107106 |
| 12hr BF ovaries_11511_FPKM | 0.242461 |
| 11512_24hr_BF_Ovaries | 77.4696 |
| 11513_36hr_BF_Ovaries | 36.2769 |
| 11514_48hr_BF_ovaries | 0.0485127 |
| 11515_60hr_BF_ovaries | 0.0157295 |
| 11360_72hr_BF_Ovaries | 0.0421673 |
| 11362_0-2Hr_Embryos | 0.0563258 |
| 11507_2-4hr_Embryos | 0.0431652 |
| 11508_4-8hr_Embryos | 0.0406549 |
| 11509_8-12hr_Embryos | 0.0446326 |
| 11724_12-16hr_embryos | 0.115891 |
| 11772_16-20hr_Embryos | 0.0263544 |
| 11773_20-24hr_embryos | 0.0305508 |
| 12105_24-28hr_Embryos | 0.00657576 |
| 12170_28-32hr_embryos | 2.28101e-22 |
| 12171_32-36hr_embryos | 0.00999916 |
| 11774_36-40hr_embryos | 1.19412 |
| 11775_40-44hr_embryos | 7.20696 |
| 11776_44-48hr_embryos | 9.66229 |
| 12172_48-52hr_embryos | 11.4303 |
| 12173_52-56hr_embryos | 7.36558 |
| 12106_56-60hr_Embryos | 1.97686 |
| 11777_60-64hr_embryos | 2.21756 |
| 12174_64-68hr_embryos | 0.0914698 |
| 12175_68-72hr_embryos | 0.108889 |
| 12107_72-76hr_embryos | 1.5324e-17 |
| 11782_1st_Instar_larvae | 3.38057e-24 |
| 11783_2nd_Instar_larvae | 0.371645 |
| 11784_3rd_Instar_Larvae | 0.0166179 |
| 11363_4th_instar_larvae | 0.210704 |
| 11725_MalePupae | 0.108889 |
| 11666_FemalePupae | 22.2356 |Laccase
### Chart:
| Category | AAEL016992-RA |
|---|---|
| 11357_MaleTestes | 0.0167715 |
| 11356_MaleCarcass | 0.0380901 |
| 11358_NBF_Fcarcass | 2.18494e-09 |
| 11780_12hr_BF_Fcarcass | 0.0 |
| 11781_24hr_BF_carcass | 0.537955 |
| 12072_36hr_BF_Fcarcass | 0.0781446 |
| 12073_48hr_BF_Fcarcass | 0.0438093 |
| 12074_60hr_BF_Fcarcass | 0.0677999 |
| 11361_72hr_BF_Fcarcass | 0.0780845 |
| 11359_NBF_Ovaries | 0.702804 |
| 12hr BF ovaries_11511_FPKM | 0.530414 |
| 11512_24hr_BF_Ovaries | 121.261 |
| 11513_36hr_BF_Ovaries | 50.5885 |
| 11514_48hr_BF_ovaries | 0.090307 |
| 11515_60hr_BF_ovaries | 0.16199 |
| 11360_72hr_BF_Ovaries | 0.201138 |
| 11362_0-2Hr_Embryos | 0.109061 |
| 11507_2-4hr_Embryos | 0.0479654 |
| 11508_4-8hr_Embryos | 0.118625 |
| 11509_8-12hr_Embryos | 0.108745 |
| 11724_12-16hr_embryos | 0.0942078 |
| 11772_16-20hr_Embryos | 0.00770483 |
| 11773_20-24hr_embryos | 0.0284817 |
| 12105_24-28hr_Embryos | 0.00742935 |
| 12170_28-32hr_embryos | 0.0 |
| 12171_32-36hr_embryos | 0.0 |
| 11774_36-40hr_embryos | 2.2073 |
| 11775_40-44hr_embryos | 13.7309 |
| 11776_44-48hr_embryos | 17.589 |
| 12172_48-52hr_embryos | 19.399 |
| 12173_52-56hr_embryos | 11.1297 |
| 12106_56-60hr_Embryos | 2.7028 |
| 11777_60-64hr_embryos | 3.10936 |
| 12174_64-68hr_embryos | 0.138077 |
| 12175_68-72hr_embryos | 0.216499 |
| 12107_72-76hr_embryos | 0.0 |
| 11782_1st_Instar_larvae | 0.0 |
| 11783_2nd_Instar_larvae | 0.522618 |
| 11784_3rd_Instar_Larvae | 0.0872592 |
| 11363_4th_instar_larvae | 0.366116 |
| 11725_MalePupae | 0.216499 |
| 11666_FemalePupae | 20.7331 |Laccase
Enzymes

## Slide 29
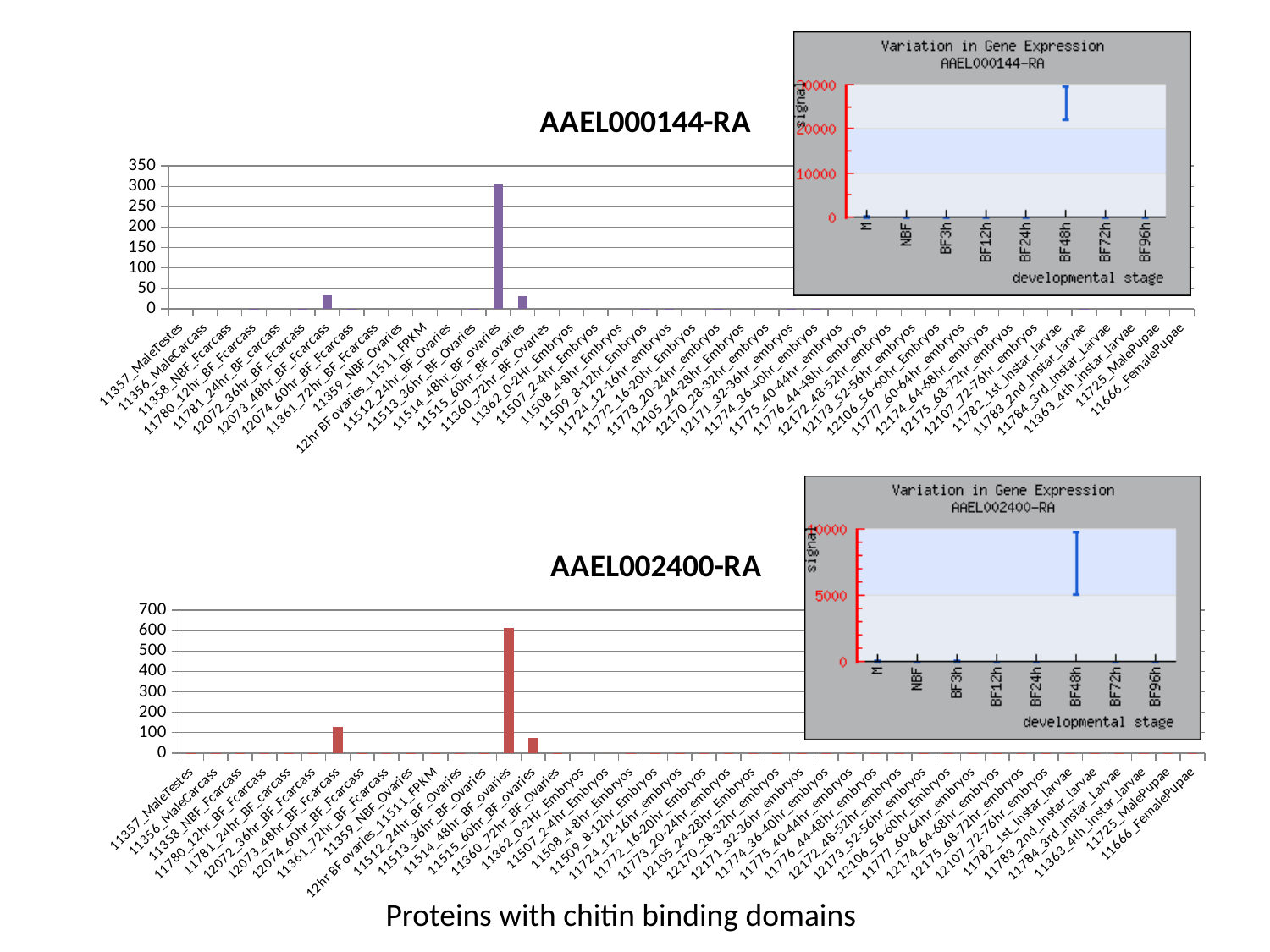

### Chart:
| Category | AAEL000144-RA |
|---|---|
| 11357_MaleTestes | 0.0 |
| 11356_MaleCarcass | 0.0 |
| 11358_NBF_Fcarcass | 0.0 |
| 11780_12hr_BF_Fcarcass | 0.0510743 |
| 11781_24hr_BF_carcass | 0.0 |
| 12072_36hr_BF_Fcarcass | 0.060783 |
| 12073_48hr_BF_Fcarcass | 32.9987 |
| 12074_60hr_BF_Fcarcass | 0.0430467 |
| 11361_72hr_BF_Fcarcass | 0.0 |
| 11359_NBF_Ovaries | 0.0 |
| 12hr BF ovaries_11511_FPKM | 0.0 |
| 11512_24hr_BF_Ovaries | 0.0 |
| 11513_36hr_BF_Ovaries | 0.109986 |
| 11514_48hr_BF_ovaries | 305.248 |
| 11515_60hr_BF_ovaries | 30.8554 |
| 11360_72hr_BF_Ovaries | 0.0 |
| 11362_0-2Hr_Embryos | 0.0 |
| 11507_2-4hr_Embryos | 0.0 |
| 11508_4-8hr_Embryos | 0.0 |
| 11509_8-12hr_Embryos | 0.0682173 |
| 11724_12-16hr_embryos | 0.0747955 |
| 11772_16-20hr_Embryos | 0.0 |
| 11773_20-24hr_embryos | 0.0452256 |
| 12105_24-28hr_Embryos | 0.0 |
| 12170_28-32hr_embryos | 0.0 |
| 12171_32-36hr_embryos | 0.0541628 |
| 11774_36-40hr_embryos | 0.0953711 |
| 11775_40-44hr_embryos | 0.0 |
| 11776_44-48hr_embryos | 0.0 |
| 12172_48-52hr_embryos | 0.0 |
| 12173_52-56hr_embryos | 0.0 |
| 12106_56-60hr_Embryos | 0.0 |
| 11777_60-64hr_embryos | 0.0 |
| 12174_64-68hr_embryos | 0.0 |
| 12175_68-72hr_embryos | 0.0 |
| 12107_72-76hr_embryos | 0.0 |
| 11782_1st_Instar_larvae | 0.0 |
| 11783_2nd_Instar_larvae | 0.0539087 |
| 11784_3rd_Instar_Larvae | 0.0 |
| 11363_4th_instar_larvae | 0.0 |
| 11725_MalePupae | 0.0 |
| 11666_FemalePupae | 0.0 |
### Chart:
| Category | AAEL002400-RA |
|---|---|
| 11357_MaleTestes | 0.0841747 |
| 11356_MaleCarcass | 0.250843 |
| 11358_NBF_Fcarcass | 0.156419 |
| 11780_12hr_BF_Fcarcass | 0.161015 |
| 11781_24hr_BF_carcass | 0.0940046 |
| 12072_36hr_BF_Fcarcass | 0.221536 |
| 12073_48hr_BF_Fcarcass | 126.385 |
| 12074_60hr_BF_Fcarcass | 0.374505 |
| 11361_72hr_BF_Fcarcass | 0.114727 |
| 11359_NBF_Ovaries | 0.0383213 |
| 12hr BF ovaries_11511_FPKM | 0.0316124 |
| 11512_24hr_BF_Ovaries | 0.0198242 |
| 11513_36hr_BF_Ovaries | 0.0434628 |
| 11514_48hr_BF_ovaries | 615.294 |
| 11515_60hr_BF_ovaries | 74.4985 |
| 11360_72hr_BF_Ovaries | 0.12487 |
| 11362_0-2Hr_Embryos | 0.0 |
| 11507_2-4hr_Embryos | 0.0 |
| 11508_4-8hr_Embryos | 0.0246019 |
| 11509_8-12hr_Embryos | 0.409598 |
| 11724_12-16hr_embryos | 0.120668 |
| 11772_16-20hr_Embryos | 0.0648236 |
| 11773_20-24hr_embryos | 0.164963 |
| 12105_24-28hr_Embryos | 0.255592 |
| 12170_28-32hr_embryos | 0.101792 |
| 12171_32-36hr_embryos | 0.0614708 |
| 11774_36-40hr_embryos | 0.437308 |
| 11775_40-44hr_embryos | 0.282393 |
| 11776_44-48hr_embryos | 0.187168 |
| 12172_48-52hr_embryos | 0.143936 |
| 12173_52-56hr_embryos | 0.0198795 |
| 12106_56-60hr_Embryos | 0.0866963 |
| 11777_60-64hr_embryos | 0.346161 |
| 12174_64-68hr_embryos | 0.043391 |
| 12175_68-72hr_embryos | 0.103472 |
| 12107_72-76hr_embryos | 0.0138008 |
| 11782_1st_Instar_larvae | 0.355042 |
| 11783_2nd_Instar_larvae | 0.241357 |
| 11784_3rd_Instar_Larvae | 0.100316 |
| 11363_4th_instar_larvae | 0.0316542 |
| 11725_MalePupae | 0.103472 |
| 11666_FemalePupae | 0.160635 |Proteins with chitin binding domains

## Slide 30
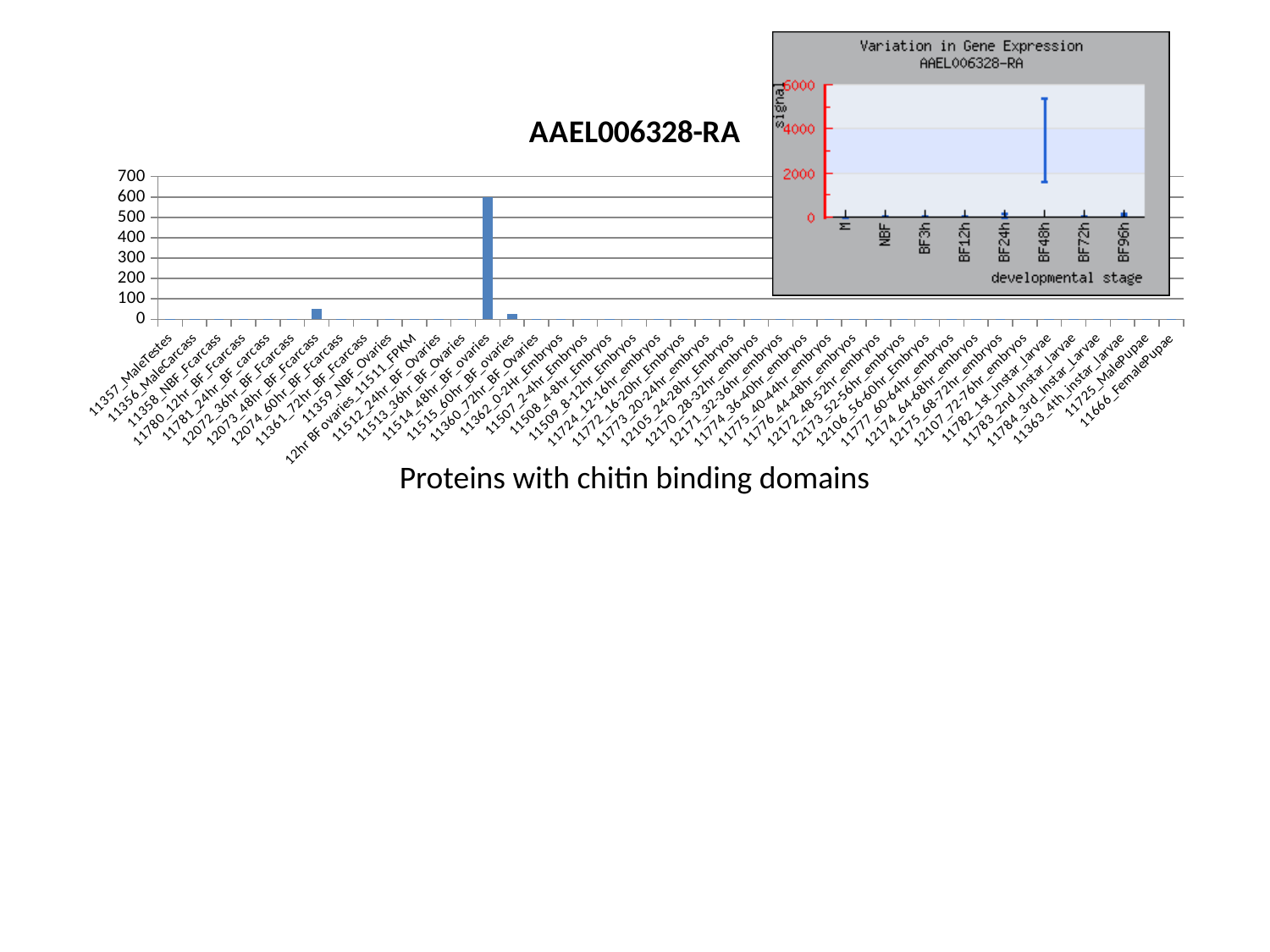

### Chart:
| Category | AAEL006328-RA |
|---|---|
| 11357_MaleTestes | 0.188043 |
| 11356_MaleCarcass | 0.266838 |
| 11358_NBF_Fcarcass | 0.723129 |
| 11780_12hr_BF_Fcarcass | 0.991745 |
| 11781_24hr_BF_carcass | 0.485875 |
| 12072_36hr_BF_Fcarcass | 0.286125 |
| 12073_48hr_BF_Fcarcass | 50.4535 |
| 12074_60hr_BF_Fcarcass | 1.6216 |
| 11361_72hr_BF_Fcarcass | 0.499307 |
| 11359_NBF_Ovaries | 0.171216 |
| 12hr BF ovaries_11511_FPKM | 3.01315 |
| 11512_24hr_BF_Ovaries | 1.978 |
| 11513_36hr_BF_Ovaries | 3.1393 |
| 11514_48hr_BF_ovaries | 600.891 |
| 11515_60hr_BF_ovaries | 26.7937 |
| 11360_72hr_BF_Ovaries | 2.89949 |
| 11362_0-2Hr_Embryos | 2.6166 |
| 11507_2-4hr_Embryos | 1.75279 |
| 11508_4-8hr_Embryos | 1.28239 |
| 11509_8-12hr_Embryos | 1.08378 |
| 11724_12-16hr_embryos | 0.308075 |
| 11772_16-20hr_Embryos | 0.316751 |
| 11773_20-24hr_embryos | 0.133057 |
| 12105_24-28hr_Embryos | 0.194356 |
| 12170_28-32hr_embryos | 0.199005 |
| 12171_32-36hr_embryos | 0.0956263 |
| 11774_36-40hr_embryos | 0.224471 |
| 11775_40-44hr_embryos | 0.363605 |
| 11776_44-48hr_embryos | 0.182952 |
| 12172_48-52hr_embryos | 0.323473 |
| 12173_52-56hr_embryos | 0.177636 |
| 12106_56-60hr_Embryos | 0.32938 |
| 11777_60-64hr_embryos | 0.528211 |
| 12174_64-68hr_embryos | 0.431789 |
| 12175_68-72hr_embryos | 0.491258 |
| 12107_72-76hr_embryos | 0.287744 |
| 11782_1st_Instar_larvae | 0.576532 |
| 11783_2nd_Instar_larvae | 1.07868 |
| 11784_3rd_Instar_Larvae | 1.65812 |
| 11363_4th_instar_larvae | 2.21571 |
| 11725_MalePupae | 0.491258 |
| 11666_FemalePupae | 0.211124 |Proteins with chitin binding domains
