# Supplementary figures and images for: Integrated proteomic and transcriptomic analysis of the Aedes aegypti eggshell
Source: BMC Dev Biol. 2014 Apr 5;14:15. doi: 10.1186/1471-213X-14-15 (PMC4234484; doi:10.1186/1471-213X-14-15)

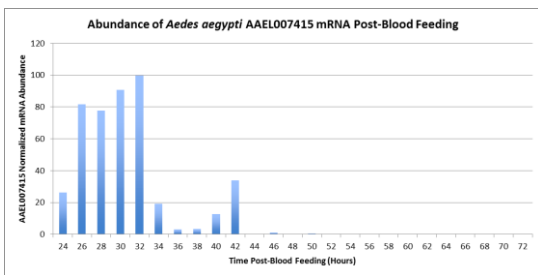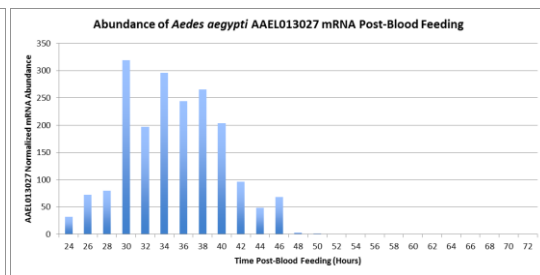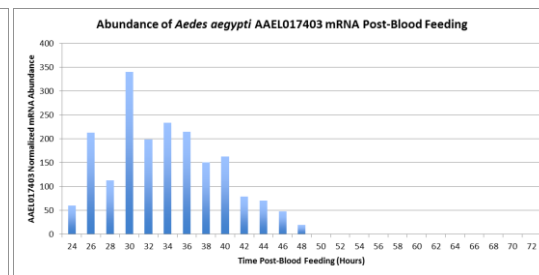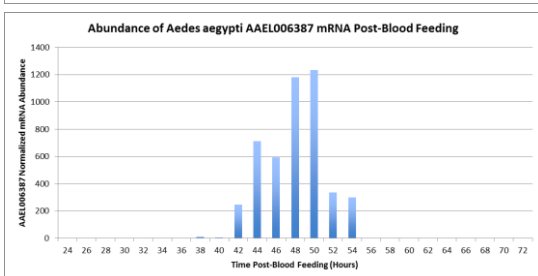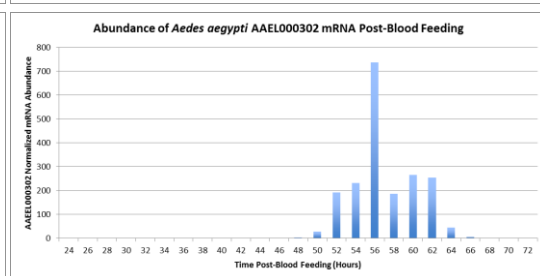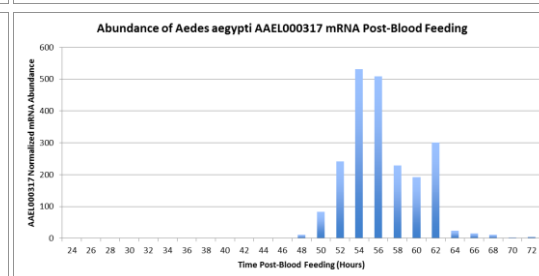

Supplement: Additional file 5 — Quantitative RT-PCR determinations of transcript accumulation in the ovaries of Aedes aegypti blood-fed females. Bars represent the mean of three biological replicates with similar results. RNA for each biological replicate was isolated from the ovaries of a single female. AAEL007415 (Laccase), AAEL013027 (VMP 15a-1), AAEL017403 (VMP 15a-2), AAEL006387 (AaegOBP29), AAEL000302 (Cysteine-rich protein, AaegCysR3), AAEL000317 (Cysteine-rich protein, AaegCysR4). Values were normalized to the expression of the ribosomal protein 49 gene, AAEL003396. [file 1471-213X-14-15-S5.pdf]
