# Supplementary material for: Integrated proteomic and transcriptomic analysis of the Aedes aegypti eggshell
Source: BMC Dev Biol. 2014 Apr 5;14:15. doi: 10.1186/1471-213X-14-15 (PMC4234484; doi:10.1186/1471-213X-14-15)

Additional file 6. Sequence and hydrophobicity conservation among *Aedes aegypti* vitelline membrane proteins. A) Full length protein sequences were submitted to MEME http://meme.nbcr.net/meme/cgi-bin/meme.cgi and a conserved motif 30 amino acids in length was discovered. B) Multiple alignment of the sequence motif constructed at PRALINE http://www.ibi.vu.nl/programs/pralinewww/ provides additional support for the conservation. C) The hydrophobicity patterns of the six vitelline membrane proteins were predicted at http://web.expasy.org/cgi-bin/protscale/protscale.pl using the Hphob. / Kyte & Doolittle option. Bars were inserted in the graphs to indicate the positions of the conserved motif displayed in A and B.

A

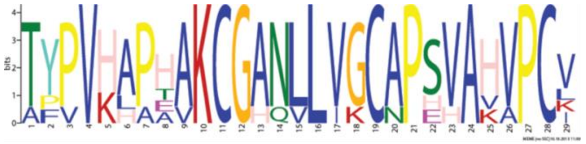

B

Unconserved 0 1 2 3 4 5 6 7 8 9 10 **Conserved**

```
           ..........10..........20..........30
AAEL013027 TYP-VHAPHA KCGANLLVGC APSVAHVPCV
AAEL006670 TYP-VHAPHA KCGANLLVGC APSVAHVPCL
AAEL014561 TYP-VHAPHA KCGANLLVGC APSVAHAPCV
AAEL017403 TYP-VKAPAA KCGANLLVGC APSVAHVPCV
AAEL017501 -APVVHHATA KCGANVLVGC APEVAKVPCI
AAEL017471 TFP-VKLPEV KCGHQLLIKC NPHHAVVPCK
Consistency 66*0*65748 ***788*97* 7*67*58**6
```

C

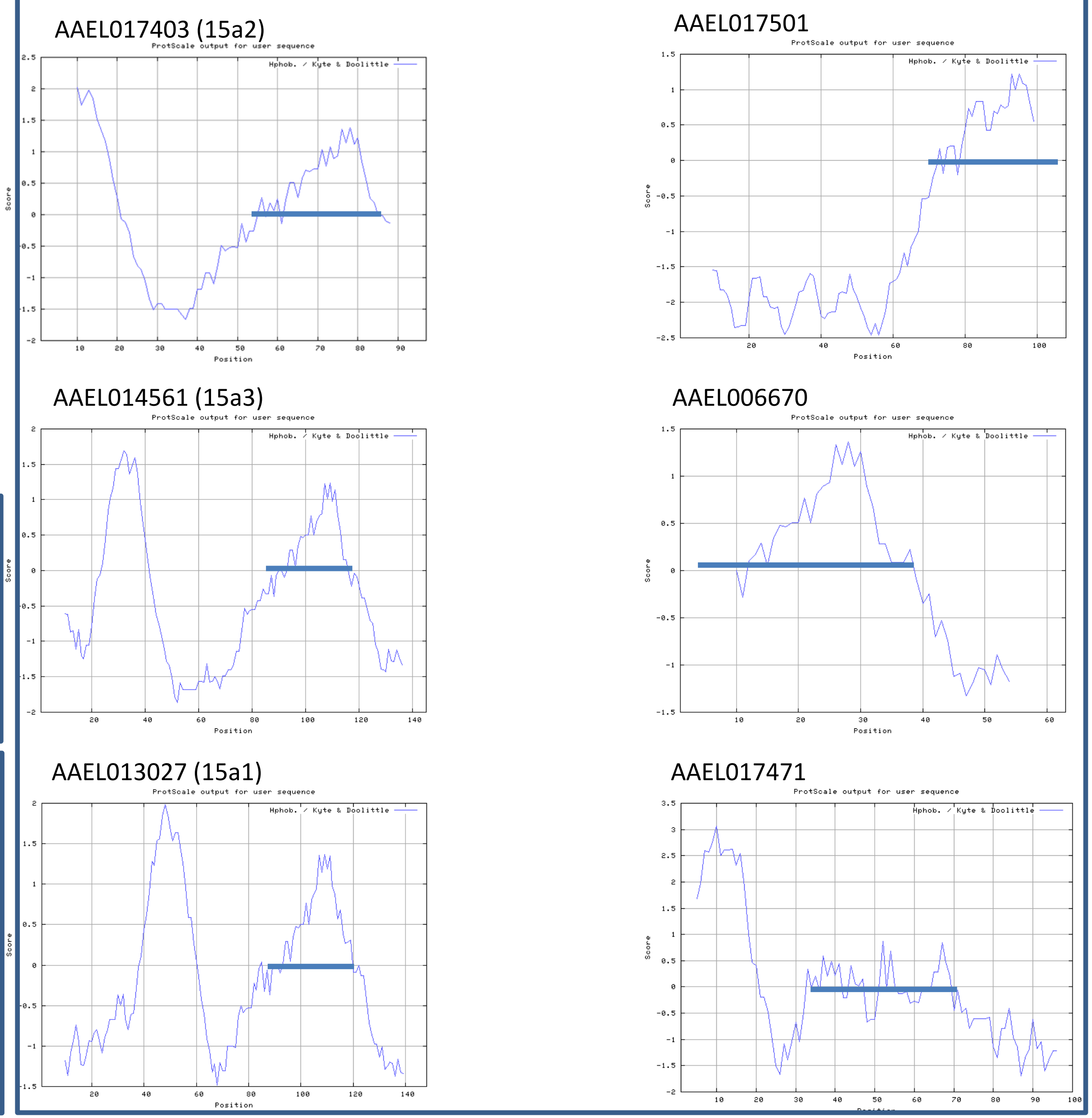

Supplement: Additional file 6 — Sequence and hydrophobicity conservation among Aedes aegypti vitelline membrane proteins. A) Full-length protein sequences were submitted to MEME [65]http://meme.nbcr.net/meme/cgi-bin/meme.cgi and a conserved motif 30 amino acids in length was identified. B) Multiple alignment of the sequence motif constructed at PRALINE [66]http://www.ibi.vu.nl/programs/pralinewww/ provides additional support for its conservation. C) The hydrophobicity patterns of the six vitelline membrane proteins were predicted at ExPASy [67]http://web.expasy.org/cgi-bin/protscale/protscale.pl using the Hphob./Kyte & Doolittle option [68]. Bars were inserted in the graphs to indicate the positions of the conserved motif displayed in A and B. [file 1471-213X-14-15-S6.pdf]
