# Supplementary material for: Integrated proteomic and transcriptomic analysis of the Aedes aegypti eggshell
Source: BMC Dev Biol. 2014 Apr 5;14:15. doi: 10.1186/1471-213X-14-15 (PMC4234484; doi:10.1186/1471-213X-14-15)

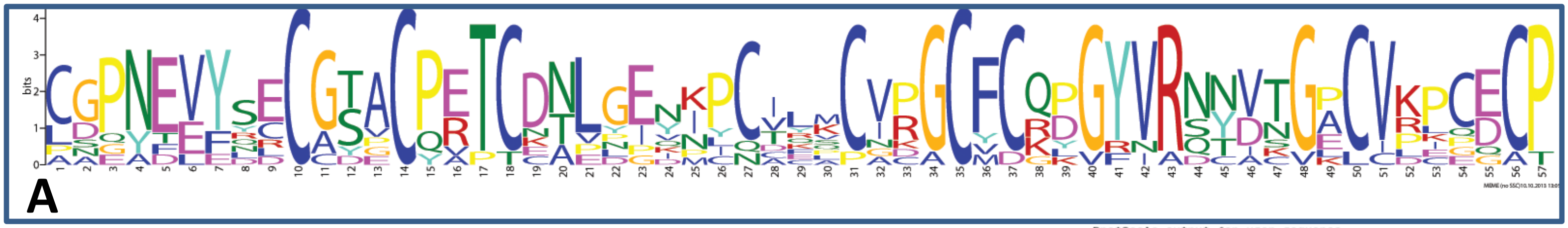

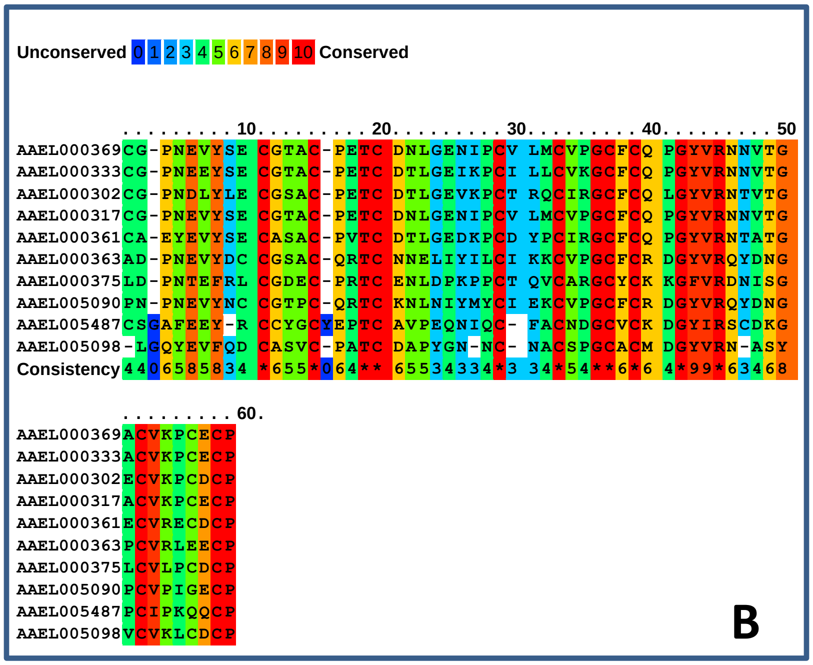

Additional file 7. Sequence and hydrophobicity conservation among *Aedes aegypti* eggshell cysteine-rich proteins. A) Full length protein sequences were submitted to MEME http://meme.nbcr.net/meme/cgi-bin/meme.cgi and a conserved motif 50 amino acids in length was discovered. B) Multiple alignment of the sequence motif constructed at PRALINE http://www.ibi.vu.nl/programs/pralinewww/ supports the conservation. C) The hydrophobicity patterns of the cysteine-rich proteins were predicted at http://web.expasy.org/cgi-bin/protscale/protscale.pl using the Hphob. / Kyte & Doolittle option. Bars were inserted in the graphs to indicate the positions of the conserved motif displayed in A and B.

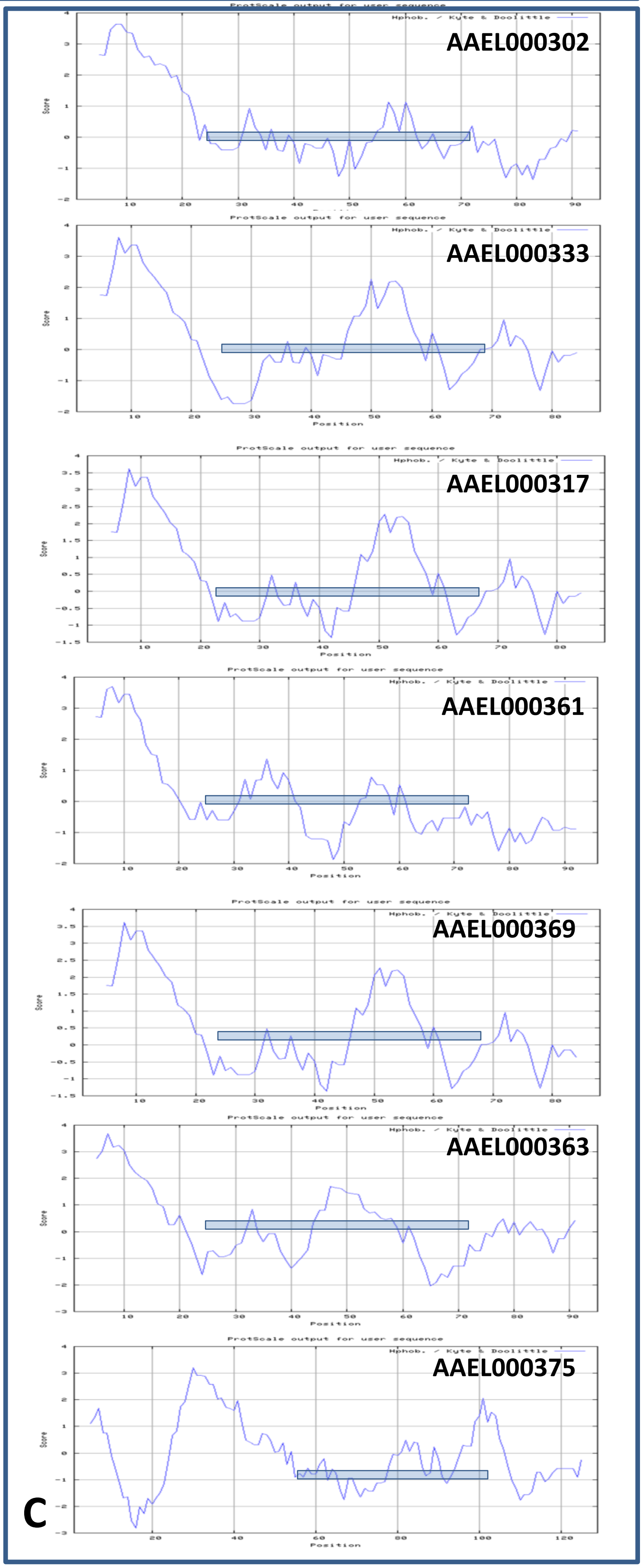

Supplement: Additional file 7 — Sequence and hydrophobicity conservation among Aedes aegypti eggshell cysteine-rich proteins. A) Full length protein sequences were submitted to MEME [65]http://meme.nbcr.net/meme/cgi-bin/meme.cgi and a conserved motif 50 amino acids in length was discovered. B) Multiple alignment of the sequence motif constructed at PRALINE [66]http://www.ibi.vu.nl/programs/pralinewww/ supports the proposed conservation. C) The hydrophobicity patterns of the cysteine-rich proteins were predicted at ExPASy [67]http://web.expasy.org/cgi-bin/protscale/protscale.pl using the Hphob./Kyte & Doolittle option [68]. Bars were inserted in the graphs to indicate the positions of the conserved motif displayed in A and B. [file 1471-213X-14-15-S7.pdf]
