# Supplementary material for: Integrated proteomic and transcriptomic analysis of the Aedes aegypti eggshell
Source: BMC Dev Biol. 2014 Apr 5;14:15. doi: 10.1186/1471-213X-14-15 (PMC4234484; doi:10.1186/1471-213X-14-15)
Supplement: Additional file 8 — Oligonucleotide primer sequences used in quantitative RT-PCRs and for the generation of probes for hybridizations in situ (q and h suffixes, respectively). [file 1471-213X-14-15-S8.pdf]

Additional file 8. Oligonucleotide primer sequences used in quantitative RT-PCRs and for the generation of probes for hybridizations *in situ* (q and h suffixes, respectively).

Primer ID	Sequence 5'-3'
AAEL017403Fq	ACCCCGTTGTCCACACATAC
AAEL017403Rq	AGCGCATCCTACCAGAAGGT
AAEL013027Fq	GTCCCACTTGAAGGCTCTTG
AAEL000302Rq	TCCAGTCACGGTATTGCGTA
AAEL000302Fq	TACGCAATACCGTGACTGGA
AAEL000302Rq	CACTGGCGTTGTTGTAGTGG
AAEL000317Fq	CGGAATGTGGAACAGCTTG
AAEL000317Rq	CGTACGTAACCTGGCTGACA
AAEL007415Fq	GCTTCCAGTGCAAGTCAACA
AAEL007415Rq	GTATGGACCACGGGCATATC
AAEL006387Fq	TCAGGGAGTCTTCCCAGATG
AAEL006387Rq	CCGTCGTAGAGTCCTTCAGC
RP49Fq	ACAAGCTTGCCCCCAACT
RP49Rq	CCGTAACCGATGTTTGGC
AAEL006387-Rh	GCCAGGAATTGGATGAGTGT
AAEL006387-Fh	CCGTCGTAGAGTCCTTCAGC
AAEL006396-Fh	GCTTCCGACAACAGTTGGAT
AAEL006396-Rh	AAGCAGCGCCATCATAAAGT
AAEL007112-Fh	CCAACTCCTCCAACCACATT
AAEL007112-Rh	TCAACCGTGGTTTCTTCCTC
AAEL010714-Fh	ACAAGTTACGCCGAAGCACT
AAEL010714-Rh	ACAGCTGTCAAGCGATTCCT
